# The effect of SSRIs on fear learning: a systematic review and meta-analysis

**DOI:** 10.1007/s00213-023-06333-7

**Published:** 2023-02-27

**Authors:** Elise J. Heesbeen, Elisabeth Y. Bijlsma, P. Monika Verdouw, Caspar van Lissa, Carlijn Hooijmans, Lucianne Groenink

**Affiliations:** 1 Division of Pharmacology, Utrecht Institute for Pharmaceutical Sciences, Utrecht University, Utrecht, Netherlands; 2 Department of Methodology, Tilburg University, Tilburg, Netherlands; 3 Department of Anaesthesiology, Pain and Palliative Care, Radboud University Medical Center, Nijmegen, Netherlands

**Keywords:** Fear learning, Anxiety, SSRIs, Acquisition, Extinction, Systematic review, Meta-analysis, 5-HT re-uptake inhibitor, Fear conditioning, Contextual anxiety

## Abstract

**Rationale:**

Selective serotonin reuptake inhibitors (SSRIs) are considered first-line medication for anxiety-like disorders such as panic disorder, generalized anxiety disorder, and post-traumatic stress disorder. Fear learning plays an important role in the development and treatment of these disorders. Yet, the effect of SSRIs on fear learning are not well known.

**Objective:**

We aimed to systematically review the effect of six clinically effective SSRIs on acquisition, expression, and extinction of cued and contextual conditioned fear.

**Methods:**

We searched the Medline and Embase databases, which yielded 128 articles that met the inclusion criteria and reported on 9 human and 275 animal experiments.

**Results:**

Meta-analysis showed that SSRIs significantly reduced contextual fear expression and facilitated extinction learning to cue. Bayesian-regularized meta-regression further suggested that chronic treatment exerts a stronger anxiolytic effect on cued fear expression than acute treatment. Type of SSRI, species, disease-induction model, and type of anxiety test used did not seem to moderate the effect of SSRIs. The number of studies was relatively small, the level of heterogeneity was high, and publication bias has likely occurred which may have resulted in an overestimation of the overall effect sizes.

**Conclusions:**

This review suggests that the efficacy of SSRIs may be related to their effects on contextual fear expression and extinction to cue, rather than fear acquisition. However, these effects of SSRIs may be due to a more general inhibition of fear-related emotions. Therefore, additional meta-analyses on the effects of SSRIs on unconditioned fear responses may provide further insight into the actions of SSRIs.

**Supplementary Information:**

The online version contains supplementary material available at 10.1007/s00213-023-06333-7.

## Introduction

Fear learning is a central process underlying the development of anxiety-like disorders, such as panic disorder (PD) (Bouton et al. 2001), generalized anxiety disorder (GAD) (Lissek et al. 2014), and post-traumatic stress disorder (PTSD) (Orr et al. 2000). All three disorders are associated with a disproportional reaction of fear in the absence of actual danger. Patients with these disorders are often not capable to extinguish this learned fear which may result in excessive fear expression and fear generalization (Jovanovic et al. 2012; Michael et al. 2007; Milad et al. 2013, 2009; Singewald et al. 2015; Wessa and Flor 2007)(reviewed in: Holmes and Singewald (2013); Kong et al. (2014)). Fear learning is often divided into five processes. These are acquisition learning, fear expression after acquisition learning, consolidation, extinction learning, and fear expression after extinction learning. Although the clinical representation of the three anxiety-like disorders is quite different, several lines of evidence point towards involvement of fear learning deficits in the etiology of these disorders. GAD and PTSD patients show enhanced fear acquisition (Orr et al. 2000; Peri et al. 2000; Thayer et al. 2000). Impaired within-session fear extinction is observed in patients with PD (Michael et al. 2007; Otto et al. 2014) and GAD (Pitman and Orr 1986) whereas PTSD patients demonstrate impaired extinction recall (Milad et al. 2008). Abnormal conditioned fear generalization is evident in PD (Lissek et al. 2010), PTSD (Lis et al. 2020), and GAD patients (Lissek et al. 2014) compared to healthy controls. There is evidence for specific neural circuitry changes within these three anxiety disorders that may relate to these fear learning deficits. First, PTSD patients show an increased activation of the dorsal anterior cingulate cortex (dACC) (Milad et al. 2009) and abnormal activity and connectivity of the hippocampus (Huang et al. 2014a). These changes are associated with a reduced ability to extinguish learned fear (Rothbaum and Davis 2003) and the preservation and intrusion of traumatic memories (Chen and Etkin 2013), respectively. Second, both PTSD and PD patients exhibit increased sustained bed nucleus of the stria terminalis (BNST) activation which is associated with heightened sensitivity to unpredictable threat (Brinkmann et al. 2017a, b; Brinkmann et al. 2017a, b). Third, hyperactivity of the anterior insula is observed in PD (Brinkmann et al. 2017a, b) and GAD (Yassa et al. 2012) patients during sustained threat which might relate to feeling a loss of control and also reflects autonomic and emotional distress during threat (Alvarez et al. 2015; Hamann et al. 2002). Fourth, GAD patients exhibit a greater connectivity between the amygdala and the medial prefrontal cortex (mPFC) (Robinson et al. 2014) which suggests that GAD patients have an increased attention toward threats (Robinson et al. 2012; Robinson et al. 2016; Vytal et al. 2014). However, how these neural mechanisms exactly relate to the different fear processes and the clinical symptoms associated with these disorders is not yet fully understood. Since there are clear parallels in terms of neural circuits and fear learning processes between humans and experimental animals (reviewed in: Burghardt and Bauer (2013)), investigating fear learning in animals can provide valuable insight in the neural mechanisms underlying anxiety disorders.

Pharmacological intervention could potentially influence the aforementioned fear learning processes, eventually leading to a reduction in abnormal fear learning. Even though all processes appear crucial in etiology and treatment, the main focus of therapeutic action has been on reducing fear expression after extinction learning in order to support long-term extinction (Singewald et al. 2015). Selective serotonin reuptake inhibitors (SSRIs) are the first-line pharmacological treatment for anxiety disorders such as PD and GAD and PTSD. Currently, six SSRIs are available for clinical use: citalopram, escitalopram, fluoxetine, fluvoxamine, paroxetine, and sertraline (Baldwin et al. 2014; Koen and Stein 2011). Their effectiveness, safety, and absence of abuse potential have been confirmed by multiple randomized controlled trials (reviewed in: Ravindran and Stein (2010)). It is not yet clear how the SSRIs reduce anxiety symptoms; this could be via a specific effect on fear learning circuits (reviewed in: Burghardt and Bauer (2013)), but also via, for example, general emotional blunting (Fagiolini et al. 2021; Goodwin et al. 2017). Since the effects of SSRIs on the different fear learning processes are not well known and have not yet been systematically reviewed, this knowledge gap will be investigated in the current systematic review.

With this systematic review we aimed to objectively determine which fear learning processes are affected by clinically effective SSRIs. Since SSRIs have been shown beneficial in the treatment of anxiety-like disorders, the results of this systematic review may identify fear learning processes that could be particularly relevant to address in future research on the development and treatment of anxiety-like disorders. Furthermore, results from this systematic review may contribute to a better understanding of the role of the serotonergic system in acquisition and extinction of fear learning and fear expression. In this systematic review, we synthesized data from human and animal studies regarding the effects of SSRIs on fear acquisition and extinction learning and on fear expression after acquisition and extinction learning, for both cued and contextual fear. We selected these fear learning processes because serotonin transporters have been shown to play a role in acquisition learning (Bijlsma et al. 2015; Heitland et al. 2013) and SSRIs may affect the fear extinction process (Deschaux et al. 2011; Karpova et al. 2011). To improve translation between animal and human research in future studies, we also examined whether study characteristics, including species, use of a disease-induction model, duration of SSRI treatment, type of SSRI, and type of anxiety test used, may affect the effects of SSRIs. This information could prove useful to determine the most effective experimental set-up to study specific fear learning processes.

## Materials and methods

This systematic review was performed in collaboration with the Systematic Review Center for Laboratory animal Experimentation (SYRCLE). Furthermore, the reporting of this systematic review complied with the guidelines described in the Preferred Reporting Items for Systematic Reviews and Meta-Analysis (PRISMA) statement (Page et al. 2021).

### Study protocol

This systematic review was conducted based on a preregistered protocol (PROSPERO, CRD42020207075) which was registered at the PROSPERO website of the University of York on 30 September 2020 and updated on 25 February 2021 and 28 September 2021; it can be accessed through this website: https://www.crd.york.ac.uk/prospero/. The protocol can also be found in supplementary file S1.

### Literature search and selection

#### Search strategy

A literature search was performed in two major medical databases, Medline and Embase, containing all published articles up until the 4th of May 2021. The following components were included in the search strategy: (1) clinically effective SSRIs (citalopram, escitalopram, fluoxetine, fluvoxamine, paroxetine, and sertraline) and (2) fear learning processes (acquisition learning, fear expression after acquisition learning, extinction learning, and fear expression after extinction learning). The full search string can be found in supplementary file S2. The articles retrieved through this literature search were imported in EndNote and herein deduplicated. Afterwards the remaining set of articles was imported in the web program Rayyan (https://www.rayyan.ai/) to perform study selection.

#### Study selection

Two reviewers (EH and MV) independently screened all articles that were identified during the literature search, based on inclusion and exclusion criteria as documented in the pre-registered protocol. Screening occurred in two phases: (1) titles and abstracts of all unique studies were screened and all possible eligible articles were selected; (2) full text of possibly suitable studies were read to determine eligibility. Discrepancies in screening of studies in both phases were resolved by discussion between the two reviewers, if an agreement was not achieved a third reviewer was consulted (EYB or LG).

Study selection occurred in two phases: (1) title and abstract screening and (2) full-text screening. The following exclusion criteria were used during the title and abstract screening: (1) a non-original study (reviews, case reports, conference abstracts, etc.); (2) an in vitro, ex vivo study or non-animal or non-human-related research; (3) any other treatment than one of the six following SSRIs (fluoxetine, fluvoxamine, sertraline, citalopram, escitalopram, paroxetine) and/or a combined treatment with any other drug; (4) outcome not assessing the level of anxiety in tests for fear acquisition learning and expression and in tests for fear extinction learning and expression. These tests were limited to conditioned freezing, fear potentiated startle, several forms of passive avoidance (inhibitory avoidance, step-through avoidance, plus-maze discriminative avoidance task, conditioned odor avoidance task), several forms of active avoidance (avoidance conditioning, two-way active avoidance), conditioned emotional response, and conditioned place aversion; (5) the SSRI is not given directly to the study subject. The full-text screening used both the previous and the following exclusion criteria: (6) no appropriate placebo or vehicle control group is included; (7) information on the protocol of fear learning is not available/retrievable within one reference; (8) information on specific SSRI used is not available/retrievable; (9) results from the fear learning test are not reported; (10) additional pharmacological treatment is used before/during/after SSRI treatment and before testing fear learning; (11) the effect of the SSRI treatment is not tested on fear learning; (12) the acquisition and/or extinction studies did not give the SSRI up until or within 24 h of the measurement of acquisition/extinction learning or fear expression (after acquisition or extinction learning); (13) the full article text is not retrievable; and (14) no English or Dutch version is retrievable.

### Extraction of study characteristics

The study characteristics as shown in Table 1 were extracted from the included articles by one reviewer (EH) and checked by the second reviewer (MV).

**Table 1:** Study characteristics and the accompanying components that were extracted from the selected articles retrieved from the systematic search

Type of study characteristic	Components
Study design	Housing; time of testing; day/night schedule
Animal model/human characteristics	Species; strain/race; age at time of testing/body weight at time of testing; sex; disease induction
Intervention	Type of SSRI; doses; route; injection test interval*; duration of treatment (acute (1 × in 24 h)/subchronic (< 7 days)/chronic treatment (> 7 days)); for subchronic and chronic treatment the number of days were specified
Experimental design	Number of subjects per experimental condition (intervention and control); type of test; test duration; reported outcome behavior
Outcome measures	Most effective dose outcome is based on; fear learning process affected by SSRI (acquisition learning, extinction learning or fear expression after acquisition or extinction learning to either cue or context)

^*^ = timing of SSRI administration relative to the measured fear conditioning process

### Risk of bias assessment

Study quality was assessed with SYRCLE’s risk of bias tool (Hooijmans et al. 2014a, b) which uses three scoring categories: high/unclear/low risk of bias. Risk of bias was assessed based on six general categories, these were: selection bias, performance bias, detection bias, attrition bias, reporting bias, and other biases. For the latter category, we determined the presence of conflict of interest within every article. Since it is known that many preclinical studies show inadequate reporting and therefore lead to the scoring of many items of the risk of bias tool as unclear, two additional reporting items related to randomization and blinding were added to the risk of bias tool. Risk of bias was assessed independently by two reviewers (EH and MV), and discrepancies were resolved by discussion or the consultation of a third reviewer (EYB or LG). Risk of bias was scored as “low” if an article took sufficient measures to minimalize or prevent risk of bias. In contrast, articles reporting that they did not take measures to limit risk of bias were scored as “high” (e.g., we did not randomize the allocation). Items of the risk of bias tool were reported as “unclear” in the situation where insufficient information was available to determine the risk of bias.

“Selective outcome reporting” (reporting bias) was assessed using two procedures: (1) checking whether outcome measures mentioned in the method section were also reported on in the result section and (2) examining the following two databases to determine the preregistration of preclinical studies: Animal Study Registry (https://www.animalstudyregistry.org) and (https://preclinicaltrials.eu/database).

### Extraction of outcome data

Two reviewers (EH, MV) extracted the outcome data of the selected articles; extracted data by one investigator (EH) was cross-checked by the second investigator (MV) and vice versa. The outcome data that was extracted included a descriptive part based on the author’s conclusion (significant increase/no effect/significant decrease in fear learning behavior compared to the placebo-controlled group) and a quantitative part (mean, SD, sample size) for both the control and the SSRI groups. Whenever there was a discrepancy in sample size between the “Materials and methods” and ”Results” section, the sample size reported in the latter was chosen. Data that were only reported graphically were extracted by using a digital ruler (Universal Desktop Ruler). If an article reported multiple time points from the same animal, the measurement with the largest effect was extracted. We extracted and reported the doses that were tested for all experiments separately. However, inclusion of drug effects obtained at suboptimal doses could hamper interpretation of the effect size estimate. Also, inclusion of several data points from one experiment would cause dependency in the data set. Since our main question was if SSRIs could have an effect on fear-learning processes, we therefore only included data for the most effective dose that was tested in the meta-analysis. In case the sample size was described as a range, the highest value was used to calculate the corresponding SD. When articles were found to have missing or unclear data, the authors of the article were contacted. If the requested data was not supplied by the authors in question, the article was removed from the systematic review.

In this article, we distinguished and reported the following processes: acquisition learning, fear expression after acquisition learning, extinction learning, and fear expression after extinction learning. All four fear learning processes can be related to either conditioning to cue or conditioning to context. This yielded a total of eight different fear learning processes. It is important to note that the level of fear induced by acquisition and extinction learning can be measured in two ways: directly during training or in an expression test following the training session. If the level of fear induced by fear learning was only measured in an expression test (and not during training), and the SSRI was administered before and/or during training (and not within 24 h before the expression test), the experiment was categorized as “fear learning measured as expression.”

If a measure of fear learning was only obtained during a fear expression test, and the SSRI was only administered before and/or during training and no longer than 24 h before the fear expression test, the article was allocated to the category “fear learning measured as expression.”

During the extraction of the data, this distinction was made; however, for the meta-analysis, the data of the two methods for measuring acquisition or extinction learning was combined.

### Data-analysis

Meta-analysis was conducted in R (R Core Team 2021). We used the R-package metafor (Viechtbauer 2010) to estimate the overall effect size, using a random-effects model which takes into account the precision of individual studies and the variation between studies and weighs each study accordingly (Hedges and Vevea 1998). Separate meta-analyses were conducted for each of the fear learning processes that were reported in ten or more independent comparisons from at least five different references. In case of multiple use of the same control group, the number of animals used in that control group was divided by the number of times that control group was used in the meta-analysis.

To account for between-studies heterogeneity, five categorical predefined moderators were coded: (SSRI, duration of treatment, disease induction, species, type of test). To encode all conditions of these categorical moderators, 33 dummy variables were created. As the number of moderators was approximately equal to the number of available effect sizes (per process), classical meta-regression models were not identified. This problem can be overcome using Bayesian regularized meta-regression (BRMA), as implemented in the pema package (Van Lissa and van Erp 2021) which applies a horseshoe prior to shrink small regression coefficients towards zero. This aids empirical model identification and helps identify relevant moderator variables (Van Lissa and van Erp 2021). The resulting regression coefficients are negatively biased by design, but the estimate of residual heterogeneity τ^2^ is unbiased. Note that, as this is a Bayesian model, inference is based on credible intervals. A credible interval is interpreted as follows: the population value falls within this interval with 95% probability (certainty). This is different from the interpretation of frequentist confidence intervals, which are interpreted as follows: in the long run, 95% of confidence intervals contain the population value.

Note that some dummy variables in our data set were redundant; this occurs when studies within one sample have identical values on multiple dummy variables. To account for this redundancy, one of these dummy variables was retained, and its name was updated to reflect those of the other redundant dummies it represents. For example, in the “acquisition learning to context” sample, all human studies used the fear potentiated startle test (FPS); no other studies used this test, and no other test was used with humans. Therefore, the dummy for human participants and the one for were identical and their effects could not be distinguished. Thus, the analysis shows their joint effect as an effect of *specieshuman;testFPS*.

To examine the effect of a categorical variable, a reference category must be chosen. Dummy variables encode the difference between each remaining category and this reference category. When examining the results, the intercept represents the expected effect size for a study that falls within the reference category for all categorical variables. The effect of dummy variables represents the difference of that category with the reference category. If a dummy variable has a significant effect, that means that that group’s mean differs significantly from the reference category’s mean (i.e., from the intercept). For the analyses, we used the following reference categories: fluoxetine (SSRI), chronic administration (duration of administration), stressed animals (disease induction), rats (species), and conditioned freezing (type of test). The choice of these categories was based on the expectation that this combination would yield the largest anxiolytic effect of SSRIs on the investigated fear learning processes.

Sensitivity analyses were not prespecified but determined during the course of the systematic review. We performed six sensitivity analyses related to the following experimental characteristics: (1) fear was instructed rather than learned; (2) BrdU was injected to measure cell proliferation; (3) subjects received anesthetics and/or analgesics at some point during the experiment; (4) SSRI was given during both the measured fear learning process and one or more previous process(es) of fear learning (during acquisition and extinction); (5) subjects were exposed to repeated stress caused by daily injections for 5 months; and (6) acquisition or extinction learning to cue or context was measured as fear expression. Sensitivity analyses were carried out one by one, separately for each fear learning process. For each of the six experimental characteristics, experiments were excluded from the data set to determine whether the results obtained in the meta-analyses were robust to the decisions made regarding the six characteristics mentioned above.

Publication bias was examined using visual inspection of funnel plots, Eggers’ regression test, and Trim and Fill analysis. A minimum of 15 studies (for each outcome) was required to analyze publication bias. Funnel plots were made by plotting the SMD against 1/√n. We used this n-based precision estimate for standardized mean differences to avoid distortion of the funnel plots (Zwetsloot et al. 2017).

Asymmetry of the plots, as determined by visual inspection, was used as an indication of publication bias. Egger’s regression test focusses on small study effects and is a linear regression of the effect sizes on their standard errors, weighted by their inverse variance. In the absence of publication bias, the regression’s slope is expected to be zero (Lin and Chu 2018). The Trim and Fill analysis not only aims to detect funnel plot asymmetry by removing small, imprecise studies, but may also provide an estimate of the number of missing studies in the funnel plot and adjusts the overall effect size accordingly, thereby giving an effect estimation of the publication bias. Both the Egger’s regression test and the Trim and Fill analysis were performed in the program Stata.

## Results

### Adjustments to the protocol

For this systematic review, we performed an analysis that was not originally planned in the preregistered protocol. Instead of a subgroup analysis we performed a Bayesian regularized meta-regression given the small sample size and the large number of moderators.

### Data documentation

The Workflow for Open Reproducible Code in Science (WORCS) was used to make analyses reproducible (Van Lissa et al. 2021). All analysis code, supplemental materials, and synthetic data are available as a reproducible repository on GitHub at 10.17605/OSF.IO/MQHJR. The original data (restricted) and all study documentation are available via 10.1007/s00213-023-06333-7.

### Study selection

**Fig. 1:**
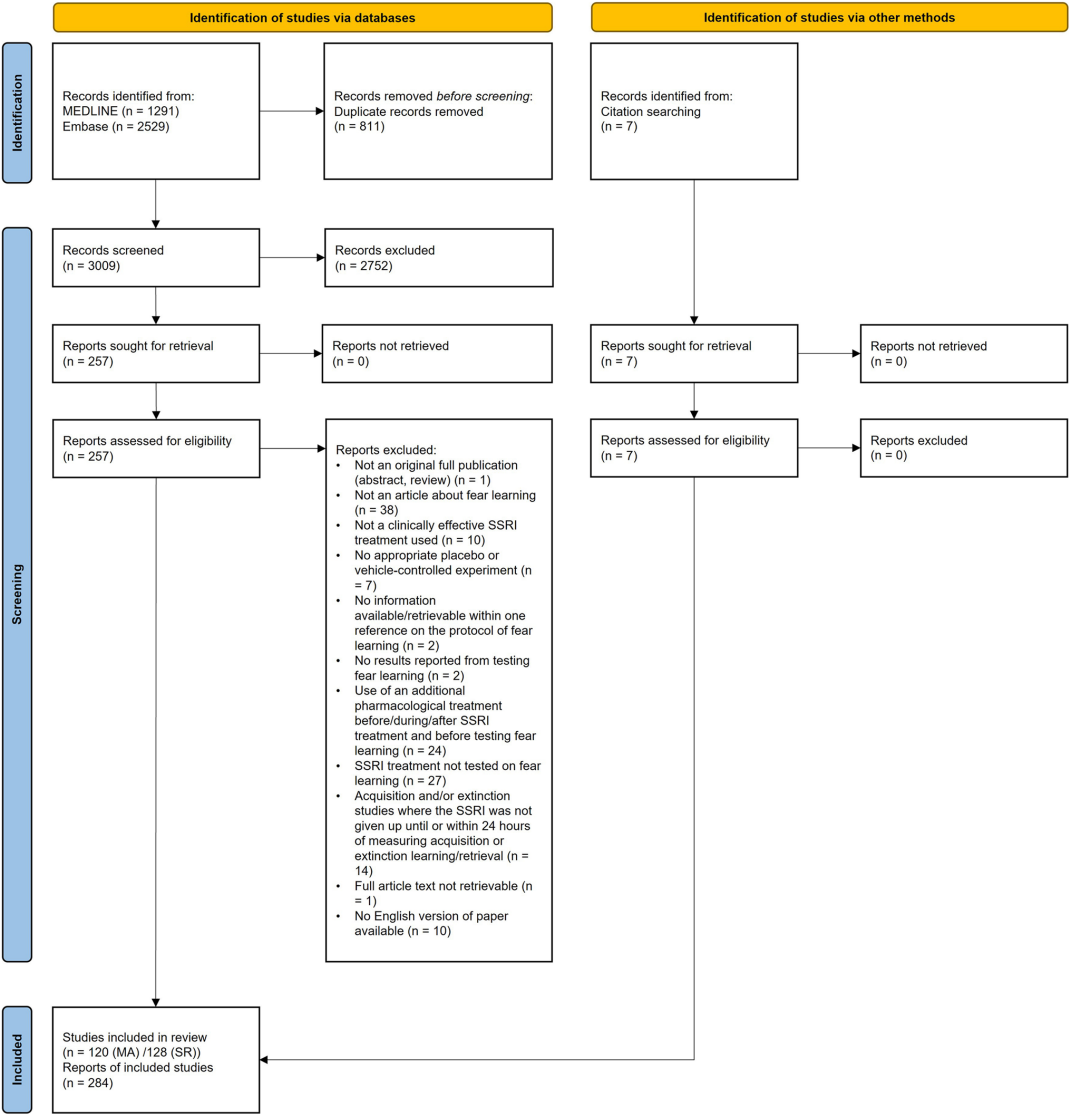
Flowchart of the study selection process

The systematic search for relevant literature in the Medline and Embase databases yielded 3009 unique articles. Screening of the titles and abstracts of these individual articles revealed that 257 articles were eligible for full text screening, seven additional articles were identified through citation screening. After full-text screening, 128 articles were included in the systematic review of which 120 articles were eligible for the meta-analysis. The whole selection procedure is visualized in a flowchart (Fig. 1).

### Study characteristics

For this article, we distinguished eight different fear learning processes. All eight processes were reported on in at least one of the 128 articles included in this systematic review. Acquisition learning was studied in 50 articles (16 to cue (6 of which as expression), 16 to context (12 of which as expression)). Fear expression after acquisition learning was studied in 88 articles (23 to cue, 65 to context). Extinction learning was studied in 15 articles (9 to cue (2 of which as expression), 4 to context), and 11 articles studied fear expression after extinction learning (6 to cue, 5 to context). A complete overview of study characteristics of the included studies can be found in supplementary file S3.

### Risk of bias assessment

**Fig. 2:**
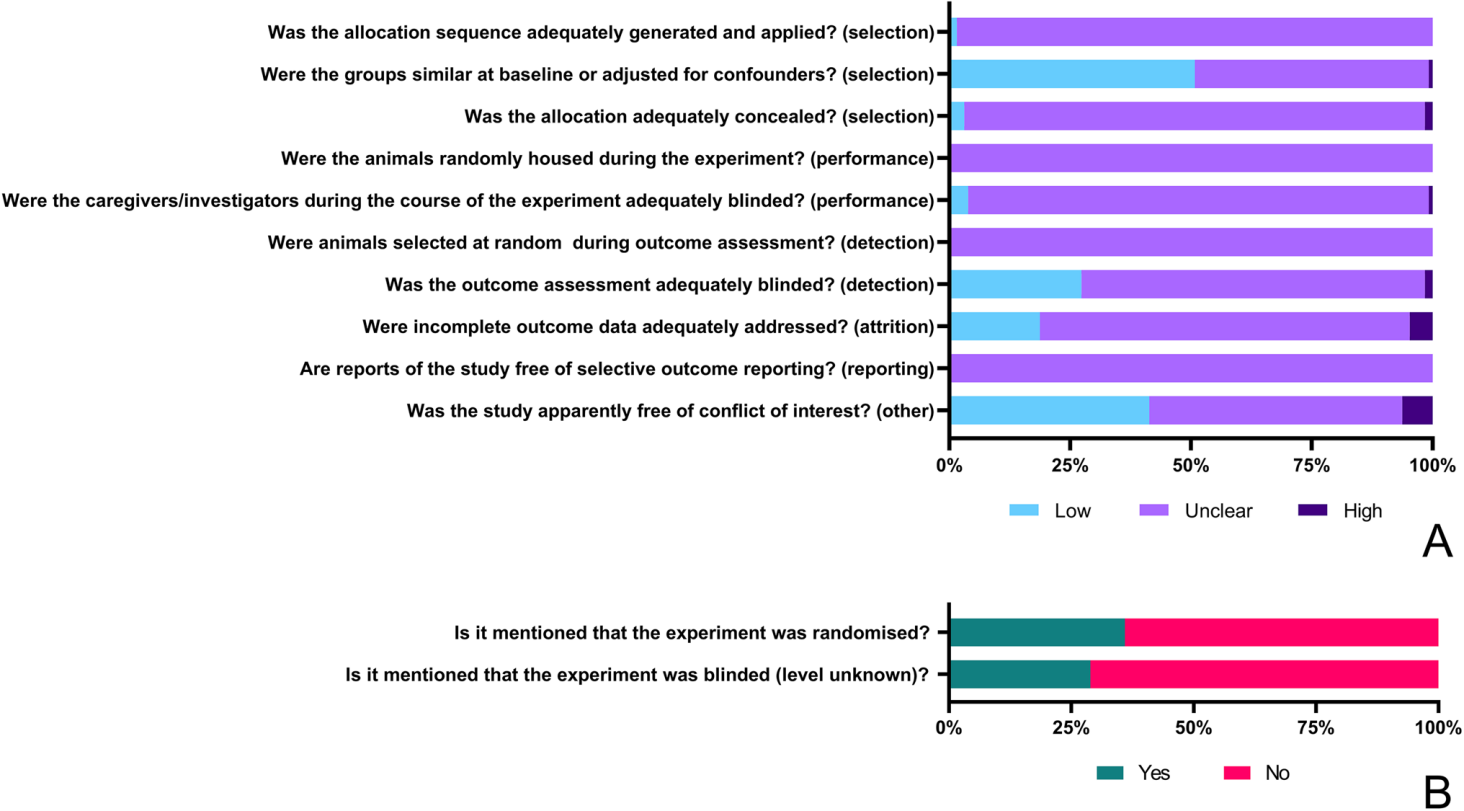
Risk of bias assessment of the included studies. The first nine questions shown in this figure were answered with “yes” indicating low risk of bias, “unclear” if not reported, and “no” indicating high risk of bias (**A**). The last two questions were answered with “yes” if there was any mention and “no” if there was no mention (**B**)

Risk of bias of the individual studies was assessed using the SYRCLE Risk of bias tool for animal studies (Hooijmans et al. 2014a, b). The complete risk of bias assessment can be found in supplementary file S3. Reporting of the six risk of bias categories was poor in almost all included articles. This lead to a predominantly unclear risk of bias score (Fig. 2). Regarding selection bias, 98% of the articles did not report on an adequately generated and applied allocation sequence. In 95% of the articles, the allocation concealment was not properly described or not described at all. On the other hand, 51% of the articles used experimental groups that were similar at baseline or were adjusted for confounders. Assessment of performance bias showed that none of the articles described whether the animals were randomly housed during the experiment. Also, 95% of publications did not describe whether caregivers/investigators were adequately blinded during the experiment. Evaluation of detection bias revealed that none of the articles reported whether the animals were selected at random during outcome assessment. In addition, the blinding of the outcome assessment was not specified in 71% of the articles. The risk of attrition bias was unclear in 76% of articles, since it was not described whether incomplete outcome data were adequately addressed. Likewise, the risk of reporting bias was unclear in all articles since none of the included studies was preregistered and therefore selective outcome reporting was assessed as unclear. Lastly, 52% of the publications did not report whether the study was free of conflict of interest, which could cause a high risk of bias (Fig. 2a).

Two additional reporting items were scored, which revealed that 36% of publications mentioned that the experiment was randomized at any level and 29% of articles described that the experiment was blinded at any level (Fig. 2b).

### Results per fear learning process

**Fig. 3:**
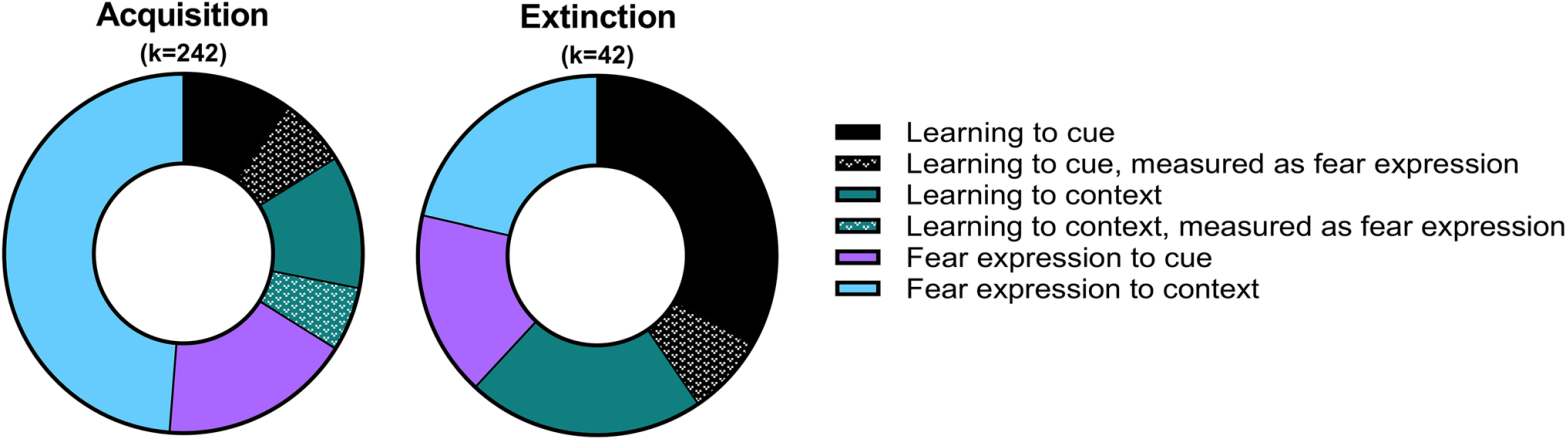
Visual representation of the number of experiments per fear learning process for both acquisition, *k* = 242 experiments (left) and extinction, *k* = 42 experiments (right) included in this systematic review

The 128 included articles reported a total of 284 unique experiments (*k* = 284). Acquisition learning was assessed in 82 experiments (24 to cue (15 of which as expression), 29 to context (14 of which as expression)) and 160 experiments assessed fear expression after acquisition learning (42 to cue, 118 to context). Extinction learning was assessed in 23 experiments (14 to cue (3 of which as expression), 9 to context) and 16 experiments assessed fear expression after extinction learning (7 to cue, 9 to context) (Fig. 3).

Below, the effects of SSRIs on fear learning are discussed separately for each fear learning process. For each process the descriptive statistics and, if performed, meta-analysis is described. If there was insufficient data to perform a meta-analysis for a particular fear learning process, the individual data are reported in a forest plot which can be found in the supplementary files. Results from the meta-analysis with subgroup analysis using the DerSimonian and Laird method (as planned in the original protocol) can be found in supplementary file S4. Results of both the classical meta-regression and the BRMA can be found in supplementary file S5.

### Acquisition learning to cue

#### Descriptive statistics

The 20 articles that studied acquisition learning to cue reported a total of 39 unique experiments (*k* = 39). Out of these 20 articles, the SSRI most frequently investigated was fluoxetine (*n* = 8; rats, 5–20 mg/kg; common goldfish, 81 ug/L) followed by citalopram (*n* = 4; rats, 10 mg/kg; humans, 10–20 mg), escitalopram (*n* = 3; rats, 5 mg/kg; mice, 10 mg/kg; humans, 10 mg/day), paroxetine (*n* = 3; rats, 5–10 mg/kg; mice, 5.5 mg/kg) and sertraline (*n* = 2; rats, 10 mg/kg; mice, 15 mg/kg). Most articles studied chronic administration of the SSRI (*n* = 13), 9 investigated acute administration and 1 article administered the SSRI subchronically. Rats were studied in 12 articles, mice in 4 articles, humans in 3 articles, and 1 article investigated the common goldfish. The majority of articles studied healthy, wild-type, naive or non-stressed subjects (*n* = 17), 5 articles used models of stress (single prolonged stress (*n* = 3), restraint (*n* = 1), acute uncontrollable stress (*n* = 1), immobilization stress (*n* = 1), and chronic social defeat (*n* = 1)) and 1 article used the genetic mouse model VGV 5-HT2CR. Fear learning was assessed with conditioned freezing (*n* = 14), active avoidance (*n* = 4), or fear potentiated startle (*n* = 2). Most articles used male study subjects (*n* = 15), two articles used female study subjects, two articles used both sexes as study subjects, and two articles did not report the sex of their study subjects.

#### Meta-analysis

**Fig. 4:**
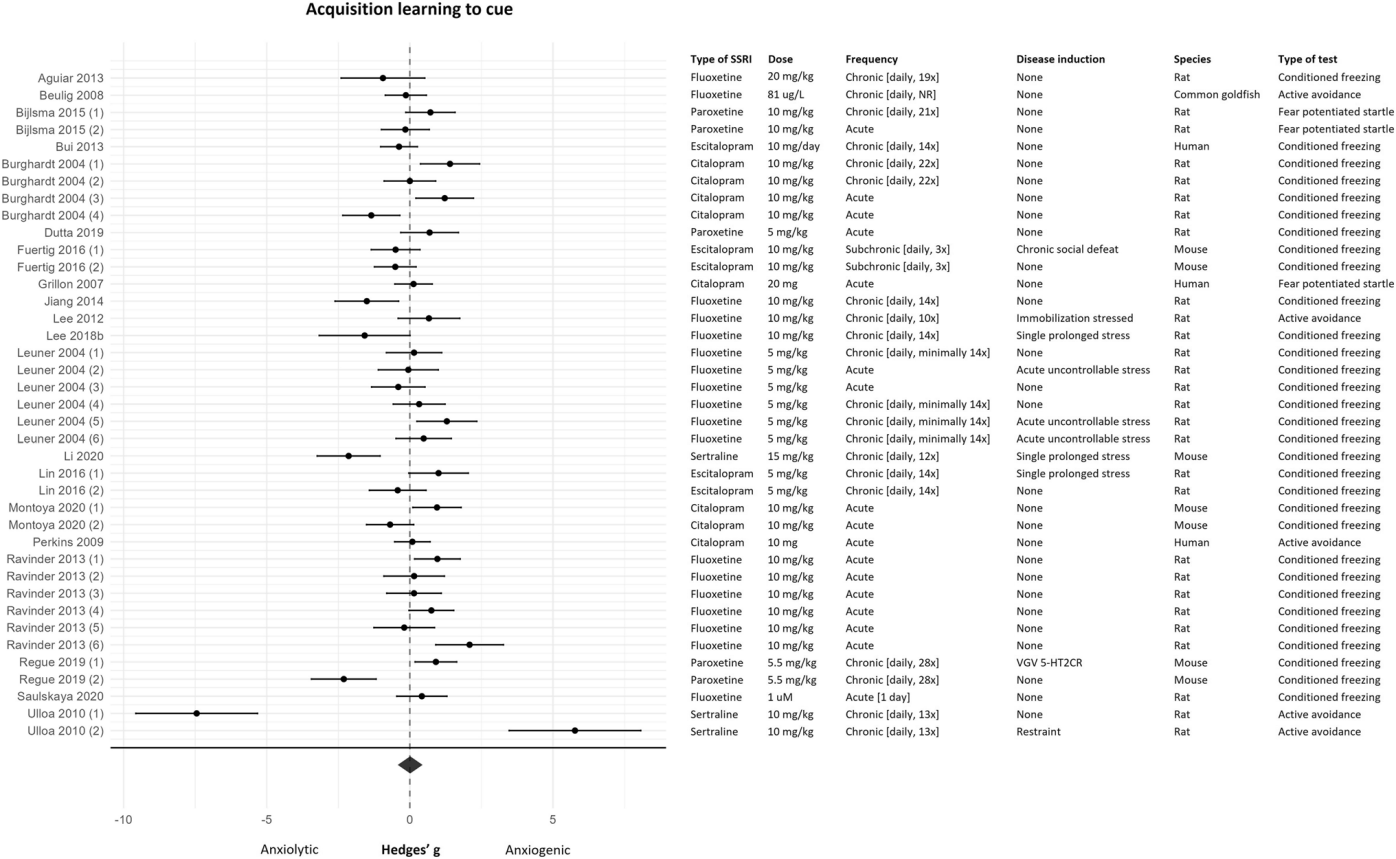
Forest plot of acquisition learning to cue with corresponding study characteristics per experiment. The overall effect was non-significant (SMD (95% CI) 0.01 (− 0.42, 0.44)). VGV 5-HT2CR = valine-glycine-valine isoform of the serotonin 2C receptor, NR = not reported

For the meta-analysis, data of 39 experiments (*k* = 39) was available. Of these 39 experiments, acquisition learning to cue was measured in 24 experiments, and in 15 experiments acquisition learning was measured as fear expression to cue. No effect of SSRIs on acquisition learning to cue was observed (effect size (95% CI) − 0.07 (− 0.45, 0.31); τ^2^ 1.17 (0.89, 3.25); *I*^2^ 84%; *k* = 39). However, this overall effect should be interpreted with caution since the investigated data showed high levels of residual heterogeneity that could not be explained by the moderator analysis. The forest plot in Fig. 4 showed that the results of this fear learning process are contradictory, 5 experiments reported an anxiolytic effect of SSRIs, 8 experiments reported an anxiogenic effect, and 26 experiments reported no effect of SSRIs on acquisition learning to cue.

### Acquisition learning to context

#### Descriptive statistics

The 25 articles that investigated acquisition learning to context reported 43 unique experiments in total (*k* = 43). Of these 25 articles the majority tested fluoxetine (*n* = 14; rats, 5–30 mg/kg; mice, 10–20 mg/kg), followed by citalopram (n = 7; rats, 10–20 mg/kg; mice, 10 mg/kg; humans, 20 mg), escitalopram (*n* = 3; rats, 1–10 mg/kg; mice, 10 mg/kg), fluvoxamine (*n* = 1; rats, 10 mg/kg), paroxetine (*n* = 2; rats, 0.15–20 mg/kg), and sertraline (*n* = 3; rats, 20 mg/kg; mice, 15 mg/kg). Acute SSRI administration was performed in 12 articles, subchronic administration in 3 articles, and chronic administration in 10 articles. The studied subjects were rats (*n* = 17), mice (*n* = 7), and humans (*n* = 1). The majority of the articles studied healthy, wild-type, naive, or non-stressed subjects (*n* = 21), 4 articles used the single prolonged stress model, 1 article the chronic social defeat model, 1 article the maternal separation model, 1 article the olfactory bulbectomy model, and 1 article used the genetically modified mouse model Zfpm1CKO. Fear learning was assessed by conditioned freezing (*n* = 15), active avoidance (*n* = 4), passive avoidance (*n* = 5), or fear potentiated startle (*n* = 1). Most articles used male study subjects (*n* = 21), three articles used female study subjects, and one article used both sexes as study subjects.

#### Meta-analysis

**Fig. 5:**
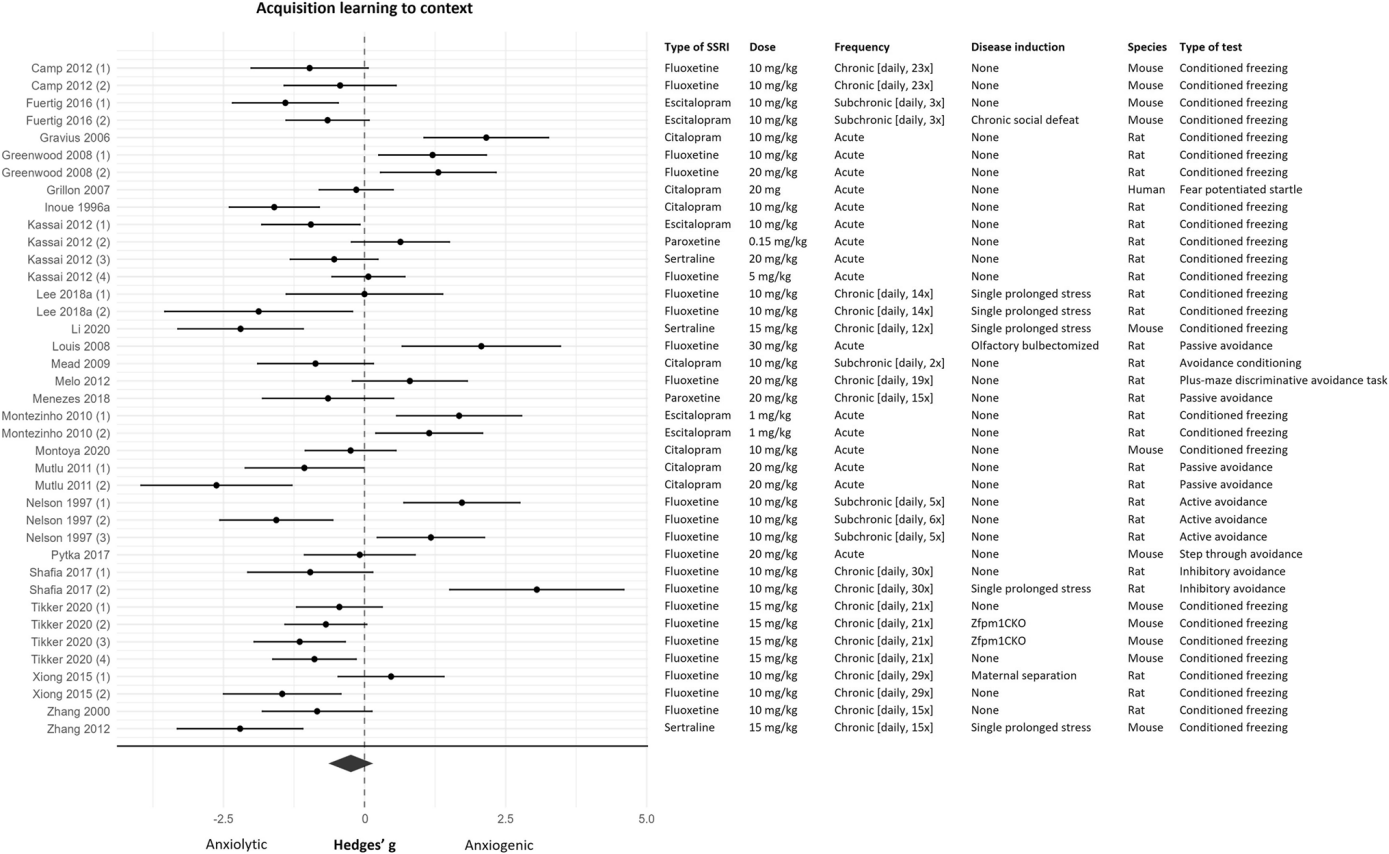
Forest plot of acquisition learning to context with corresponding study characteristics per experiment. The overall effect was negative (favoring SSRIs) and non-significant (SMD (95% CI) − 0.24 (− 0.64, 0.15)). Zfpm1CKO = Zfpm1 conditional knockout

Data of 39 experiments (*k* = 39) was available for the meta-analysis. Two articles comprising 4 experiments were not included in the meta-analysis since information on the dispersion of the data was missing (Archer 1982; Archer et al. 1984). Of the 39 experiments, acquisition learning to context was measured in 25 experiments and acquisition learning measured as fear expression to context in 14 experiments. No effect of SSRIs on acquisition learning to context was observed (effect size (95% CI) − 0.24 (− 0.64, 0.15); τ^2^ 1.32 (0.83, 2.59); *I*^2^ 85%; *k* = 39). Given the high levels of heterogeneity, however, the overall effect should be interpreted with caution. The forest plot in Fig. 5 showed that the results of this fear learning process were ambiguous, 11 experiments reported an anxiolytic effect of SSRIs, 9 experiments reported an anxiogenic effect, and 19 experiments reported no effect of SSRIs on acquisition learning to context.

### Cued fear expression after acquisition learning

#### Descriptive statistics

The 23 articles that studied cued fear expression after acquisition learning reported 42 unique experiments in total (*k* = 42). The majority of articles tested the SSRI fluoxetine (*n* = 12; rats, 10 mg/kg; mice, 10–20 mg/kg; rabbits, 3 mg/kg) followed by paroxetine (*n* = 7; rats, 1–20 mg/kg; mice, 3–10 mg/kg), citalopram (*n* = 3; rats, 10 mg/kg; mice, 10 mg/kg; humans, 10 and 20 mg), fluvoxamine (*n* = 2; rats, 5–20 mg/kg; mice, 30 mg/kg), escitalopram (*n* = 1; mice, 10 mg/kg), and sertraline (*n* = 1; rats, 100 mg/kg). These SSRIs were administered acutely in 10 articles, subchronically in 2 articles and chronically in 15 articles. The studied subjects were rats (*n* = 10), mice (*n* = 11), rabbits (*n* = 1), and humans (*n* = 1). Most articles studied healthy, wild-type, naive, or non-stressed subjects (*n* = 19), some articles used models of stress (single prolonged stress (*n* = 3), water-immersion and restraint stress (*n* = 1), or chronic social defeat (n = 1), other articles used genetically modified animals (Pdcd2-/- (*n* = 1) or MeCP2-308 (*n* = 1)), and 1 article looked at two phenotypes in rats characterized as low fear and high fear. Fear learning was assessed by conditioned freezing (*n* = 17), fear-potentiated startle (*n* = 5), or conditioned emotional response (*n* = 1). The majority of articles used male study subjects (*n* = 21), two articles used both sexes as study subjects, and one article did not report the sex of the study subjects.

#### Meta-analysis

**Fig. 6:**
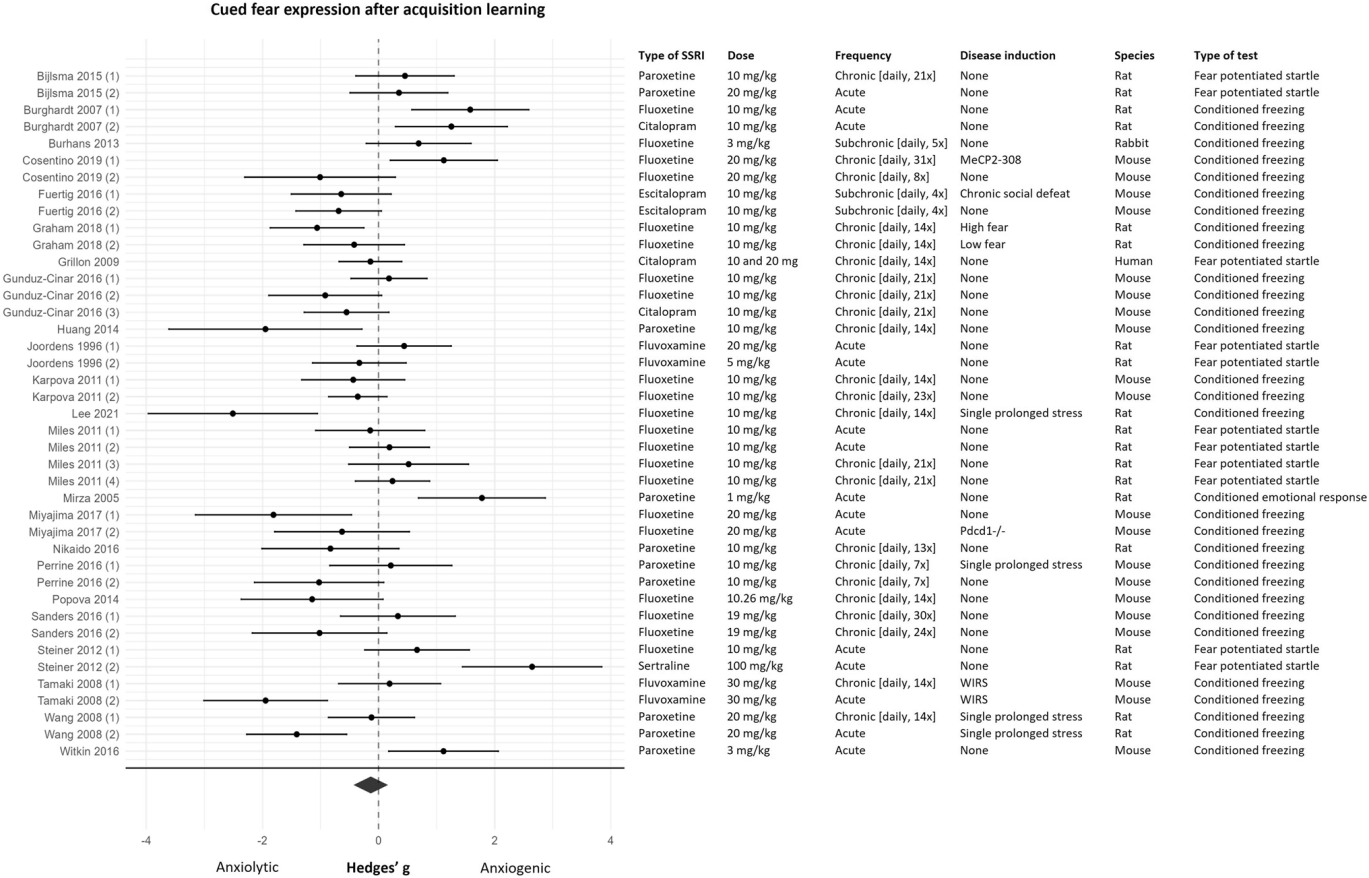
Forest plot of cued fear expression after acquisition learning with corresponding study characteristics per experiment. The overall effect was negative (favoring SSRIs) and non-significant (SMD (95% CI) − 0.13 (− 0.43, 0.16)). WIRS = water immersion restraint stress, MeCP2-308 = truncated form of methyl-CpG-binding protein 2, Pdcd1-/- = PD-1 deficient

Extracted data of 41 experiments (*k* = 41) was included in the meta-analysis. One article, which reported 1 experiment, was not included in the meta-analysis since the quality of figures was too low to reliably extract the data (Hellewell et al. 1999). The forest plot in Fig. 6 shows that the results of this fear learning process were ambiguous, 6 experiments reported an anxiolytic effect of SSRIs, 6 experiments reported an anxiogenic effect, and 29 experiments reported no effect of SSRIs on cued fear expression. No effect of SSRIs on cued fear expression after acquisition learning was observed (effect size (95% CI) − 0.13 (− 0.43, 0.16); τ^2^ 0.71 (0.44, 1.53); *I*^2^ 78%; *k* = 41). However, given the high levels of heterogeneity, this overall effect should be interpreted with caution. The intercept of the BRMA model for this fear learning process was significant (SMD (95% CI) − 0.94 (− 1.65, − 0.21)), indicating that SSRIs significantly reduced cued fear expression whenever studies fell within all of the chosen reference categories (fluoxetine, chronic administration, stressed animals, rats, and conditioned freezing). BRMA further showed that this effect of SSRIs was moderated by duration of treatment. Chronic administration of SSRIs had a significantly larger anxiolytic effect on cued fear expression than acute administration (SMD (95% CI) 0.73 (0.14, 1.26).

### Contextual fear expression after acquisition learning

#### Descriptive statistics

The 65 articles that investigated contextual fear expression after acquisition learning reported a total of 117 unique experiments (*k* = 117). These articles studied the effect of citalopram (*n* = 18; rats, 3–100 mg/kg; humans, 10 and 20 mg; zebrafish, 100 mg/kg), fluoxetine (*n* = 18; rats, 3–60 mg/kg; mice, 2.5–20 mg/kg), sertraline (*n* = 16; rats, 10–20 mg/kg; mice, 15–40 mg/kg), fluvoxamine (*n* = 11; rats, 3–100 mg/kg; mice, 10 – 40 mg/kg), paroxetine (*n* = 8; rats, 2.5–20 mg/kg; mice, 5–10 mg/kg), and escitalopram (*n* = 5; rats, 1–10 mg/kg; mice, 10 mg/kg). Acute administration was performed in 34 articles, subchronic administration in 8 articles, and chronic administration in 29 articles. Most articles investigated rats (*n* = 39) followed by mice (*n* = 24), zebrafish (*n* = 2), and humans (*n* = 1). Nearly all articles studied healthy, wild-type, naive, or non-stressed subjects (*n* = 57), 13 articles used models of stress (single prolonged stress (*n* = 4), time-dependent sensitization (*n* = 4), variable stress (*n* = 3), chronic unpredictable stress (*n* = 1), and chronic social defeat (*n* = 1)), and 3 articles made use of genetically modified animals (3xTgad (*n* = 1), MeCP2-308 (*n* = 1), and Pdcd2-/- (*n* = 1)). Outcome measures by which fear learning was assessed were conditioned freezing (*n* = 55), fear-potentiated startle (*n* = 3), passive avoidance (*n* = 3), conditioned place aversion (*n* = 2), active avoidance (*n* = 1), and conditioned emotional response (*n* = 1). Most of the articles used male study subjects (*n* = 60), two articles used female study subjects, two articles used both sexes as study subjects, and three articles did not report the sex of the study subjects.

#### Meta-analysis

**Fig. 7:**
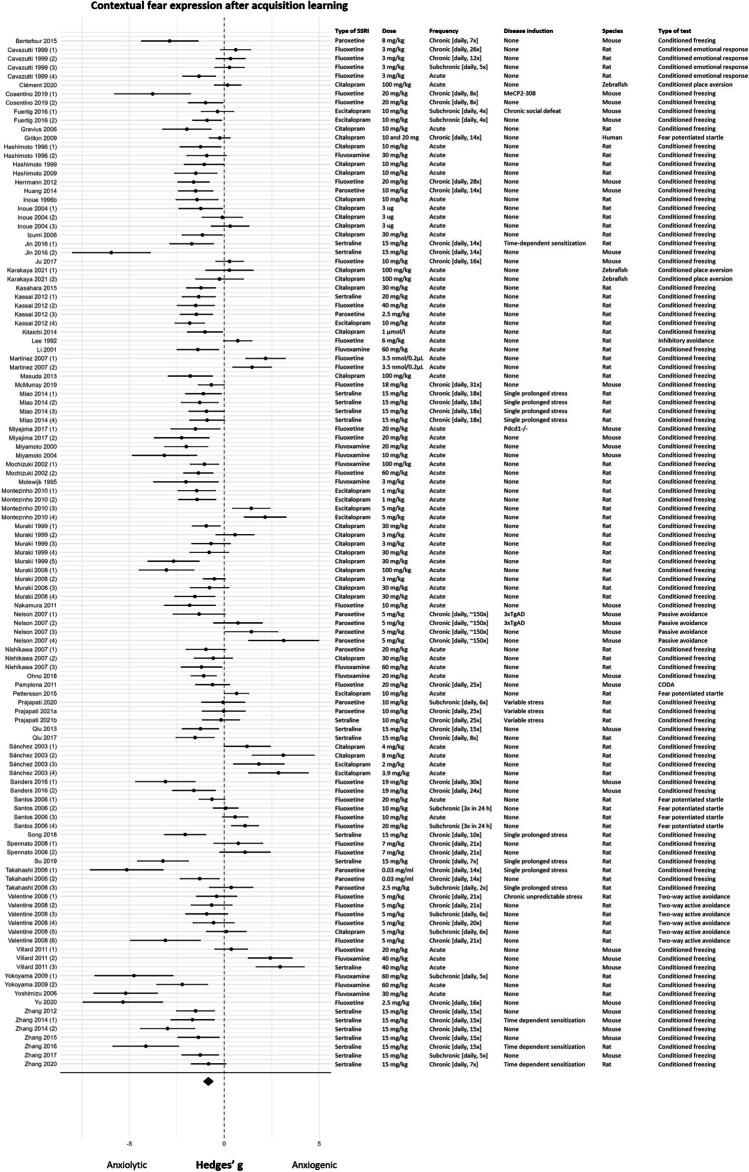
Forest plot of contextual fear expression after acquisition learning with corresponding study characteristics per experiment. The overall effect was negative (favoring SSRIs) and significant (SMD (95% CI) − 0.84 (− 1.11, − 0.56) (*P* < 0.001)). MeCP2-308 = truncated form of methyl-CpG-binding protein 2, Pdcd1-/- = PD-1 deficient, 3xTgAD = triple transgenic Alzheimer’s disease, CODA = conditioned odor avoidance task

The meta-analysis included data extracted from a total of 117 experiments (*k* = 117). One article had an effect size estimate almost 15 standard deviations from the mean effect size (Z = 14.98); this article was removed as an outlier (Verma et al. 2016). The forest plot in Fig. 7 shows that the results of this fear learning process were rather ambiguous, 60 experiments reported an anxiolytic effect of SSRIs, 11 experiments reported an anxiogenic effect, and 46 experiments reported no effect of SSRIs on acquisition learning to context. SSRIs significantly reduced contextual fear expression (effect size (95% CI) − 0.85 (− 1.12, − 0.57); *P* < 0.001; τ^2^ 2.00 (2.04, 4.38); *I*^2^ 89%; *k* = 117). However, caution should be taken when interpreting this overall effect given the presence of high levels of heterogeneity. The intercept of the BRMA model for this fear learning process was significant (SMD (95% CI) − 1.49 (− 2.31, − 0.72)), indicating that SSRIs significantly reduced contextual fear expression whenever studies fell within all of the chosen reference categories (fluoxetine, chronic administration, stressed animals, rats, and conditioned freezing).

### Extinction learning to cue

#### Descriptive statistics

The 9 articles that studied extinction learning to cue reported a total of 17 unique experiments (*k* = 17). The articles investigated paroxetine (*n* = 3; rats, 5–10 mg/kg; mice, 5.5–16 mg/kg), citalopram (*n* = 2; rats, 10 mg/kg; mice, 5–20 mg/kg), fluoxetine (*n* = 2; rats, 7 mg/kg; mice, 18 mg/kg), escitalopram (*n* = 1; humans, 10 mg/day), and sertraline (*n* = 1; rats, 10 mg/kg). These SSRIs were mostly administered chronically (*n* = 6), 2 articles used subchronic administration, and 2 articles gave the SSRIs acutely. The species investigated in the 9 articles were rats (*n* = 5), mice (*n* = 3), and humans (*n* = 1). Most of these articles investigated healthy, wild-type, naive, or non-stressed subjects (*n* = 8), 1 article used single prolonged stress as a model of stress, and 1 article used the genetic mouse model VGV 5-HT2CR. Conditioned freezing was used to test fear learning in 8 articles; 1 article used active avoidance. Most articles used male study subjects (*n* = 7), one articles used both sexes as study subjects, and one article did not report the sex of the study subjects.

#### Meta-analysis

**Fig. 8:**
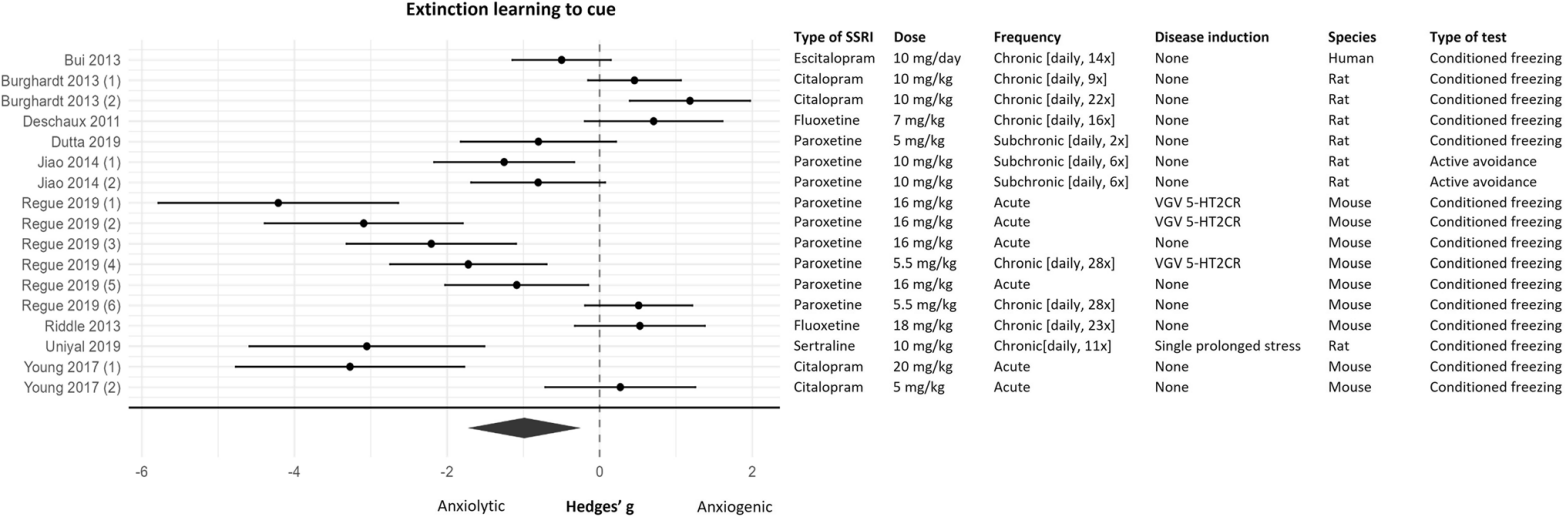
Forest plot of extinction learning to cue with corresponding study characteristics per experiment. The overall effect was negative (favoring SSRIs) and significant (SMD (95% CI) − 0.99 − 1.73, − 0.25) (*P* = 0.01)). VGV 5-HT2CR = valine-glycine-valine isoform of the serotonin 2C receptor

For the meta-analysis, the data of 17 experiments (*k* = 17) was included. Of these 17 experiments, extinction learning to cue was measured in 14 experiments and extinction learning was measured as fear expression to cue in 3 experiments. SSRIs were observed to have a significant anxiolytic effect on extinction learning to cue (effect size (95% CI) − 0.99 (− 1.73, − 0.25); *P* = 0.01; τ^2^ 2.15 (1.09, 5.74); *I*^2^ 91%; *k* = 17). However, this overall effect should be interpreted with caution since the investigated data showed high levels of heterogeneity which could not be explained by the moderator analysis. The forest plot in Fig. 8 shows that the results of this fear learning process were ambiguous, 8 experiments reported an anxiolytic effect of SSRIs, 1 experiment reported an anxiogenic effect, and 8 experiments reported no effect of SSRIs on acquisition learning to context.

### Extinction learning to context

#### Descriptive statistics

The 4 articles that investigated extinction learning to context reported on 9 unique experiments in total (*k* = 9). All 4 articles administered fluoxetine chronically, 1 of the 4 articles also studied the effect of acutely administered fluoxetine (rats, 10–20 mg/kg; mice, 10 mg/kg). Both rats (*n* = 3) and mice (*n* = 1) were used in these articles. These rodents were healthy, wild-type, naive, or non-stressed in all 4 articles; also 1 article used the maternal separation model of stress. The fear learning was assessed with both conditioned freezing (*n* = 3) and active avoidance (*n* = 1). Two articles used male study subjects, one article used female study subjects, and one article used both sexes as study subjects.

This fear learning process was investigated in 9 independent experiments; this number was too small to perform a meta-analysis on (as defined in the preregistered protocol). Details of the experiments for this fear learning process can be found in the forest plot shown in supplementary file S6.

### Cued fear expression after extinction learning

#### Descriptive statistics

The 6 articles that studied cued fear expression after extinction learning reported a total of 7 unique experiments (*k* = 7). Most of these articles studied the effect of fluoxetine (*n* = 4; rats, 10 mg/kg; mice, 10–18 mg/kg), escitalopram (*n* = 1; humans, 10 mg/day), and paroxetine (*n* = 1; rats, 5 mg/kg) were investigated in the other 2 articles. Acute administration was performed in 1 article, subchronic administration also in 1 article, and chronic administration in 4 articles. The species studied for this fear learning process were mice (*n* = 3), rats (*n* = 2), and humans (*n* = 1). All subjects were healthy, wild-type, naive, or non-stressed (*n* = 6). Fear learning was tested in all 6 articles with conditioned freezing. Four articles used male study subjects, one article used both sexes as study subjects, and one article did not report the sex of study subjects.

This fear learning process was investigated in 7 independent experiments; this number was too small to perform a meta-analysis on (as defined in the preregistered protocol). Details of the experiments for this fear learning process can be found in the forest plot shown in supplementary file S6.

### Contextual fear expression after extinction learning

#### Descriptive statistics

The 5 articles that investigated contextual fear expression after extinction learning reported a total of 9 unique experiments (*k* = 9). All 5 articles studied the effect of chronically administered fluoxetine (rats and mice: 10 mg/kg). In 3 articles, the study subjects were mice, and in 2 articles, the subjects were rats. All 5 articles studied healthy, wild-type, naive, or non-stressed animals; 1 of these 5 articles also used the maternal separation model of stress. Conditioned freezing was used in all articles to assess fear learning (*n* = 5). All articles used male study subjects (*n* = 5).

This fear learning process was investigated in 9 independent experiments; this number was too small to perform a meta-analysis on (as defined in the preregistered protocol). Details of the experiments for this fear learning process can be found in the forest plot shown in supplementary file S6.

### Sensitivity analysis

For each fear learning process, multiple sensitivity analyses were performed to check the robustness of the meta-analysis results. Exclusion of the experiments by the characteristics described in the method section did not influence the substantive conclusions on the overall effect of SSRIs on acquisition learning to cue, acquisition learning to context and cued and contextual fear expression after acquisition learning. However, upon exclusion of the 8 experiments in which SSRIs were administered not only during the fear learning process of interest but also during one or more previous process(es) of fear learning, the overall effect of SSRIs on extinction learning to cue was no longer significant but the direction remained the same. Given that the original number of articles investigated was already low (*n* = 17), this loss of statistical significance is likely due to loss of power rather than lack of robustness.

### Publication bias

Visual inspection of the funnel plots of the five fear learning processes included in the meta-analysis showed variable shapes (supplementary file S7). However, these shapes did not show high levels of asymmetry and therefore do not indicate the presence of publication bias. In addition, Egger’s regression test and a Trim and Fill analysis were performed to assess publication bias. In 4 out of 5 investigated fear learning processes, publication bias was found in at least 1 of the 2 analyses (Table 2). The Trim and Fill analysis imputed 12, 7, and 26 “missing” studies for the fear learning processes acquisition learning to cue, cued fear expression after acquisition learning, and contextual fear expression after acquisition learning, respectively (supplementary file S8). Significant publication bias was found for extinction learning to cue in the Egger’s regression test (*p* = 0.016). No publication bias was found for acquisition learning to context.

**Table 2:** Publication bias assessment of the fear learning processes using Egger’s regression test (p value) and the Trim and Fill analysis (imputed missing studies)

Fear learning process	Egger’s regression test	Trim and Fill analysis
Acquisition learning to cue	0.468	12
Acquisition learning to context	0.466	0
Cued fear expression after acquisition learning	0.448	7
Contextual fear expression after acquisition learning	0.15	26
Extinction learning to cue	0.016	0

## Discussion

### Effect of SSRIs on fear learning processes

This systematic review is the first to review the effects of clinically effective SSRIs on fear learning in both animals and humans. Meta-analyses of the included data suggest that treatment with SSRIs reduces the expression of contextual fear after acquisition learning and facilitate extinction learning to cue. Meta-analyses did not suggest an overall effect of SSRIs on acquisition learning to cue or context and on cued fear expression after acquisition learning. BRMA analysis indicated that duration of treatment may influence the effect of SSRIs on the expression of cued fear after acquisition learning.

Our study showed that SSRI treatment reduced contextual fear expression. Interestingly, exacerbated contextual fear expression is considered an indicator of fear generalization. Generalization is characterized by inappropriate fear responses to a variety of stimuli that share resemblance to the original conditioned stimulus, which can be both a cue and a context. Fear generalization is a hallmark of generalized anxiety disorder (Lissek et al. 2014), panic disorder (Lissek et al. 2010), and post-traumatic stress syndrome (Lis et al. 2020) which are treated most effectively with SSRIs (Baldwin et al. 2011; Chawla et al. 2022; Huang et al. 2020). Our results could thus suggest that the effectiveness of SSRIs is partly based on their ability to reduce fear generalization which is reflected in decreased contextual fear expression.

Although alterations in 5-HT transporter expression have been implicated in acquisition learning deficits (Bijlsma et al. 2015; Heitland et al. 2013), this systematic review of pharmacological studies does not provide evidence for the involvement of the 5-HT transporter in acquisition learning since selective blockade of the 5-HT transporter with SSRIs did not seem to affect this fear learning process. This finding is in line with earlier studies that have suggested that 5-HT transporter functioning is specifically important during early development by tuning the neural systems involved in anxiety (Ansorge et al. 2004; Bijlsma et al. 2015). It is, however, important to realize that the absence of a significant overall effect of SSRIs on acquisition learning does not mean that SSRIs do not have an effect on this process. The lack of an overall effect could be due to the shortage of data and the high level of heterogeneity within the studied fear learning processes.

This systematic review also showed that SSRI treatment facilitated extinction learning to cue. However, this overall effect should not be overinterpreted since only a few studies were included and the analysis showed high levels of heterogeneity. Due to the low number of experiments, not all study characteristics within this fear learning process could be investigated properly with the Bayesian regularized meta-regression. For example, only one experiment investigated the effect of SSRIs in stressed animals even though the use of such a disease model could contribute to the translational value of research into treating anxiety-like disorders. Therefore, we want to emphasize that additional research is necessary to confirm the observed effects of SSRI treatment on extinction learning and investigate how this effect is moderated by different study characteristics.

The effects of SSRIs on fear learning processes found in this systematic review can be linked to specific neural mechanisms. The effect of SSRI treatment on contextual fear expression may suggest that the clinical effect of SSRIs is mediated by the BNST. The BNST is involved in processing of sustained and unpredictable threat (Goode et al. 2019). Both PD and PTSD patients show increased BNST activity which is associated with increased sensitivity to unpredictable threat (Brinkmann et al. 2017a, b; Brinkmann et al. 2017a, b). Serotonin is thought to decrease BNST activity (as reviewed in: Burghardt and Bauer (2013)). and, therefore, SSRI treatment may have a normalizing effect on exacerbated BNST-mediated fear responses. Furthermore, GAD, PD, and PTSD have all been associated with decreased fear extinction (Michael et al. 2007; Milad et al. 2008; Otto et al. 2014; Pitman and Orr 1986). As amygdala reactivity has been implicated as an indicator of treatment responsiveness (Gorka et al. 2019) and the amygdala has an important role in extinction learning (Phelps et al. 2004), our findings could also indicate that the clinical effect of SSRIs is mediated by its facilitatory effect on extinction learning.

In addition, our results do not suggest an emotional blunting effect of SSRIs since no effect of SSRIs on acquisition and cued fear expression was found. It could, however, be that the anxiolytic effect of SSRIs on contextual fear expression and extinction learning to cue is set about by a general inhibition of fear-related emotions. Therefore, it would be interesting to investigate how SSRIs affect other forms of anxiety, such as unconditioned fear responses. To our knowledge, the effects of SSRIs on unconditioned anxiety, such as innate anxiety behavior and approach-avoidance behavior, show varying results. Acute administration of SSRIs, for example, has an anxiolytic effect in the separation-induced vocalization test in guinea-pig pups (Groenink et al. 2015). But both acute and chronic administration of SSRIs have anxiogenic-like or no effects in the elevated plus maze (reviewed in: Borsini et al. (2002)). Hence, we are currently performing a systematic review and meta-analysis in which we investigate the effect of SSRIs on unconditioned anxiety (PROSPERO registration number: CRD42022371871).

Bayesian regularized meta-regression suggested that the effect of SSRIs on cued fear expression may be dependent on duration of the treatment. Chronic SSRI treatment was associated with a stronger reduction in cued fear expression than single SSRI treatment as measured by conditioned freezing of fluoxetine-treated stressed rats. This finding is interesting in light of the clinically observed delayed onset of action of SSRIs in the treatment of anxiety disorders (Lenze et al. 2005; Rickels et al. 2003). This delayed onset of action of SSRIs is often attributed to the time it takes for the 5HT1A receptor to desensitize, and the expression of brain-derived neurotrophic factor to increase (reviewed in: Blier et al. (1987)). Both the 5HT1A receptor and BDNF are involved in fear learning (reviewed in: Dincheva et al. (2016)). In addition, this delayed onset could also be related to downregulation of the NR2B subunit of the NMDA receptor which was observed in the basolateral amygdala of rats after chronic SSRI treatment (Burghardt et al. 2013). The NMDA receptor plays an important role in synaptic plasticity and fear learning (Liu et al. 2004; Zhao et al. 2005). It is interesting that an effect of duration of treatment was only found for cued fear expression and not for contextual fear expression. It is currently unknown what could explain this discrepancy between the effects on these two fear processes. This would be an interesting line of future research.

Given that patients may respond to one SSRI but not to another (Baldwin et al. 2014), one of our sub-questions if the six clinically prescribed SSRIs would differ in the fear learning processes they affect and in the extent to which they would do so. Most SSRIs are considered selective towards the 5-HT transporter (Owens et al. 2001). As reviewed by Sanchez and co-workers, the selectivity of SSRIs for SERT relative to the nearest target ranges from over 60 times for sertraline to over a 1000 times for escitalopram (Sanchez et al. 2014). Fluvoxamine is the only SSRI that binds with considerable affinity towards another target than SERT. It binds with a 15 times weaker affinity to sigma1 receptors, at which it acts as an agonist (Ishikawa et al. 2007). Currently, however, it is unclear to what extent binding to these other proteins are involved in the anxiolytic effects of SSRIs. The extent to which the different SSRIs act as orthosteric or allosteric modulator of the SERT protein may also affect their efficacy (reviewed in: Sanchez et al. (2014)). Our meta-analysis, which only included the most effective dose within each experiment, did not provide evidence that the six SSRIs differ in the way they affect fear learning. This finding may suggest that SSRIs exert their effect on fear learning via inhibition of SERT, which would be in line with the high selectivity of SSRIs towards SERT (Owens et al. 2001; Sanchez et al. 2014). Of note, given the limited data available for some of the SSRIs, the meta-analysis may not have been sufficiently powered to reliably detect differences between SSRIs.

### Heterogeneity

In general, we found high levels of statistical heterogeneity (I^2^ and τ^2^) for all the investigated fear learning processes. This is, however, not surprising since most included experiments were animal studies which often vary considerable in their experimental set-up compared to clinical trials (Hooijmans et al. 2014a, b).

Bayesian regularized meta-regression was performed to investigate the impact of the study characteristics on the overall heterogeneity within the fear learning processes. This analysis suggested that SSRIs reduce cued and contextual fear expression after acquisition learning when the moderators fall within the chosen reference categories (fluoxetine, chronic administration, stressed animals, rats, conditioned freezing). Interestingly, no overall effect of SSRIs on cued fear expression was found in this systematic review. This suggests that a model of purposefully chosen reference categories may resolve part of the heterogeneity and reveal an effect of SSRIs on cued fear expression, suggesting the importance of the experimental set-up of a study.

The BRMA analysis was used since the investigated dataset was quite small, compared to the many moderators that were included. A BRMA analysis tends to overfit data less than a classical meta-regression analysis and was therefore the preferred type of analysis (Van Lissa and van Erp 2021). In addition, the classical meta-regression analysis showed moderate to high levels of multicollinearity present within our dataset as indicated by the variance inflation factor (supplementary file S5) and the BRMA is robust to multicollinearity.

### Limitations

#### Knowledge gaps

First, only a small number of studies have investigated the effect of SSRIs on fear learning processes in humans. Data from these studies would give direct insight into the effect of SSRIs on the different fear learning processes. Second, the articles included in this systematic review did not allow us to investigate the potential differences between the six SSRIs, since too few experiments were conducted. Therefore, we cannot draw any conclusions regarding the efficacy of the different SSRIs. Third, the majority of subjects that were studied were healthy even though an experimental set-up with stressed subjects may hold greater translational value since it bears more resemblance to the clinical situation of patients with anxiety-like disorders. The same holds true for the sex of the studied subjects. In 87% of all experiments, only male subjects were tested and in 6% of the experiments females were studied. In 4% of the experiments, both sexes were tested without differentiation between the sexes and in the remaining experiments the sex of the studied subjects was not reported. Including female subjects in the experimental set-up could increase the translational value of these studies since psychiatric epidemiology shows that women have a significant higher chance of developing anxiety disorders than men (Angst and Dobler-Mikola 1985; Bruce et al. 2005; Regier et al. 1990). Fourth, only a few experiments used either the active or passive avoidance tests and, therefore, not enough data was available to include these tests in the moderator analysis. It would have been interesting to determine the effect of SSRIs on this behavioral aspect of anxiety considering that avoidance behavior is a key aspect of anxiety-like disorders (reviewed in: Hofmann and Hay (2018); Krypotos et al. (2015)). Similarly, only a few articles investigated the effect of SSRIs on fear extinction. It is, however, hypothesized that deficits in fear extinction contribute to the development of anxiety disorders (Milad et al. 2014; Wessa and Flor 2007), and various exposure therapies in patients are based on promoting extinction (Hermans et al. 2006). Therefore, additional preclinical studies specifically investigating effects of SSRIs on fear extinction processes may provide a better understanding of relevant disease processes and could help to improve the treatment of anxiety-like disorders.

#### Limitations of the included studies

The articles included in this systematic review showed some limitations which should be considered for proper interpretation of the data. First, in some articles, the SSRI was administered during multiple processes of fear learning. This makes it difficult to pinpoint the effect of the SSRI on the specific processes. For example, in some studies, the SSRI was administered during both the acquisition and extinction phase. The effect of the SSRI on the extinction phase could therefore have been influenced by the effect that the SSRI already may have had on the acquisition phase. Even though the sensitivity analysis did not indicate that this experimental set-up affected the overall effect, the measured and reported effect of the SSRI on extinction is not specific. Second, the majority of the included articles reported very poorly on preventive measures taken to reduce risk of bias. It is, however, difficult to determine whether the articles did not report the preventive measures or whether they did not apply these. Nonetheless, we did not exclude articles based on their risk of bias assessment. Lastly, publication bias has likely occurred in four out of five investigated fear learning processes as determined by the Egger’s regression test and the Trim and Fill analysis. Taking these limitations into consideration, it is important to interpret the results with caution since the overall effects of SSRIs on fear learning are most probably overestimated.

#### Limitations of the design of the systematic review

This systematic review only included articles written in English or Dutch. This decision was based on a cost–benefit analysis. The excluded articles were either written in Chinese (Ji et al. 2015; Sun et al. 2016; Wang et al. 2014; Wen et al. 2018) or Japanese (Hashimoto 2000; Inoue 1993; Muraki 2001; Tsuchiya 1999). In addition, the chosen approach to include only one datapoint per animal and to include the datapoint of the fear learning process measured most directly after SSRI treatment might partly explain the low number of fear extinction experiments included in this systematic review. It also reveals that only few experiments investigated the effect of SSRIs on fear extinction processes with as little interference as possible. Also, by only including the most effective dose of experiments in the meta-analysis, we may have introduced a bias regarding the pharmacological selectivity of the effect of SSRIs. This seems, however, not likely since in only 27% of all experiments a dose–response relationship was studied, and in 66% of these experiments the most effective dose was also the highest dose tested. Furthermore, not all the biological study characteristics were included as moderators (such as sex, age, dose, route of administration, etc.). On the basis of previous research, a selection of relevant moderators was made since a relatively high number of experiments is needed per parameter to reliably examine heterogeneity (Riley et al. 2011). We cannot exclude that some of the remaining heterogeneity could have been explained by study characteristics not included in the moderator analysis. Future research might seek to better understand the sources of heterogeneity observed in our meta-analyses. This could help optimize and standardize the experimental set-up of future studies on the mechanisms underlying fear learning.

## Conclusion and recommendations

In conclusion, this review provided evidence to suggest that the clinical efficacy of SSRIs may be related to their effects on fear expression and extinction. Given the findings of the current review, it would be important to conduct additional studies that are tailored to specifically measure drug effects on fear extinction, instead of multiple fear learning processes, to further validate and extend the effect of SSRIs on fear extinction. In addition, the effects of SSRIs on these fear processes could also be related to a general inhibition of fear-related emotions. It would therefore be interesting to investigate if SSRIs affect other forms of anxiety, such as unconditioned fear.

We would further recommend to more frequently use human subjects in this line of research. Human models of anxiety, such as anxiety-potentiated startle, have shown great potential as an experimental tool and have high translational value (Grillon and Ernst 2020). Lastly, the conduct of systematic reviews with meta-analyses that include animal studies is still under development. An important limitation when synthesizing evidence of animal studies is that the number of available studies is often small and the between study heterogeneity is most times high. To address this problem, we applied the BRMA analysis as a valuable alternative to subgroup analyses. We would like to encourage fellow researchers to consider using BRMA when working with small datasets containing high levels of multicollinearity to limit overestimation of estimated effects.

### Supplementary Information

Below is the link to the electronic supplementary material.

Supplementary file1 (PDF 287 KB)

Supplementary file2 (PDF 168 KB)

Supplementary file3 (XLSX 143 KB)

Supplementary file4 (PDF 1059 KB)

Supplementary file5 (HTML 8777 KB)

Supplementary file6 (PDF 428 KB)

Supplementary file7 (PDF 168 KB)

Supplementary file8 (PDF 502 KB)
