# Supplementary material for: The effect of SSRIs on fear learning: a systematic review and meta-analysis
Source: Psychopharmacology (Berl). 2023 Feb 27;240(11):2335–59. doi: 10.1007/s00213-023-06333-7 (PMC10593621; doi:10.1007/s00213-023-06333-7)
Supplement: Supplementary file 1 — Supplementary file1 (PDF 287 KB) [file 213_2023_6333_MOESM1_ESM.pdf]

The effect of SSRIs on fear learning: a systematic review and meta-analysis

Psychopharmacology

Elise J Heesbeen, Elisabeth Y Bijlsma, P Monika Verdouw, Caspar van Lissa, Carlijn Hooijmans, Lucianne Groenink

Corresponding author: Lucianne Groenink, l.groenink@uu.nl

Supplementary file S1. SYRCLE protocol

SYSTEMATIC REVIEW PROTOCOL FOR ANIMAL INTERVENTION STUDIES

FORMAT BY SYRCLE (WWW.SYRCLE.NL)

VERSION 2.0 (DECEMBER 2014)

Item #	Section/Subsection/Item	Description	Check for approval
	A. General		
1.	Title of the review	The effect of SSRIs on fear learning: a systematic review (and meta-analysis)	
2.	Authors (names, affiliations, contributions)	E. Heesbeen, Department of Pharmacology, University Utrecht, The Netherlands<br>E.Y. Bijlsma, Department of Pharmacology, University Utrecht, The Netherlands<br>P.M. Verdouw, Department of Pharmacology, University Utrecht, The Netherlands<br>C. R. Hooijmans, Department for health evidence unit SYRCLE and Department of Anesthesiology. Radboud University Medical Centre Nijmegen, The Netherlands<br>L. Groenink, Department of Pharmacology, University Utrecht, The Netherlands	
3.	Other contributors (names, affiliations, contributions)		
4.	Contact person + e-mail address	Elise Heesbeen, e.j.heesbeen@uu.nl	
5.	Funding sources/sponsors	ZonMw, MKMD 114024147	
6.	Conflicts of interest	None to declare	
7.	Date and location of protocol registration	Utrecht, 30/09/2020	
8.	Registration number (if applicable)		
9.	Stage of review at time of registration		
	B. Objectives		
	Background		

10.	What is already known about this disease/model/intervention? Why is it important to do this review?	Fear-learning is a central process underlying the development of anxiety-like disorders, such as panic disorder, generalized anxiety disorder and post-traumatic stress disorder. Disruptions in either the acquisition or extinction of fear may result in excessive fear expression and fear generalization. Given that individuals suffering from anxiety disorders are often unable to inhibit or extinguish fear responses, treatment of anxiety disorders is mostly based on cognitive behavioural therapy (CBT), aimed at enhancing fear inhibition or fear extinction. CBT may be combined with treatment with selective serotonin re-uptake inhibitors (SSRIs) to enhance treatment response. This kind of treatment of anxiety disorders is relatively successful, yet 40% of patients remains unresponsive to treatment or suffers from residual symptoms. This means there is ample room for improvement.<br><br>Experimental animal studies focusing on fear learning are frequently used to find new ways to enhance treatment effects of CBT. For this various tests, procedures and species are used. Studies may address either acquisition or extinction, use acute or chronic dosing and test in animals following disease induction or in healthy animals.<br><br>With this systematic review we aim to objectively determine which aspects of fear learning are modulated by clinically effective SSRIs. This information may improve translation between human and animal research since it may enable informed choices for the use of particular anxiety tests and procedures. In addition, results from the systematic review may contribute to a better understanding of the role of the serotonergic system in fear acquisition and extinction learning and retrieval.	
	Research question		
11.	Specify the disease/health problem of interest	Anxiety-related disorders	
12.	Specify the population/species studied	All species, human (healthy and diagnosed with anxiety-related disorder) and non-human animals (naïve and disease induction)	

13.	Specify the intervention/exposure	Treatment with one of the six following clinically relevant SSRIs (fluoxetine, fluvoxamine, sertraline, citalopram, escitalopram, paroxetine)	
14.	Specify the control population	Subject treated with placebo undergoing similar anxiety test and disease-induction (if applicable)	
15.	Specify the outcome measures	Levels of anxiety in tests for fear acquisition learning and retrieval and in tests for fear extinction learning and retrieval	
16.	State your research question (based on items 11-15)	What is the effect of clinically effective SSRIs on fear learning?	
	C. Methods		
	Search and study identification		
17.	Identify literature databases to search (*e.g.* Pubmed, Embase, Web of science)	■ MEDLINE via PubMed ☐ Web of Science<br>☐ SCOPUS ■ EMBASE<br>☐ Other, namely:<br>☐ Specific journal(s), namely:	
18.	Define electronic search strategies (*e.g.* use the step by step search guide[15] and animal search filters[20, 21])	When available, please add a supplementary file containing your search strategy: see supplementary file S2	
19.	Identify other sources for study identification	■ Reference lists of included studies ☐ Books<br>■ Reference lists of relevant reviews<br>☐ Conference proceedings, namely:<br>☐ Contacting authors/ organisations, namely:<br>☐ Other, namely:	
20.	Define search strategy for these other sources	Reference lists will be screened for interesting titles. Relevance of papers will be screened in the same way as performed in the papers retrieved by initial search.	
	Study selection		
21.	Define screening phases (*e.g.* pre-screening based on title/abstract, full text screening, both)	After removal of duplications<br>- First phase: Screening on title and abstract on in and exclusion criteria<br>- Second phase: Screening full text on in and exclusion criteria	
22.	Specify (a) the number of reviewers per screening phase and (b) how discrepancies will be resolved	2 reviewers (EH, MV) will cover each screening phase. In case differences in screening occur, a third reviewer will be consulted (EYB or LG)	

	Define all inclusion and exclusion criteria based on:		
23.	Type of study (design)	Inclusion criteria:<br>- original peer reviewed published studies<br>- controlled studies<br>Exclusion criteria:<br>- Non original studies (reviews, case reports etc)<br>- Studies without a control group	
24.	Type of animals/population (*e.g.* age, gender, disease model)	Inclusion criteria:<br>- all human (healthy and diagnosed with anxiety-related disorder) and non-human animals (naïve and disease induction)<br>Exclusion criteria:<br>- In vitro, ex vivo<br>- Non-animal or non-human related research	
25.	Type of intervention (*e.g.* dosage, timing, frequency)	Inclusion criteria:<br>- Clinically effective SSRI (fluoxetine, fluvoxamine, sertraline, citalopram, escitalopram, paroxetine) with an adequate placebo-treated control group,<br>- All dosing schedules (that is: no restrictions on dose, timing or frequency)<br>- Acquisition and/or extinction studies where the SSRI was given up until or within 24 hours of measuring acquisition or extinction learning/retrieval.<br><br>Exclusion criteria:<br>- Any other treatment than one of the six following SSRIs (fluoxetine, fluvoxamine, sertraline, citalopram, escitalopram, paroxetine),<br>- Combined treatment with any other drug<br>- Acquisition and/or extinction studies where the SSRI was not given up until or within 24 hours of measuring acquisition or extinction learning/retrieval.	
26.	Outcome measures	Inclusion criteria:<br>- Any outcome parameter (e.g. freezing behavior, startle reflex) assessing the level of anxiety in tests for fear acquisition learning and retrieval and in tests for fear extinction learning and retrieval. Including tests such as classical fear conditioning, passive avoidance, active avoidance, conditioned emotional response and contextual and discrete conditioning.<br><br>Exclusion criteria:<br>- Outcome not assessing the level of anxiety in tests for fear acquisition learning and retrieval and in tests for fear extinction learning and retrieval	

27.	Language restrictions	Inclusion criteria: English or Dutch publications<br><br>Exclusion criteria: non-English or non-Dutch publications	
28.	Publication date restrictions	No restrictions	
29.	Other	-	
30.	Sort and prioritize your exclusion criteria per selection phase	Selection phase I: title and abstract<br><br>1. Not an original full publication (abstract, review)<br>2. Not an *in vivo* animal study or human study but *in vitro, ex vivo*<br>3. Not an article about fear learning<br>4. Not a clinically defined effective SSRI treatment used<br>5. SSRI not directly given to study subject<br><br>Selection phase II: full text<br><br>As above, with the addition of:<br><br>6. No appropriate placebo or vehicle-controlled experiment<br>7. No information available/retrievable within one reference on the protocol of fear learning<br>8. No information available/retrievable on specific SSRI used<br>9. No results reported from testing fear learning<br>10. Use of an additional pharmacological treatment before/during/after SSRI treatment and before testing fear learning<br>11. SSRI treatment not tested on fear learning<br>12. Acquisition and/or extinction studies where the SSRI was not given up until or within 24 hours of measuring acquisition or extinction learning/retrieval<br>13. Full article text not retrievable	
	Study characteristics to be extracted (for assessment of external validity, reporting quality)		
31.	Study ID (*e.g.* authors, year)	- Author<br>- Title<br>- Year of publication	
32.	Study design characteristics (*e.g.* experimental groups, number of animals)	- Housing (single /group/NR)<br>- Time of test (active (schedule) /passive/ NR)<br>- Day/night schedule (normal (light during daytime) /reversed (dark during daytime)/ NR)	
33.	Animal model characteristics (*e.g.* species, gender, disease induction)	- Species (mouse, rat, guinea pig, ... NR)<br>- Strain (…, NR)<br>- Age at time of testing<br>- (and body weight at time of testing (if not available [[at arrival]])<br>- Sex (m/f/mix/NR)	

		- Disease induction (prior stress (NR, no, acute, chronic (>1); what), GMO (y/n; what), other (y/n; what)	
34.	Intervention characteristics (*e.g.* intervention, timing, duration)	SSRI treatment:<br>- SSRI<br>- Doses<br>- Route<br>- Injection test interval<br>- Frequency (acute/chronic treatment)<br>For chronic treatment specify:<br>• # days<br>• treated during disease induction (y/n)<br>• treated on test day (y/n)	
35.	Outcome measures	Number of subjects per experimental condition (intervention and control)<br><br><u>Fear learning:</u><br>Outcome behaviour as reported (<u>document behaviour</u> (!))<br>*includes freezing, startle, immobility, vocalizations, other..*<br>- Most effective dose (= ... mg/kg); defined as largest difference between placebo treated and experimental group<br><br>- acquisition learning (to cue)<br>- acquisition learning (to context)<br>- acquisition retrieval (to cue)<br>- acquisition retrieval (to context)<br><br>- extinction learning (to cue)<br>- extinction learning (to context)<br>- extinction retrieval (to cue)<br>- extinction retrieval (to context)<br><br>- Type of test<br>- Type of behaviour	
36.	Other (*e.g.* drop-outs)	-	
	Assessment risk of bias (internal validity) or study quality		
37.	Specify (a) the number of reviewers assessing the risk of bias/study quality in each study and (b) how discrepancies will be resolved	Two reviewers will assess the risk of bias. In case differences in assessment occur, a third reviewer will be consulted.	

38.	Define criteria to assess (a) the internal validity of included studies (*e.g.* selection, performance, detection and attrition bias) and/or (b) other study quality measures (*e.g.* reporting quality, power)	☐By use of SYRCLE's Risk of Bias tool[4]<br><br>■By use of SYRCLE's Risk of Bias tool, adapted as follows:<br><br>- Reporting questions related to randomization/blinding<br><br>☐By use of CAMARADES' study quality checklist, e.g [22]<br><br>☐By use of CAMARADES' study quality checklist, adapted as follows:<br><br>☐Other criteria, namely:	
	Collection of outcome data		
39.	For each outcome measure, define the type of data to be extracted (*e.g.* continuous/dichotomous, unit of measurement)	We expect the unit of measurement to be variable and the type of data that needs to be extracted to be continuous.	
40.	Methods for data extraction/retrieval (*e.g.* first extraction from graphs using a digital screen ruler, then contacting authors)	Data extraction from text, tables and graphs. Defined as largest difference between placebo treated and experimental group. If necessary, contact authors. In case data is not exactly mentioned in the text we will extract data from graphs using a digital screen ruler, if that is not possible either we will try to contact the authors	
41.	Specify (a) the number of reviewers extracting data and (b) how discrepancies will be resolved	One reviewer will extract the data and the second reviewer will check these extracted data.	
	Data analysis/synthesis		
42.	Specify (per outcome measure) how you are planning to combine/compare the data (*e.g.* descriptive summary, meta-analysis)	Same for acquisition learning and retrieval and extinction learning and retrieval:<br><br>- Meta-analysis is limited to outcome measures reported in at least 10 independent studies to ensure sufficient statistical power.<br>- If there is insufficient studies to perform a meta-analysis we will perform a descriptive analyse, reporting the outcome of the most effective dose (no effect, increase, decrease). Figures will be made to integrate different aspects of the reported studies.	
43.	Specify (per outcome measure) how it will be decided whether a meta-analysis will be performed	Same for acquisition learning and retrieval and extinction learning and retrieval: A subgroup analysis will be performed if five or more independent comparisons from at least three different references are included reporting the same or similar outcome measures (acquisition, extinction).	

	If a meta-analysis seems feasible/sensible, specify (for each outcome measure):		
44.	The effect measure to be used (*e.g.* mean difference, standardized mean difference, risk ratio, odds ratio)	Standardized mean difference	
45.	The statistical model of analysis (*e.g.* random or fixed effects model)	Random effects model	
46.	The statistical methods to assess heterogeneity (*e.g.* I^2, Q)	Heterogeneity will be assessed using I^2 values	
47.	Which study characteristics will be examined as potential source of heterogeneity (subgroup analysis)	- Acute versus chronic treatment<br>- Disease induction versus healthy subjects<br>- Species<br>- Type of test used to assess outcome measure<br>- Type of SSRI used	
48.	Any sensitivity analyses you propose to perform	To be determined (SSRI, if not enough data to analyse each SSRI separately)	
49.	Other details meta-analysis (*e.g.* correction for multiple testing, correction for multiple use of control group)	Bonferroni correction for multiple testing and correction for multiple use of the control group (nr of animals/nr of times the control group is used)	
50.	The method for assessment of publication bias	Visual inspection of the funnel plot, Egger's Regression Test and Duval and Tweedie's Trim and Fill. For standardized mean differences, we will use an n-based precision estimate to avoid distortion of the funnel plots. A minimum of 15 studies (for each outcome) will be required to analyse publication bias.	
Final approval by (names, affiliations):		Date:	
