# Supplementary material for: The effect of SSRIs on fear learning: a systematic review and meta-analysis
Source: Psychopharmacology (Berl). 2023 Feb 27;240(11):2335–59. doi: 10.1007/s00213-023-06333-7 (PMC10593621; doi:10.1007/s00213-023-06333-7)
Supplement: Supplementary file 2 — Supplementary file2 (PDF 168 KB) [file 213_2023_6333_MOESM2_ESM.pdf]

The effect of SSRIs on fear learning: a systematic review and meta-analysis

Psychopharmacology

Elise J Heesbeen, Elisabeth Y Bijlsma, P Monika Verdouw, Caspar van Lissa, Carlijn Hooijmans, Lucianne Groenink

Corresponding author: Lucianne Groenink, l.groenink@uu.nl

Supplementary file S2. Search strategy of the systematic review demonstrating the two included components (SSRIs and fear learning) for the two searched databases (PubMed and Embase).

Database	*Component*	*Search strategy*
PubMed	SSRI	(citalopram[MeSH] OR citalopram*[Title/Abstract] OR Cytalopram[Title/Abstract] OR Seropram[Title/Abstract] OR Celexa[Title/Abstract] OR Lu-10-171[Title/Abstract] OR Lu10171[Title/Abstract] OR Lexapro[Title/Abstract] OR escitalopram*[Title/Abstract] OR fluoxetine[MeSH] OR fluoxetin*[Title/Abstract] OR "N-Methyl-gamma-(4-(trifluoromethyl)phenoxy)benzenepropanamine"[Title/Abstract] OR "Lilly-110140"[Title/Abstract] OR "Lilly110140"[Title/Abstract] OR Sarafem[Title/Abstract] OR Prozac[Title/Abstract] OR fluvoxamine[MeSH] OR fluvoxamin*[Title/Abstract] OR Fluvoxadura[Title/Abstract] OR Luvox[Title/Abstract] OR Floxyfral[Title/Abstract] OR Fevarin[Title/Abstract] OR Dumirox[Title/Abstract] OR Faverin[Title/Abstract] OR Desiflu[Title/Abstract] OR "DU-23000"[Title/Abstract] OR "DU23000"[Title/Abstract] OR paroxetine[MeSH] OR paroxetin*[Title/Abstract] OR Aropax[Title/Abstract] OR "BRL-29060"[Title/Abstract] OR "BRL29060"[Title/Abstract] OR "FG-7051"[Title/Abstract] OR "FG7051"[Title/Abstract] OR Seroxat[Title/Abstract] OR "Paxil"[Title/Abstract] OR sertraline[MeSH] OR sertralin*[Title/Abstract] OR Zoloft[Title/Abstract] OR Altruline[Title/Abstract] OR Lustral[Title/Abstract] OR Aremis[Title/Abstract] OR Besitran[Title/Abstract] OR Sealdin[Title/Abstract] OR Gladem[Title/Abstract])
PubMed	Fear learning	(acquisition[Title/Abstract] OR extinction[Title/Abstract] OR "Extinction, Psychological"[Mesh] OR learn*[Title/Abstract] OR "Learning"[Mesh] OR condition*[Title/Abstract] OR "Conditioning, Classical"[Mesh] OR "Cues"[Mesh] OR memor*[Title/Abstract] OR memory[Mesh] OR avoidance[Title/Abstract] OR "Avoidance Learning"[Mesh] OR retrieval[Title/Abstract] OR freezing[Title/Abstract] OR "Freezing Reaction, Cataleptic"[Mesh] OR “foot shock*”[Title/Abstract] OR “electric shock*”[Title/Abstract] OR

		“air puff*”[Title/Abstract] OR tone*[Title/Abstract] OR noise*[Title/Abstract] OR “startle reflex*”[Title/Abstract] OR "Reflex, Startle"[Mesh] OR cue[Title/Abstract] OR cued[Title/Abstract] OR cues[Title/Abstract] OR context*[Title/Abstract] OR “skin conductance*”[Title/Abstract] OR "Galvanic Skin Response"[Mesh] OR “heart rate*”[Title/Abstract] OR "Heart Rate"[Mesh] OR “communication*”[Title/Abstract] OR "Communication"[Mesh] OR VAS[Title/Abstract] OR “Visual Analogue Scale*”[Title/Abstract] OR “Visual Analog Scale*”[Title/Abstract] OR "Visual Analog Scale"[Mesh] OR “survey*”[Title/Abstract] OR "Health Care Surveys"[Mesh] OR "Patient Health Questionnaire"[Mesh]) AND (fear[Title/Abstract] OR fear [MeSH] OR anxiety [Title/Abstract] OR anxiety [MeSH])
Embase	SSRI	‘citalopram’/exp OR ‘citalopram*’:ab,ti,kw OR ‘1 3 dimethylaminopropyl 1 4 fluorophenyl 1 3 dihydroisobenzofuran 5 carbonitrile’:ab,ti,kw OR ‘1 3 dimethylaminopropyl 1 4 fluorophenyl 5 phthalancarbonitrile’:ab,ti,kw OR ‘1 3 dimethylaminopropyl 1, 3 dihydro 1 4 fluorophenyl isobenzofuran 5 carbonitrile’:ab,ti,kw OR ‘5 phthalancarbonitrile 1 3 dimethylaminopropyl 1 4 fluorophenyl’:ab,ti,kw OR ‘acelopam’:ab,ti,kw OR ‘adeprenal’:ab,ti,kw OR ‘apo-cital’:ab,ti,kw OR ‘aurex’:ab,ti,kw OR ‘celexa’:ab,ti,kw OR ‘cilopress’:ab,ti,kw OR ‘cinavol’:ab,ti,kw OR ‘ciprager’:ab,ti,kw OR ‘cipram’:ab,ti,kw OR ‘cipramil’:ab,ti,kw OR ‘cipraned’:ab,ti,kw OR ‘ciprotan’:ab,ti,kw OR ‘ciral’:ab,ti,kw OR ‘citabax’:ab,ti,kw OR ‘citacip’:ab,ti,kw OR ‘citagen’:ab,ti,kw OR ‘cital’:ab,ti,kw OR ‘citalec’:ab,ti,kw OR ‘citalich’:ab,ti,kw OR ‘citalon’:ab,ti,kw OR ‘citalonte’:ab,ti,kw OR ‘citalostad’:ab,ti,kw OR ‘citalox’:ab,ti,kw OR ‘citalvir’:ab,ti,kw OR ‘citapram’:ab,ti,kw OR ‘citaxin’:ab,ti,kw OR ‘citesint’:ab,ti,kw OR ‘citopam’:ab,ti,kw OR ‘citrol’:ab,ti,kw OR ‘citronil’:ab,ti,kw OR ‘cytalopram’:ab,ti,kw OR ‘dalsan’:ab,ti,kw OR ‘elopram’:ab,ti,kw

OR ‘exenadil’:ab,ti,kw OR ‘frimaind’:ab,ti,kw OR ‘futuril’:ab,ti,kw OR ‘galopran’:ab,ti,kw OR ‘humorap’:ab,ti,kw OR ‘kaidor’:ab,ti,kw OR ‘kitapram’:ab,ti,kw OR ‘linisan’:ab,ti,kw OR ‘lopracil’:ab,ti,kw OR ‘lopraxer’:ab,ti,kw OR ‘loxopram’:ab,ti,kw OR ‘lu 10171’:ab,ti,kw OR ‘lu10 171’:ab,ti,kw OR ‘lu10171’:ab,ti,kw OR ‘lupram’:ab,ti,kw OR ‘malicon’:ab,ti,kw OR ‘nitalapram’:ab,ti,kw OR ‘oropram’:ab,ti,kw OR ‘percitale’:ab,ti,kw OR ‘pralotam’:ab,ti,kw OR ‘pramital’:ab,ti,kw OR ‘prefucet’:ab,ti,kw OR ‘pricital’:ab,ti,kw OR ‘prisdal’:ab,ti,kw OR ‘psiconor’:ab,ti,kw OR ‘renevil’:ab,ti,kw OR ‘ricap’:ab,ti,kw OR ‘ropramin’:ab,ti,kw OR ‘selon’:ab,ti,kw OR ‘sepram’:ab,ti,kw OR ‘seralgan’:ab,ti,kw OR ‘seregra’:ab,ti,kw OR ‘serital’:ab,ti,kw OR ‘seropram’:ab,ti,kw OR ‘seror’:ab,ti,kw OR ‘sintopram’:ab,ti,kw OR ‘sotovon’:ab,ti,kw OR ‘talam’:ab,ti,kw OR ‘talosin’:ab,ti,kw OR ‘varom’:ab,ti,kw OR ‘vesema’:ab,ti,kw OR ‘xadorek’:ab,ti,kw OR ‘zanipram’:ab,ti,kw OR ‘zd 211’:ab,ti,kw OR ‘zd211’:ab,ti,kw OR ‘zeclicid’:ab,ti,kw OR ‘zentius’:ab,ti,kw OR ‘zitolex’:ab,ti,kw OR ‘zyloram’:ab,ti,kw OR ‘escitalopram’/exp OR ‘escitalopram*’:ab,ti,kw OR ‘cipralex’:ab,ti,kw OR ‘entact’:ab,ti,kw OR ‘esciprex’:ab,ti,kw OR ‘lexapro’:ab,ti,kw OR ‘lu 26054 0’:ab,ti,kw OR ‘lu 260540’:ab,ti,kw OR ‘lu260540’:ab,ti,kw OR ‘premalex’:ab,ti,kw OR ‘prilect’:ab,ti,kw OR ‘seroplex’:ab,ti,kw OR ‘sipralexa’:ab,ti,kw OR ‘zecidec’:ab,ti,kw OR ‘zocital’:ab,ti,kw OR ‘fluoxetine’/exp OR ‘fluoxetin*’:ab,ti,kw OR ‘3 4 trifluoromethylphenoxy n methyl 3 phenylpropylamine’:ab,ti,kw OR ‘3 n methyl 3 phenyl 3 4 trifluoromethylphenoxy propylamine’:ab,ti,kw OR ‘actan’:ab,ti,kw OR ‘adofen’:ab,ti,kw OR ‘afeksin’:ab,ti,kw OR ‘alzac 20’:ab,ti,kw OR ‘andep’:ab,ti,kw OR ‘andepin’:ab,ti,kw OR ‘ansilan’:ab,ti,kw OR ‘atd 20’:ab,ti,kw OR ‘auroken’:ab,ti,kw OR ‘auscap’:ab,ti,kw OR ‘bioxetin’:ab,ti,kw OR ‘captaton’:ab,ti,kw OR ‘compound 110140’:ab,ti,kw OR ‘daforin’:ab,ti,kw OR ‘dagrilan’:ab,ti,kw OR ‘depren’:ab,ti,kw OR ‘deprex’:ab,ti,kw OR ‘deprex

	leciva':ab,ti,kw OR 'deprexetin':ab,ti,kw OR 'deprexin':ab,ti,kw OR 'deprizac':ab,ti,kw OR 'deproxin':ab,ti,kw OR 'diesan':ab,ti,kw OR 'digassim':ab,ti,kw OR 'elizac':ab,ti,kw OR 'exostrept':ab,ti,kw OR 'felicium':ab,ti,kw OR 'fldiss':ab,ti,kw OR 'flotinal':ab,ti,kw OR 'floxet':ab,ti,kw OR 'fluctin':ab,ti,kw OR 'fluctine':ab,ti,kw OR 'fludac':ab,ti,kw OR 'flufran':ab,ti,kw OR 'fluketin':ab,ti,kw OR 'flunil':ab,ti,kw OR 'flunirin':ab,ti,kw OR 'fluohexal':ab,ti,kw OR 'fluoksetin':ab,ti,kw OR 'fluoksetyna':ab,ti,kw OR 'fluox':ab,ti,kw OR 'fluox-puren':ab,ti,kw OR 'fluoxac':ab,ti,kw OR 'fluoxeren':ab,ti,kw OR 'fluoxifar':ab,ti,kw OR 'fluoxil':ab,ti,kw OR 'fluoxone':ab,ti,kw OR 'fluoxtab':ab,ti,kw OR 'fluronin':ab,ti,kw OR 'flusac':ab,ti,kw OR 'flustad':ab,ti,kw OR 'flutin':ab,ti,kw OR 'flutine':ab,ti,kw OR 'flux':ab,ti,kw OR 'fluxemed':ab,ti,kw OR 'fluxen':ab,ti,kw OR 'fluxet':ab,ti,kw OR 'fluxetil':ab,ti,kw OR 'fluxetin':ab,ti,kw OR 'fluxil':ab,ti,kw OR 'fluxomed':ab,ti,kw OR 'fluzac':ab,ti,kw OR 'fokeston':ab,ti,kw OR 'fontex':ab,ti,kw OR 'foxetin':ab,ti,kw OR 'foxtin':ab,ti,kw OR 'fropine':ab,ti,kw OR 'fuloren':ab,ti,kw OR 'gerozac':ab,ti,kw OR 'ladose':ab,ti,kw OR 'lanclic':ab,ti,kw OR 'lilly 110140':ab,ti,kw OR 'lilly110140':ab,ti,kw OR 'lorien':ab,ti,kw OR 'lovan':ab,ti,kw OR 'luramon':ab,ti,kw OR 'ly 110140':ab,ti,kw OR 'ly110140':ab,ti,kw OR 'magrilan':ab,ti,kw OR 'margrilan':ab,ti,kw OR 'meropan':ab,ti,kw OR 'modipran':ab,ti,kw OR 'mutan':ab,ti,kw OR 'n methyl 3 phenyl 3 (4 trifluoromethylphenoxy) propylamine':ab,ti,kw OR 'n methyl 3 phenyl 3 [(alpha, alpha, alpha trifluoro para tolyl) oxy] propylamine':ab,ti,kw OR 'nopres':ab,ti,kw OR 'nuzak':ab,ti,kw OR 'olena':ab,ti,kw OR 'oxactin':ab,ti,kw OR 'oxedep':ab,ti,kw OR 'phenylpropylamine, n methyl 3 (4 trifluoromethylphenoxy)':ab,ti,kw OR 'plazeron':ab,ti,kw OR 'plinzene':ab,ti,kw OR 'pragmaten':ab,ti,kw OR 'prizma':ab,ti,kw OR 'proctin':ab,ti,kw OR 'prodep':ab,ti,kw OR 'prosac':ab,ti,kw OR 'prozac':ab,ti,kw OR 'prozamel':ab,ti,kw OR

‘prozamin’:ab,ti,kw OR ‘prozep’:ab,ti,kw OR ‘prozit’:ab,ti,kw OR ‘psipax’:ab,ti,kw OR ‘qualisac’:ab,ti,kw OR ‘rapiflux’:ab,ti,kw OR ‘reconcile’:ab,ti,kw OR ‘reneuron’:ab,ti,kw OR ‘rowexetina’:ab,ti,kw OR ‘salipax’:ab,ti,kw OR ‘sanzur’:ab,ti,kw OR ‘sarafem’:ab,ti,kw OR ‘sartuzin’:ab,ti,kw OR ‘selfemra’:ab,ti,kw OR ‘seromex’:ab,ti,kw OR ‘seronil’:ab,ti,kw OR ‘sinzac’:ab,ti,kw OR ‘sofelin’:ab,ti,kw OR ‘stephadilat-s’:ab,ti,kw OR ‘xeredien’:ab,ti,kw OR ‘zactin’:ab,ti,kw OR ‘zepax’:ab,ti,kw OR ‘zinovat’:ab,ti,kw OR ‘fluvoxamine’/exp OR ‘fluvoxamin*’:ab,ti,kw OR ‘5 methoxy 1 [4 (trifluoromethyl) phenyl] 1 pentanone o (2 aminoethyl) oxime’:ab,ti,kw OR ‘5 methoxy 4 (trifluoromethyl) valerophenone o (2 aminoethyl) oxime’:ab,ti,kw OR ‘du 23000’:ab,ti,kw OR ‘du23000’:ab,ti,kw OR ‘fluoxamine’:ab,ti,kw OR ‘fluroxamine’:ab,ti,kw OR ‘paroxetine’/exp OR ‘paroxetin*’:ab,ti,kw OR ‘4 (4 fluorophenyl) 3 [(3, 4 methylenedioxyphenoxy) methyl] piperidine’:ab,ti,kw OR ‘arketis’:ab,ti,kw OR ‘aropax’:ab,ti,kw OR ‘aroxat’:ab,ti,kw OR ‘brisdelle’:ab,ti,kw OR ‘brl 29060’:ab,ti,kw OR ‘brl 29060a’:ab,ti,kw OR ‘brl29060’:ab,ti,kw OR ‘brl29060a’:ab,ti,kw OR ‘daparox’:ab,ti,kw OR ‘deroxat’:ab,ti,kw OR ‘dexorat’:ab,ti,kw OR ‘divarius’:ab,ti,kw OR ‘dropax’:ab,ti,kw OR ‘euplix’:ab,ti,kw OR ‘eutimil’:ab,ti,kw OR ‘fg 7051’:ab,ti,kw OR ‘fg7051’:ab,ti,kw OR ‘frosinor’:ab,ti,kw OR ‘motivan’:ab,ti,kw OR ‘optipar’:ab,ti,kw OR ‘paluxetil’:ab,ti,kw OR ‘paluxon’:ab,ti,kw OR ‘paroc’:ab,ti,kw OR ‘parogen’:ab,ti,kw OR ‘paroxedura’:ab,ti,kw OR ‘paroxet’:ab,ti,kw OR ‘paroxia’:ab,ti,kw OR ‘paxan’:ab,ti,kw OR ‘paxil’:ab,ti,kw OR ‘paxtine’:ab,ti,kw OR ‘paxxet’:ab,ti,kw OR ‘pexeva’:ab,ti,kw OR ‘sereupin’:ab,ti,kw OR ‘seroxat’:ab,ti,kw OR ‘setine’:ab,ti,kw OR ‘si 211103’:ab,ti,kw OR ‘si211103’:ab,ti,kw OR ‘solben’:ab,ti,kw OR ‘syntopar’:ab,ti,kw OR ‘tagonis’:ab,ti,kw OR ‘sertraline’/exp OR ‘sertralin*’:ab,ti,kw OR ‘1 methylamino 4 (3, 4 dichlorophenyl) tetralin’:ab,ti,kw OR ‘altruline’:ab,ti,kw OR ‘aremis’:ab,ti,kw OR ‘atruline’:ab,ti,kw OR ‘besitran’:ab,ti,kw OR

		‘cp 51974’:ab,ti,kw OR ‘cp 51974 01’:ab,ti,kw OR ‘cp 51974 1’:ab,ti,kw OR ‘cp 519741’:ab,ti,kw OR ‘cp51974’:ab,ti,kw OR ‘cp51974 01’:ab,ti,kw OR ‘cp51974 1’:ab,ti,kw OR ‘cp5197401’:ab,ti,kw OR ‘cp519741’:ab,ti,kw OR ‘dominum’:ab,ti,kw OR ‘doxime’:ab,ti,kw OR ‘fatral’:ab,ti,kw OR ‘fridep’:ab,ti,kw OR ‘gladem’:ab,ti,kw OR ‘lesefer’:ab,ti,kw OR ‘lustral’:ab,ti,kw OR ‘n methyl 4 (3, 4 dichlorophenyl) 1, 2, 3, 4 tetrahydro 1 naphthylamine’:ab,ti,kw OR ‘nudep’:ab,ti,kw OR ‘seltra’:ab,ti,kw OR ‘serad’:ab,ti,kw OR ‘sercerin’:ab,ti,kw OR ‘serlain’:ab,ti,kw OR ‘serlift’:ab,ti,kw OR ‘sertralin’:ab,ti,kw OR ‘sertranex’:ab,ti,kw OR ‘sertranquil’:ab,ti,kw OR ‘sosser’:ab,ti,kw OR ‘tatig’:ab,ti,kw OR ‘tresleen’:ab,ti,kw OR ‘zolof’:ab,ti,kw OR ‘zoloft’:ab,ti,kw OR ‘zosert’:ab,ti,kw
Embase	Fear learning	(‘acquisition’:ab,ti,kw OR ‘extinction’:ab,ti,kw OR ‘reinforcement’/exp OR ‘learn*’:ab,ti,kw OR ‘learning’/exp OR ‘condition*’:ab,ti,kw OR ‘conditioning’/exp OR ‘memor*’:ab,ti,kw OR ‘memory’/exp OR ‘avoidance’:ab,ti,kw OR ‘avoidance behavior’/exp OR ‘retrieval’:ab,ti,kw OR ‘information retrieval’/exp OR ‘freezing’:ab,ti,kw OR ‘catalepsy’/exp OR ‘foot shock*’:ab,ti,kw OR ‘electric shock*’:ab,ti,kw OR ‘air puff*’:ab,ti,kw OR ‘tone*’:ab,ti,kw OR ‘noise*’:ab,ti,kw OR ‘startle reflex*’:ab,ti,kw OR ‘startle reflex’/exp OR ‘cue’:ab,ti,kw OR ‘cued’:ab,ti,kw OR ‘cues’:ab,ti,kw OR ‘context*’:ab,ti,kw OR ‘skin conductance*’:ab,ti,kw OR ‘skin conductance’/exp OR ‘electrodermal response’/exp OR ‘heart rate*’:ab,ti,kw OR ‘heart rate’/exp OR ‘communication*’:ab,ti,kw OR ‘interpersonal communication’/exp OR VAS:ab,ti,kw OR ‘Visual Analogue Scale*’:ab,ti,kw OR ‘Visual Analog Scale*’:ab,ti,kw OR ‘visual analog scale’/exp OR ‘survey*’:ab,ti,kw OR ‘health survey’/exp OR ‘questionnaire’/exp) AND (fear:ab,ti,kw OR fear/exp OR anxiety:ab,ti,kw OR anxiety/exp)
