# Supplementary material for: The effect of SSRIs on fear learning: a systematic review and meta-analysis
Source: Psychopharmacology (Berl). 2023 Feb 27;240(11):2335–59. doi: 10.1007/s00213-023-06333-7 (PMC10593621; doi:10.1007/s00213-023-06333-7)
Supplement: Supplementary file 4 — Supplementary file4 (PDF 1059 KB) [file 213_2023_6333_MOESM4_ESM.pdf]

The effect of SSRIs on fear learning: a systematic review and meta-analysis

Psychopharmacology

Elise J Heesbeen, Elisabeth Y Bijlsma, P Monika Verdouw, Caspar van Lissa, Carlijn Hooijmans, Lucianne Groenink

Corresponding author: Lucianne Groenink, l.groenink@uu.nl

Supplementary file S4. Methods and results of meta-analysis performed with CMA software

Materials and Methods

Data-analysis

The Comprehensive Meta-Analysis (CMA) V3.3 software was used to perform the statistical analysis the accompanying forest plots were produced in GraphPad Prism 9. The Hedges's g standardized mean difference (SMD) was used as the effect size measure.

In case of multiple use of the same control group the number of animals used in that control group was divided by the number of times that control group was used in the meta-analysis. A meta-analysis was conducted for each fear learning process that was reported in ten or more independent comparisons from at least five different references. We used the random effects model which takes into account the precision of individual studies and the variation between studies and weights each study accordingly. An overall SMD with a 95% confidence interval was calculated for each of these fear learning processes by pooling all the individual effect sizes (SMDs). A random effects model was used since a variation in the true effect was expected between experiments.

Moderators were predefined (type of SSRI, frequency of administration, species, disease induction and type of fear learning test) and registered in the protocol (supplementary file S1). Subgroup analyses were performed within these moderators whenever five or more independent comparisons from at least three different references were included reporting the same fear learning outcome measures (acquisition/extinction learning or fear expression (after acquisition or extinction learning) to cue/context). Comparisons between subgroups were made by calculating the SMD and the 95% confidence interval of the difference (ΔSMD and Δ95% CI) between the subgroups. We expected the variance to be comparable within the subgroups; therefore, we assumed a common among-study variance across subgroups. For subgroup analyses, we adjusted our significance level according to the conservative Bonferroni method to account for multiple analyses (p* number of comparisons). However, differences between subgroups should be interpreted with caution and should only be used for constructing new hypotheses rather than for drawing final conclusions. In addition, heterogeneity was evaluated by measuring the I^2 value, the ratio of true heterogeneity to total variance across the observed effect estimates.

Results

Acquisition learning to cue

Meta-analysis

A total of 39 experiments (k = 39) were included in the meta-analysis that investigated the effect of SSRIs on the acquisition learning to cue. Of these 39 experiments, 24 experiments measured acquisition learning to cue, and 15 experiments acquisition learning measured as expression to cue. No effect of SSRIs on acquisition learning to cue was observed (SMD (95% CI) 0.02 (-0.32, 0.35); I^2 81%; k = 39) (figure 7A).

None of the four SSRIs that could be included in the subgroup analysis had a significant effect on acquisition learning to cue: citalopram (SMD (95% CI) 0.19 (-0.37, 0.75); P=0.50; I^2 75%; k = 8), escitalopram (SMD (95% CI) -0.19 (-0.90, 0.51); P=0.59; I^2 45%; k = 5), fluoxetine (SMD (95% CI) 0.19 (-0.20, 0.57); P=0.35; I^2 62%; k = 18) and paroxetine (SMD (95% CI) 0.06 (-0.66, 0.77); P=0.88; I^2 85%; k = 5) (figure 7B).

Subgroup analysis showed that acute (SMD (95% CI) 0.50 (0.01, 0.99); P=0.04; I^2 51%; k = 17) SSRI administration significantly increased fear behaviour during acquisition learning to cue. However, chronic SSRI administration had no significant effect on acquisition learning to cue (SMD (95% CI) -0.37 (-0.84, 0.10); P=0.12; I^2 87%; k = 20). Additional analysis revealed that acute SSRI administration had a significantly different effect on fear behaviour than chronic SSRI administration (ΔSMD (95% CI) 0.87 (0.19, 1.55); P=0.02) (figure 7B).

Subgroup analysis of disease induction showed no significant effect of SSRIs on acquisition learning to cue in healthy (SMD (95% CI) 0.07 (-0.31, 0.44); P=0.72; I^2 78%; k = 29) and stressed subjects (SMD (95% CI) 0.11 (-0.59, 0.81); P=0.76; I^2 86%; k = 9)(figure 7B).

A subgroup analysis of species revealed no significant effect of SSRIs on acquisition learning to cue in mice (SMD (95% CI) -0.56 (-1.40, 0.28); P=0.19; I^2 87%; k = 7) nor in rats (SMD (95% CI) 0.19 (-0.25, 0.62); P=0.40; I^2 81%; k = 28) (figure 7B).

Subgroup analysis comparing different types of fear learning tests revealed no significant effect of SSRIs on acquisition learning neither in the active avoidance (SMD (95% CI) -0.17 (-1.24, 0.90); P=0.76; I^2 95%; k = 5) nor in the conditioned freezing test (SMD (95% CI) 0.02 (-0.38, 0.42); P=0.93; I^2 76%; k = 31) (figure 7B).

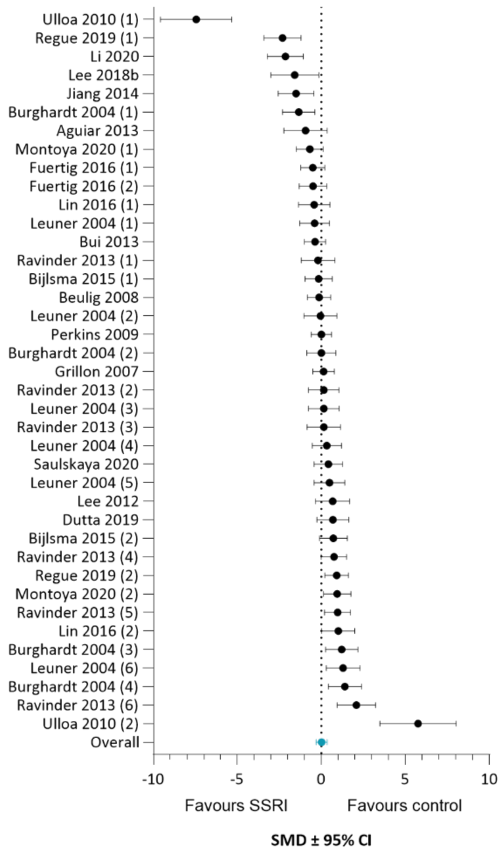

Type of SSRI	Frequency	Disease induction	Species	Type of test
Sertraline	Chronic [daily, 13x]	None	Rat	Active avoidance
Paroxetine	Chronic [daily, 28x]	VGV 5-HT2CR	Mouse	Conditioned freezing
Sertraline	Chronic [daily, 12x]	Single prolonged stress	Mouse	Conditioned freezing
Fluoxetine	Chronic [daily, 14x]	Single prolonged stress	Rat	Conditioned freezing
Fluoxetine	Chronic [daily, 14x]	None	Rat	Conditioned freezing
Citalopram	Chronic [daily, 22x]	None	Rat	Conditioned freezing
Fluoxetine	Chronic [daily, 19x]	None	Rat	Conditioned freezing
Citalopram	Acute	None	Mouse	Conditioned freezing
Escitalopram	Subchronic [daily, 3x]	Chronic social defeat	Mouse	Conditioned freezing
Escitalopram	Subchronic [daily, 3x]	None	Mouse	Conditioned freezing
Escitalopram	Chronic [daily, 14x]	Single prolonged stress	Rat	Conditioned freezing
Fluoxetine	Chronic [daily, minimally 14x]	None	Rat	Conditioned freezing
Escitalopram	Chronic [daily, 14x]	None	Human	Conditioned freezing
Fluoxetine	Acute	None	Rat	Conditioned freezing
Paroxetine	Chronic [daily, 21x]	None	Rat	Fear potentiated startle
Fluoxetine	Chronic [daily, NR]	None	Common goldfish	Active avoidance
Fluoxetine	Acute	Acute uncontrollable stress	Rat	Conditioned freezing
Citalopram	Acute	None	Human	Active avoidance
Citalopram	Chronic [daily, 22x]	None	Rat	Conditioned freezing
Citalopram	Acute	None	Human	Fear potentiated startle
Fluoxetine	Acute	None	Rat	Conditioned freezing
Fluoxetine	Acute	None	Rat	Conditioned freezing
Fluoxetine	Acute	None	Rat	Conditioned freezing
Fluoxetine	Chronic [daily, minimally 14x]	None	Rat	Conditioned freezing
Fluoxetine	Acute [1 day]	None	Rat	Conditioned freezing
Fluoxetine	Chronic [daily, minimally 14x]	Acute uncontrollable stress	Rat	Conditioned freezing
Fluoxetine	Chronic [daily, 10x]	Immobilization stressed	Rat	Active avoidance
Paroxetine	Acute	None	Rat	Conditioned freezing
Paroxetine	Acute	None	Rat	Fear potentiated startle
Fluoxetine	Acute	None	Rat	Conditioned freezing
Paroxetine	Chronic [daily, 28x]	None	Mouse	Conditioned freezing
Citalopram	Acute	None	Mouse	Conditioned freezing
Fluoxetine	Acute	None	Rat	Conditioned freezing
Escitalopram	Chronic [daily, 14x]	None	Rat	Conditioned freezing
Citalopram	Acute	None	Rat	Conditioned freezing
Fluoxetine	Chronic [daily, minimally 14x]	Acute uncontrollable stress	Rat	Conditioned freezing
Citalopram	Acute	None	Rat	Conditioned freezing
Fluoxetine	Acute	None	Rat	Conditioned freezing
Sertraline	Chronic [daily, 13x]	Restraint	Rat	Active avoidance

Supplementary figure 7A. Forest plot of acquisition learning to cue with corresponding study characteristics per experiment. The overall effect was negative (favouring SSRIs) and non-significant (SMD (95% CI) -0.10 (-0.55, 0.36) (P=0.68)). VGV 5-HT2CR = valine-glycine-valine isoform of the serotonin 2C receptor, NR = not reported.

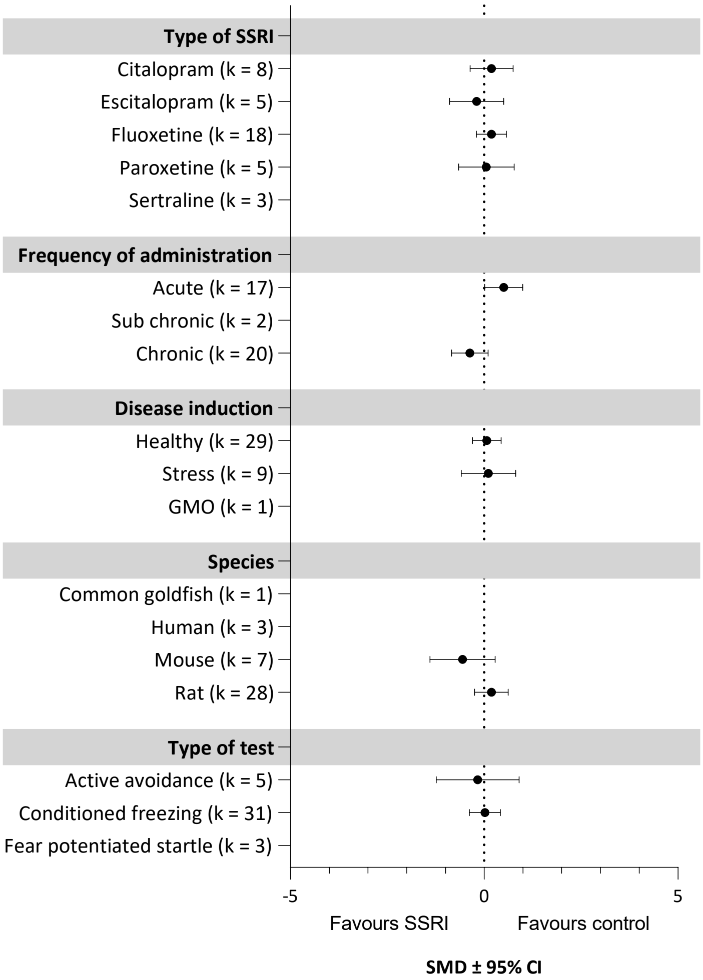

Supplementary figure 7B. Summary of subgroup analyses on the fear learning process acquisition learning to cue. Subgroup analyses were performed on type of SSRI, frequency of administration, disease induction, species, and type of test. Data are shown as standardized mean differences (SMDs) ± 95% confidence interval (CI). Missing values indicate subgroups that were reported, but had insufficient data to include in the subgroup analyses.

Acquisition learning to context

Meta-analysis

A total of 39 experiments (k = 39) were included in the meta-analysis that investigated the effect of SSRIs on the acquisition learning to context. 2 papers comprising of 4 experiments were not included in the meta-analysis since information on the dispersion of the data was missing. Of these 39 experiments, 25 experiments measured acquisition learning to context and 14 experiments acquisition learning measured as expression to context. No effect of SSRIs on acquisition learning to context was observed (SMD (95% CI) -0.24 (-0.60, 0.11); P=0.18; I^2 83%; k = 39) (figure 7C).

None of the three SSRIs that were included in the subgroup analysis revealed a significant effect on acquisition learning to context: citalopram (SMD (95% CI) -0.61 (-1.47, 0.26); P=0.17; I^2 87%; k = 7), escitalopram (SMD (95% CI) -0.07 (-1.09, 0.95); P=0.90; I^2 87%; k = 5) and fluoxetine (SMD (95% CI) 0.02 (-0.47, 0.51); P=0.94; I^2 82%; k = 22) (figure 7D).

Subgroup analysis of frequency of administration showed that both acute (SMD (95% CI) 0.17 (-0.37, 0.71); P=0.54; I^2 85%; k = 16) and subchronic (SMD (95% CI) -0.27 (-1.16, 0.61); P=0.54; I^2 88%; k = 6) SSRI administration had no significant effect on acquisition learning to context. However, chronic SSRI administration significantly reduced fear behaviour as assessed by acquisition learning to context (SMD (95% CI) -0.64 (-1.17, -0.10); P=0.02; I^2 75%; k = 17). Further analysis showed that chronic SSRI administration had a significantly different effect on fear behaviour compared to acute SSRI administration (ΔSMD (95% CI) 0.81 (0.05, 1.57); P=0.046) (figure 7D).

Subgroup analysis of disease induction on the effect of SSRIs on acquisition learning to context showed no significant effect in both healthy subjects (SMD (95% CI) -0.14 (-0.56, 0.28); P=0.51; I^2 82%; k = 29) and stressed subjects (SMD (95% CI) -0.49 (-1.34, 0.35); P=0.25; I^2 89%; k = 8) (figure 7D).

A subgroup analysis of species revealed that SSRIs significantly reduced fear behaviour during acquisition learning to context in mice (SMD (95% CI) -0.92 (-1.53, -0.32); P=0.00; I^2 49%; k = 12) but not in rats (SMD (95% CI) 0.08 (-0.34, 0.51); P=0.70; I^2 85%; k = 26). Additional analysis revealed that SSRI administration had a significantly different effect on fear behaviour in mice than in rats (ΔSMD (95%) 1.01 (0.27, 1.75); P=0.01) (figure 7D).

No significant effect of SSRIs on acquisition learning to context was measured in both the conditioned freezing (SMD (95% CI) -0.37 (-0.81, 0.07); P=0.10; I^2 82%; k = 26) and the passive avoidance test (SMD (95% CI) -0.13 (-1.10, 0.85); P=0.80; I^2 90%; k = 6) in the subgroup analysis of types of fear learning tests (figure 7D).

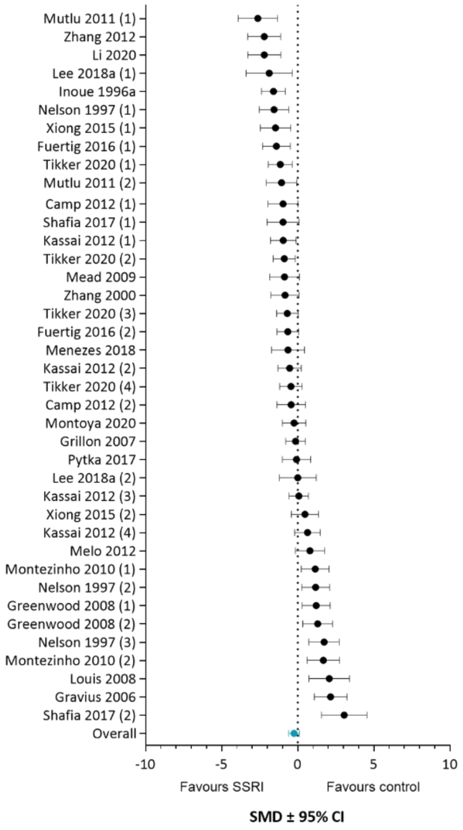

Type of SSRI	Frequency	Disease induction	Species	Type of test
Citalopram	Acute	None	Rat	Passive avoidance
Sertraline	Chronic [daily, 15x]	Single prolonged stress	Mouse	Conditioned freezing
Sertraline	Chronic [daily, 12x]	Single prolonged stress	Mouse	Conditioned freezing
Fluoxetine	Chronic [daily, 14x]	Single prolonged stress	Rat	Conditioned freezing
Citalopram	Acute	None	Rat	Conditioned freezing
Fluoxetine	Subchronic [daily, 5x]	None	Rat	Active avoidance
Fluoxetine	Chronic [daily, 29x]	Maternal separation	Rat	Conditioned freezing
Escitalopram	Subchronic [daily, 3x]	None	Mouse	Conditioned freezing
Fluoxetine	Chronic [daily, 21x]	None	Mouse	Conditioned freezing
Citalopram	Acute	None	Rat	Passive avoidance
Fluoxetine	Chronic [daily, 23x]	None	Mouse	Conditioned freezing
Fluoxetine	Chronic [daily, 30x]	None	Rat	Inhibitory avoidance
Escitalopram	Acute	None	Rat	Conditioned freezing
Fluoxetine	Chronic [daily, 21x]	Zfpm1CKO	Mouse	Conditioned freezing
Citalopram	Subchronic [daily, 2x]	None	Rat	Avoidance conditioning
Fluoxetine	Chronic [daily, 15x]	None	Rat	Conditioned freezing
Fluoxetine	Chronic [daily, 21x]	Zfpm1CKO	Mouse	Conditioned freezing
Escitalopram	Subchronic [daily, 3x]	Chronic social defeat	Mouse	Conditioned freezing
Paroxetine	Chronic [daily, 15x]	None	Rat	Passive avoidance
Paroxetine	Acute	None	Rat	Conditioned freezing
Fluoxetine	Chronic [daily, 21x]	None	Mouse	Conditioned freezing
Fluoxetine	Chronic [daily, 23x]	None	Mouse	Conditioned freezing
Citalopram	Acute	None	Mouse	Conditioned freezing
Citalopram	Acute	None	Human	Fear potentiated startle
Fluoxetine	Acute	None	Mouse	Step through avoidance
Fluoxetine	Chronic [daily, 14x]	Single prolonged stress	Rat	Conditioned freezing
Sertraline	Acute	None	Rat	Conditioned freezing
Fluoxetine	Chronic [daily, 29x]	None	Rat	Conditioned freezing
Fluoxetine	Acute	None	Rat	Conditioned freezing
Fluoxetine	Chronic [daily, 19x]	None	Rat	Plus-maze discriminative avoidance task
Escitalopram	Acute	None	Rat	Conditioned freezing
Fluoxetine	Subchronic [daily, 6x]	None	Rat	Active avoidance
Fluoxetine	Acute	None	Rat	Conditioned freezing
Fluoxetine	Acute	None	Rat	Conditioned freezing
Escitalopram	Acute	None	Rat	Conditioned freezing
Fluoxetine	Subchronic [daily, 5x]	None	Rat	Active avoidance
Fluoxetine	Acute	Olfactory bulbectomized	Rat	Passive avoidance
Citalopram	Acute	None	Rat	Conditioned freezing
Fluoxetine	Chronic [daily, 30x]	Single prolonged stress	Rat	Inhibitory avoidance

Supplementary figure 7C. Forest plot of acquisition learning to context with corresponding study characteristics per experiment. The overall effect was negative (favouring SSRIs) and non-significant (SMD (95% CI) -0.02 (-0.43, 0.38) (P=0.92)). Zfpm1CKO = Zfpm1 conditional knockout.

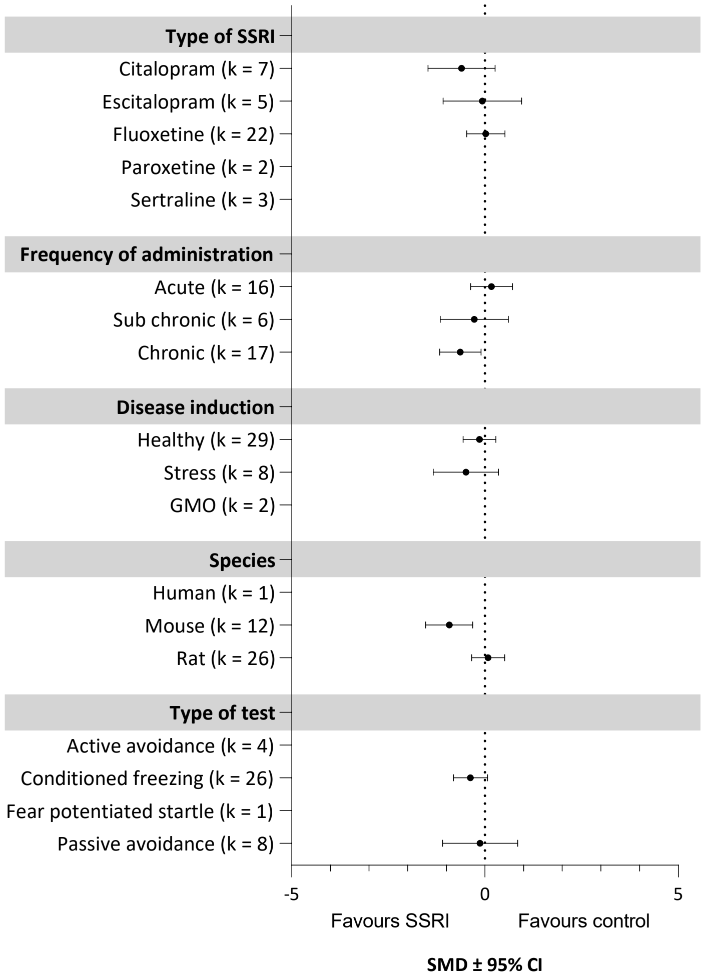

Supplementary figure 7D. Summary of subgroup analyses on the fear learning process acquisition learning to context. Subgroup analyses were performed on type of SSRI, frequency of administration, disease induction, species and type of test. Data are shown as standardized mean differences (SMDs) ± 95% confidence interval (CI). Missing values indicate subgroups that were reported, but had insufficient data to include in the subgroup analyses.

Cued fear expression after acquisition learning

Meta-analysis

A total of 41 experiments (k = 41) were included in the meta-analysis that investigated the effect of SSRIs on cued fear expression after acquisition learning. One article, comprising of one experiment was not included in the meta-analysis since the results were not extractable due to the low quality of figures in the paper, the figures were not readable. No effect of SSRIs on cued fear expression after acquisition learning was observed (SMD (95% CI) -0.13 (-0.40, 0.14); P=0.33; I^2 75%; k = 41) (figure 7E).

Both fluoxetine (SMD (95% CI) -0.27 (-0.64, 0.10); P=0.16; I^2 69%; k = 21) and paroxetine (SMD (95% CI) -0.07 (-0.62, 0.47); P=0.79; I^2 78%; k = 10) were included in the subgroup analysis and did not have a significant effect on cued fear expression after acquisition learning (figure 7F).

Subgroup analysis showed that both acute (SMD (95% CI) 0.48 (0.06, 0.89); P=0.02; I^2 74%; k = 15) and chronic (SMD (95% CI) -0.51 (-0.85, -0.18); P=0.003; I^2 67%; k = 23) SSRI administration had a significant effect on cued fear expression after acquisition learning. The difference in effect of these two subgroups was significant (ΔSMD (95%) 0.99 (0.46, 1.52); P=0.00). Acute SSRI administration led to significantly increased fear behaviour whereas chronic SSRI administration caused fear behaviour to decrease (figure 7F).

Subgroup analysis on the effect of SSRIs on cued fear expression after acquisition learning in healthy subjects showed no significant effect (SMD (95% CI) 0.08 (-0.20, 0.37); P=0.57; I^2 73%; k = 32) but significantly reduced fear behaviour in stressed subjects (SMD (95% CI) -0.97 (-1.59, -0.34); P=0.00; I^2 72%; k = 7). Furthermore, the anxiolytic effect of SSRIs was found to be significantly stronger in stressed subjects than in healthy subjects (ΔSMD (95%) 1.05 (0.37, 1.74); P=0.00) (figure 7F).

A subgroup analysis of species revealed no significant effect of SSRIs on cued fear expression after acquisition learning in rats (SMD (95% CI) 0.18 (-0.22, 0.57); P=0.38; I^2 79%; k = 19) whereas a significant effect was found for mice (SMD (95% CI) -0.48 (-0.87, -0.09); P=0.02; I^2 66%; k = 20). Additional analysis showed that the effect of SSRIs had a significantly different effect in mice compared to rats (ΔSMD (95%) 0.66 (0.10, 1.21); P=0.03) (figure 7F).

SSRIs were shown to have a significant effect on cued fear expression after acquisition learning when measured in the conditioned freezing test, fear behaviour was significantly decreased (SMD (95% CI) -0.40 (-0.70, -0.10); P=0.009; I^2 74%; k = 29). No significant effect of SSRIs on cued fear expression after acquisition learning was found when fear behaviour was measured using the fear potentiated startle test (SMD (95% CI) 0.38 (-0.08, 0.85); P=0.11; I^2 56%; k = 11) (figure 7F). Further analysis showed that the effect of SSRIs measured in tests based on conditioned freezing was significantly different from that measured in fear potentiated startle tests (ΔSMD (95%) 0.79 (0.23, 1.34); P=0.02).

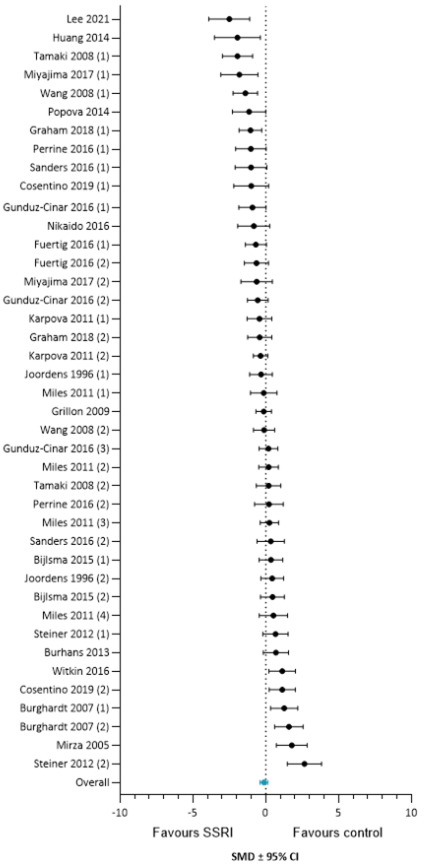

Study	Type of SSRI	Frequency	Disease induction	Species	Type of test
Lee 2021	Fluoxetine	Chronic [daily, 14x]	Single prolonged stress	Rat	Conditioned freezing
Huang 2014	Paroxetine	Chronic [daily, 14x]	None	Mouse	Conditioned freezing
Tamaki 2008 (1)	Fluvoxamine	Chronic [daily, 14x]	WIRS	Mouse	Conditioned freezing
Miyajima 2017 (1)	Fluoxetine	Acute	None	Mouse	Conditioned freezing
Wang 2008 (1)	Paroxetine	Chronic [daily, 14x]	Single prolonged stress	Rat	Conditioned freezing
Popova 2014	Fluoxetine	Chronic [daily, 14x]	None	Mouse	Conditioned freezing
Graham 2018 (1)	Fluoxetine	Chronic [daily, 14x]	High fear	Rat	Conditioned freezing
Perrine 2016 (1)	Paroxetine	Chronic [daily, 7x]	Single prolonged stress	Mouse	Conditioned freezing
Sanders 2016 (1)	Fluoxetine	Chronic [daily, 30x]	None	Mouse	Conditioned freezing
Cosentino 2019 (1)	Fluoxetine	Chronic [daily, 31x]	MeCP2-308	Mouse	Conditioned freezing
Gunduz-Cinar 2016 (1)	Fluoxetine	Chronic [daily, 21x]	None	Mouse	Conditioned freezing
Nikaido 2016	Paroxetine	Chronic [daily, 13x]	None	Rat	Conditioned freezing
Fuertig 2016 (1)	Escitalopram	Subchronic [daily, 4x]	Chronic social defeat	Mouse	Conditioned freezing
Fuertig 2016 (2)	Escitalopram	Subchronic [daily, 4x]	None	Mouse	Conditioned freezing
Miyajima 2017 (2)	Fluoxetine	Acute	Pdcd1-/-	Mouse	Conditioned freezing
Gunduz-Cinar 2016 (2)	Fluoxetine	Chronic [daily, 21x]	None	Mouse	Conditioned freezing
Karpova 2011 (1)	Fluoxetine	Chronic [daily, 14x]	None	Mouse	Conditioned freezing
Graham 2018 (2)	Fluoxetine	Chronic [daily, 14x]	Low fear	Rat	Conditioned freezing
Karpova 2011 (2)	Fluoxetine	Chronic [daily, 23x]	None	Mouse	Conditioned freezing
Joordens 1996 (1)	Fluvoxamine	Acute	None	Rat	Fear potentiated startle
Miles 2011 (1)	Fluoxetine	Acute	None	Rat	Fear potentiated startle
Grillon 2009	Citalopram	Chronic [daily, 14x]	None	Human	Fear potentiated startle
Wang 2008 (2)	Paroxetine	Acute	Single prolonged stress	Rat	Conditioned freezing
Gunduz-Cinar 2016 (3)	Citalopram	Chronic [daily, 21x]	None	Mouse	Conditioned freezing
Miles 2011 (2)	Fluoxetine	Acute	None	Rat	Fear potentiated startle
Tamaki 2008 (2)	Fluvoxamine	Acute	WIRS	Mouse	Conditioned freezing
Perrine 2016 (2)	Paroxetine	Chronic [daily, 7x]	None	Mouse	Conditioned freezing
Miles 2011 (3)	Fluoxetine	Chronic [daily, 21x]	None	Rat	Fear potentiated startle
Sanders 2016 (2)	Fluoxetine	Chronic [daily, 24x]	None	Mouse	Conditioned freezing
Bijlsma 2015 (1)	Paroxetine	Chronic [daily, 21x]	None	Rat	Fear potentiated startle
Joordens 1996 (2)	Fluvoxamine	Acute	None	Rat	Fear potentiated startle
Bijlsma 2015 (2)	Paroxetine	Acute	None	Rat	Fear potentiated startle
Miles 2011 (4)	Fluoxetine	Chronic [daily, 21x]	None	Rat	Fear potentiated startle
Steiner 2012 (1)	Fluoxetine	Acute	None	Rat	Fear potentiated startle
Burhans 2013	Fluoxetine	Subchronic [daily, 5x]	None	Rabbit	Conditioned freezing
Witkin 2016	Paroxetine	Acute	None	Mouse	Conditioned freezing
Cosentino 2019 (2)	Fluoxetine	Chronic [daily, 8x]	None	Mouse	Conditioned freezing
Burghardt 2007 (1)	Fluoxetine	Acute	None	Rat	Conditioned freezing
Burghardt 2007 (2)	Citalopram	Acute	None	Rat	Conditioned freezing
Mirza 2005	Paroxetine	Acute	None	Rat	Conditioned emotional response
Steiner 2012 (2)	Sertraline	Acute	None	Rat	Fear potentiated startle
Overall					

Supplementary figure 7E. Forest plot of cued fear expression after acquisition learning with corresponding study characteristics per experiment. The overall effect was negative (favouring SSRIs) and non-significant (SMD (95% CI) -0.13 (-0.40, 0.14) (P=0.33)). WIRS = water immersion restraint stress, MeCP2-308 = truncated form of methyl-CpG binding protein 2, Pdcd1-/- = PD-1 deficient.

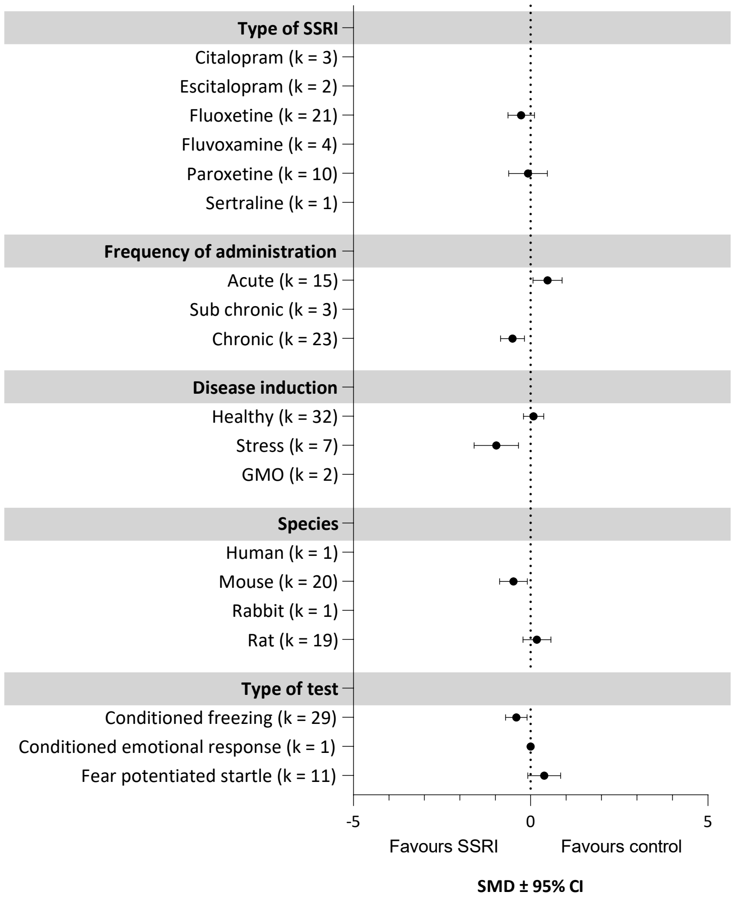

Supplementary figure 7F. Summary of subgroup analyses on the fear learning process cued fear expression after acquisition learning. Subgroup analyses were performed on type of SSRI, frequency of administration, disease induction, species and type of test. Data are shown as standardized mean differences (SMDs) ± 95% confidence interval (CI). Missing values indicate subgroups that were reported, but had insufficient data to include in the subgroup analyses.

Contextual fear expression after acquisition learning

<u>Meta-analysis</u>

A total of 118 experiments (k = 118) were included in the meta-analysis that investigated the effect of SSRIs on contextual fear expression after acquisition learning. SSRIs were found to have a significant anxiolytic effect on this fear learning process (SMD (95% CI) -0.82 (-1.06, -0.59); P=0.00; I^2 85%; k = 118) (figure 7G).

Subgroup analyses showed that most SSRIs were favoured over placebo (citalopram: SMD (95% CI) -0.75 (-1.20, -0.31); P=0.00; I^2 70%; k = 29, fluoxetine: SMD (95% CI) -0.56 (-0.97, -0.14); P=0.01; I^2 84%; k = 33, fluvoxamine: SMD (95% CI) -1.66 (-2.38, -0.94); P=0.00; I^2 87%; k = 12, and sertraline: SMD (95% CI) -1.55 (-2.10, -1.01); P=0.00; I^2 84%; k = 21). Except for escitalopram (SMD (95% CI) 0.34 (-0.42, 1.09); P=0.38; I^2 90%; k = 10) and paroxetine (SMD (95% CI) -0.69 (-1.37, 0.00);

P=0.05; I^2 85%; k = 13), for which the effect on contextual fear expression after acquisition learning did not differ from placebo (figure 7H). Further analyses showed that the anxiolytic effect of sertraline was significantly stronger than that of fluoxetine (ΔSMD (95%) 1.00 (0.31, 1.68); P=0.04).

Subgroup analysis showed that both acute (SMD (95% CI) -0.59 (-0.90, -0.27); P=0.00; I^2 86%; k = 62) and chronic (SMD (95% CI) -1.27 (-1.65, -0.89); P=0.00; I^2 83%; k = 45) SSRI administration significantly reduced fear behaviour as assessed by contextual fear expression after acquisition learning. However, chronic SSRI administration caused a significantly larger reduction in fear behaviour than acute SSRI administration (SMD (95%) 0.68 (0.19, 1.17); P=0.02). Sub chronic treatment of SSRIs did not have a significant effect on contextual fear expression after acquisition learning (SMD (95% CI) -0.45 (-1.19, 0.29); P=0.24; I^2 79%; k = 11) (figure 7H).

SSRI administration was found to cause a significant reduction in fear behaviour in both healthy (SMD (95% CI) -0.66 (-0.91, -0.40); P=0.00; I^2 85%; k = 97) and stressed (SMD (95% CI) -1.68 (-2.30, -1.06); P=0.00; I^2 83%; k = 17) subjects as measured by contextual fear expression after acquisition learning. In addition, the anxiolytic effect of SSRIs was found to be significantly stronger in stressed subjects than in healthy subjects (ΔSMD (95%) 1.02 (0.36, 1.69); P=0.00) (figure 7H).

A subgroup analysis of species revealed that the administration of SSRIs in both mice (SMD (95% CI) -1.07 (-1.53, -0.62); P=0.00; I^2 86%; k = 34) and rats (SMD (95% CI) -0.77 (-1.06, -0.48); P=0.00; I^2 85%; k = 80) significantly reduced fear behaviour as assessed by contextual fear expression after acquisition learning (figure 7H).

Fear behaviour was significantly reduced by SSRIs when measured in the conditioned freezing test as shown by a subgroup analysis of types of fear learning tests (SMD (95% CI) -1.06 (-1.32, -0.80); P=0.00; I^2 85%; k = 93). No significant effect on contextual fear expression after acquisition learning was found when fear behaviour was measured using the fear potentiated startle test (SMD (95% CI) 0.25 (-0.71, 1.20); P=0.61; I^2 74%; k = 6) (figure 7H). Furthermore, comparing the outcome measures of these two types of tests revealed that significantly lower levels of fear behaviour were measured in the conditioned freezing test after SSRI administration as compared to the fear potentiated startle test (ΔSMD (95%) 1.31 (0.32, 2.29); P=0.01).

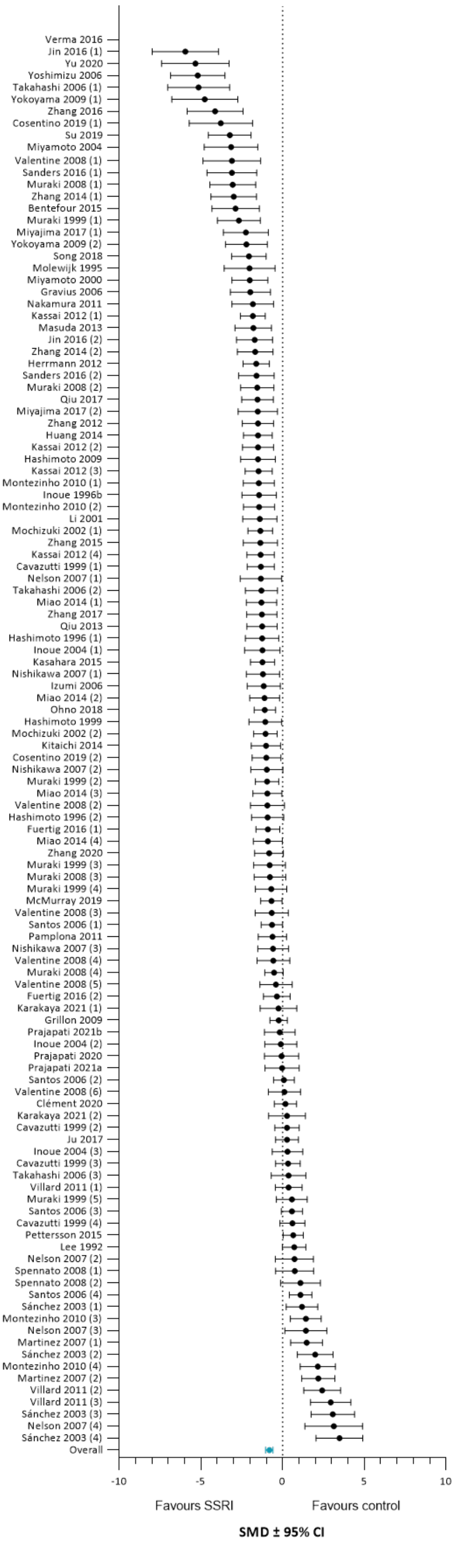

Study	Type of SSRI	Frequency	Disease induction	Species	Type of test
Verma 2016	Sertraline	Chronic [daily, 13x]	None	Mouse	Conditioned freezing
Jin 2016 (1)	Sertraline	Chronic [daily, 14x]	Time-dependent sensitization	Rat	Conditioned freezing
Yu 2020	Fluoxetine	Chronic [daily, 16x]	None	Mouse	Conditioned freezing
Yoshimizu 2006	Fluvoxamine	Acute	None	Rat	Conditioned freezing
Takahashi 2006 (1)	Paroxetine	Chronic [daily, 14x]	Single prolonged stress	Rat	Conditioned freezing
Yokoyama 2009 (1)	Fluvoxamine	Subchronic [daily, 5x]	None	Rat	Conditioned freezing
Zhang 2016	Sertraline	Chronic [daily, 15x]	Time dependent sensitization	Rat	Conditioned freezing
Cosentino 2019 (1)	Fluoxetine	Chronic [daily, 8x]	MeCP2-308	Mouse	Conditioned freezing
Su 2019	Sertraline	Chronic [daily, 7x]	Single prolonged stress	Rat	Conditioned freezing
Miyamoto 2004	Fluovoxamine	Acute	None	Mouse	Conditioned freezing
Valentine 2008 (1)	Fluoxetine	Chronic [daily, 21x]	Chronic unpredictable stress	Rat	Two-way active avoidance
Sanders 2016 (1)	Fluoxetine	Chronic [daily, 30x]	None	Mouse	Conditioned freezing
Muraki 2008 (1)	Citalopram	Acute	None	Rat	Conditioned freezing
Zhang 2014 (1)	Sertraline	Chronic [daily, 15x]	Time dependent sensitization	Mouse	Conditioned freezing
Bentefour 2015	Paroxetine	Chronic [daily, 7x]	None	Mouse	Conditioned freezing
Muraki 1999 (1)	Citalopram	Acute	None	Rat	Conditioned freezing
Miyajima 2017 (1)	Fluoxetine	Acute	Pdcd1-/-	Mouse	Conditioned freezing
Yokoyama 2009 (2)	Fluvoxamine	Acute	None	Rat	Conditioned freezing
Song 2018	Sertraline	Chronic [daily, 10x]	Single prolonged stress	Rat	Conditioned freezing
Molewijk 1995	Fluvoxamine	Acute	None	Rat	Conditioned freezing
Miyamoto 2000	Fluvoxamine	Acute	None	Mouse	Conditioned freezing
Gravius 2006	Citalopram	Acute	None	Rat	Conditioned freezing
Nakamura 2011	Fluoxetine	Acute	None	Mouse	Conditioned freezing
Kassai 2012 (1)	Sertraline	Acute	None	Rat	Conditioned freezing
Masuda 2013	Citalopram	Acute	None	Rat	Conditioned freezing
Jin 2016 (2)	Sertraline	Chronic [daily, 14x]	None	Mouse	Conditioned freezing
Zhang 2014 (2)	Sertraline	Chronic [daily, 15x]	None	Mouse	Conditioned freezing
Herrmann 2012	Fluoxetine	Chronic [daily, 28x]	None	Mouse	Conditioned freezing
Sanders 2016 (2)	Fluoxetine	Chronic [daily, 24x]	None	Mouse	Conditioned freezing
Muraki 2008 (2)	Citalopram	Acute	None	Rat	Conditioned freezing
Qiu 2017	Sertraline	Chronic [daily, 8x]	None	Rat	Conditioned freezing
Miyajima 2017 (2)	Fluoxetine	Acute	None	Mouse	Conditioned freezing
Zhang 2012	Sertraline	Chronic [daily, 15x]	None	Mouse	Conditioned freezing
Huang 2014	Paroxetine	Chronic [daily, 14x]	None	Mouse	Conditioned freezing
Kassai 2012 (2)	Fluoxetine	Acute	None	Rat	Conditioned freezing
Hashimoto 2009	Citalopram	Acute	None	Rat	Conditioned freezing
Kassai 2012 (3)	Paroxetine	Acute	None	Rat	Conditioned freezing
Montezinho 2010 (1)	Escitalopram	Acute	None	Rat	Conditioned freezing
Inoue 1996b	Citalopram	Acute	None	Rat	Conditioned freezing
Montezinho 2010 (2)	Escitalopram	Acute	None	Rat	Conditioned freezing
Li 2001	Fluvoxamine	Acute	None	Rat	Conditioned freezing
Mochizuki 2002 (1)	Fluvoxamine	Acute	None	Rat	Conditioned freezing
Zhang 2015	Sertraline	Chronic [daily, 15x]	None	Mouse	Conditioned freezing
Kassai 2012 (4)	Escitalopram	Acute	None	Rat	Conditioned freezing
Cavazutti 1999 (1)	Fluoxetine	Chronic [daily, 26x]	None	Rat	Conditioned emotional response
Nelson 2007 (1)	Paroxetine	Chronic [daily, ~150x]	3xTgAD	Mouse	Passive avoidance
Takahashi 2006 (2)	Paroxetine	Chronic [daily, 14x]	None	Rat	Conditioned freezing
Miao 2014 (1)	Sertraline	Chronic [daily, 18x]	Single prolonged stress	Rat	Conditioned freezing
Zhang 2017	Sertraline	Subchronic [daily, 5x]	None	Mouse	Conditioned freezing
Qiu 2013	Sertraline	Chronic [daily, 15x]	None	Mouse	Conditioned freezing
Hashimoto 1996 (1)	Citalopram	Acute	None	Rat	Conditioned freezing
Inoue 2004 (1)	Citalopram	Acute	None	Rat	Conditioned freezing
Kasahara 2015	Citalopram	Acute	None	Rat	Conditioned freezing
Nishikawa 2007 (1)	Paroxetine	Acute	None	Rat	Conditioned freezing
Izumi 2006	Citalopram	Acute	None	Rat	Conditioned freezing
Miao 2014 (2)	Sertraline	Chronic [daily, 18x]	Single prolonged stress	Rat	Conditioned freezing
Ohno 2018	Fluvoxamine	Acute	None	Mouse	Conditioned freezing
Hashimoto 1999	Citalopram	Acute	None	Rat	Conditioned freezing
Mochizuki 2002 (2)	Fluoxetine	Acute	None	Rat	Conditioned freezing
Kitaichi 2014	Citalopram	Acute	None	Rat	Conditioned freezing
Cosentino 2019 (2)	Fluoxetine	Chronic [daily, 8x]	None	Mouse	Conditioned freezing
Nishikawa 2007 (2)	Citalopram	Acute	None	Rat	Conditioned freezing
Muraki 1999 (2)	Citalopram	Acute	None	Rat	Conditioned freezing
Miao 2014 (3)	Sertraline	Chronic [daily, 18x]	Single prolonged stress	Rat	Conditioned freezing
Valentine 2008 (2)	Fluoxetine	Chronic [daily, 21x]	None	Rat	Two-way active avoidance
Hashimoto 1996 (2)	Fluvoxamine	Acute	None	Rat	Conditioned freezing
Fuertig 2016 (1)	Escitalopram	Subchronic [daily, 4x]	Chronic social defeat	Mouse	Conditioned freezing
Miao 2014 (4)	Sertraline	Chronic [daily, 18x]	Single prolonged stress	Rat	Conditioned freezing
Zhang 2020	Sertraline	Chronic [daily, 7x]	Time dependent sensitization	Rat	Conditioned freezing
Muraki 1999 (3)	Citalopram	Acute	None	Rat	Conditioned freezing
Muraki 2008 (3)	Citalopram	Acute	None	Rat	Conditioned freezing
Muraki 1999 (4)	Citalopram	Acute	None	Rat	Conditioned freezing
McMurray 2019	Fluoxetine	Chronic [daily, 31x]	None	Mouse	Conditioned freezing
Valentine 2008 (3)	Fluoxetine	Subchronic [daily, 6x]	None	Rat	Two-way active avoidance
Santos 2006 (1)	Fluoxetine	Acute	None	Rat	Fear potentiated startle
Pamplona 2011	Fluoxetine	Chronic [daily, 25x]	None	Mouse	CODA
Nishikawa 2007 (3)	Fluvoxamine	Acute	None	Rat	Conditioned freezing
Valentine 2008 (4)	Fluoxetine	Chronic [daily, 20x]	None	Rat	Two-way active avoidance
Muraki 2008 (4)	Citalopram	Acute	None	Rat	Conditioned freezing
Valentine 2008 (5)	Citalopram	Subchronic [daily, 6x]	None	Rat	Two-way active avoidance
Fuertig 2016 (2)	Escitalopram	Subchronic [daily, 4x]	None	Mouse	Conditioned freezing
Karakaya 2021 (1)	Citalopram	Acute	None	Zebrafish	Conditioned place aversion
Grillon 2009	Citalopram	Chronic [daily, 14x]	None	Human	Fear potentiated startle
Prajapati 2021b	Setraline	Chronic [daily, 25x]	Variable stress	Rat	Conditioned freezing
Inoue 2004 (2)	Citalopram	Acute	None	Rat	Conditioned freezing
Prajapati 2020	Paroxetine	Subchronic [daily, 6x]	Variable stress	Rat	Conditioned freezing
Prajapati 2021a	Paroxetine	Chronic [daily, 25x]	Variable stress	Rat	Conditioned freezing
Santos 2006 (2)	Fluoxetine	Subchronic [3x in 24 h]	None	Rat	Fear potentiated startle
Valentine 2008 (6)	Fluoxetine	Chronic [daily, 21x]	None	Rat	Two-way active avoidance
Clément 2020	Citalopram	Acute	None	Zebrafish	Conditioned place aversion
Karakaya 2021 (2)	Citalopram	Acute	None	Zebrafish	Conditioned place aversion
Cavazutti 1999 (2)	Fluoxetine	Chronic [daily, 12x]	None	Rat	Conditioned emotional response
Ju 2017	Fluoxetine	Chronic [daily, 16x]	None	Mouse	Conditioned freezing
Inoue 2004 (3)	Citalopram	Acute	None	Rat	Conditioned freezing
Cavazutti 1999 (3)	Fluoxetine	Subchronic [daily, 5x]	None	Rat	Conditioned emotional response
Takahashi 2006 (3)	Paroxetine	Subchronic [daily, 2x]	Single prolonged stress	Rat	Conditioned freezing
Villard 2011 (1)	Fluoxetine	Acute	None	Mouse	Conditioned freezing
Muraki 1999 (5)	Citalopram	Acute	None	Rat	Conditioned freezing
Santos 2006 (3)	Fluoxetine	Acute	None	Rat	Fear potentiated startle
Cavazutti 1999 (4)	Fluoxetine	Acute	None	Rat	Conditioned emotional response
Pettersson 2015	Escitalopram	Acute	None	Rat	Fear potentiated startle
Lee 1992	Fluoxetine	Acute	None	Rat	Inhibitory avoidance
Nelson 2007 (2)	Paroxetine	Chronic [daily, ~150x]	3xTgAD	Mouse	Passive avoidance
Spennato 2008 (1)	Fluoxetine	Chronic [daily, 21x]	None	Rat	Conditioned freezing
Spennato 2008 (2)	Fluoxetine	Chronic [daily, 21x]	None	Rat	Conditioned freezing
Santos 2006 (4)	Fluoxetine	Subchronic [3x in 24 h]	None	Rat	Fear potentiated startle
Sánchez 2003 (1)	Citalopram	Acute	None	Rat	Conditioned freezing
Montezinho 2010 (3)	Escitalopram	Acute	None	Rat	Conditioned freezing
Nelson 2007 (3)	Paroxetine	Chronic [daily, ~150x]	None	Mouse	Passive avoidance
Martinez 2007 (1)	Fluoxetine	Acute	None	Rat	Conditioned freezing
Sánchez 2003 (2)	Citalopram	Acute	None	Rat	Conditioned freezing
Montezinho 2010 (4)	Escitalopram	Acute	None	Rat	Conditioned freezing
Martinez 2007 (2)	Fluoxetine	Acute	None	Rat	Conditioned freezing
Villard 2011 (2)	Fluvoxamine	Acute	None	Mouse	Conditioned freezing
Villard 2011 (3)	Sertraline	Acute	None	Mouse	Conditioned freezing
Sánchez 2003 (3)	Escitalopram	Acute	None	Rat	Conditioned freezing
Nelson 2007 (4)	Paroxetine	Chronic [daily, ~150x]	None	Mouse	Passive avoidance
Sánchez 2003 (4)	Escitalopram	Acute	None	Rat	Conditioned freezing

Supplementary figure 7G. Forest plot of contextual fear expression after acquisition learning with corresponding study characteristics per experiment. The overall effect was negative (favouring SSRIs) and significant (SMD (95% CI) -0.82 (-1.05, -0.58) (P=0.00)). MeCP2-308 = truncated form of methyl-CpG binding protein 2, Pdcd1-/- = PD-1 deficient, 3xTgAD = triple transgenic Alzheimer's disease, CODA = conditioned odor avoidance task.

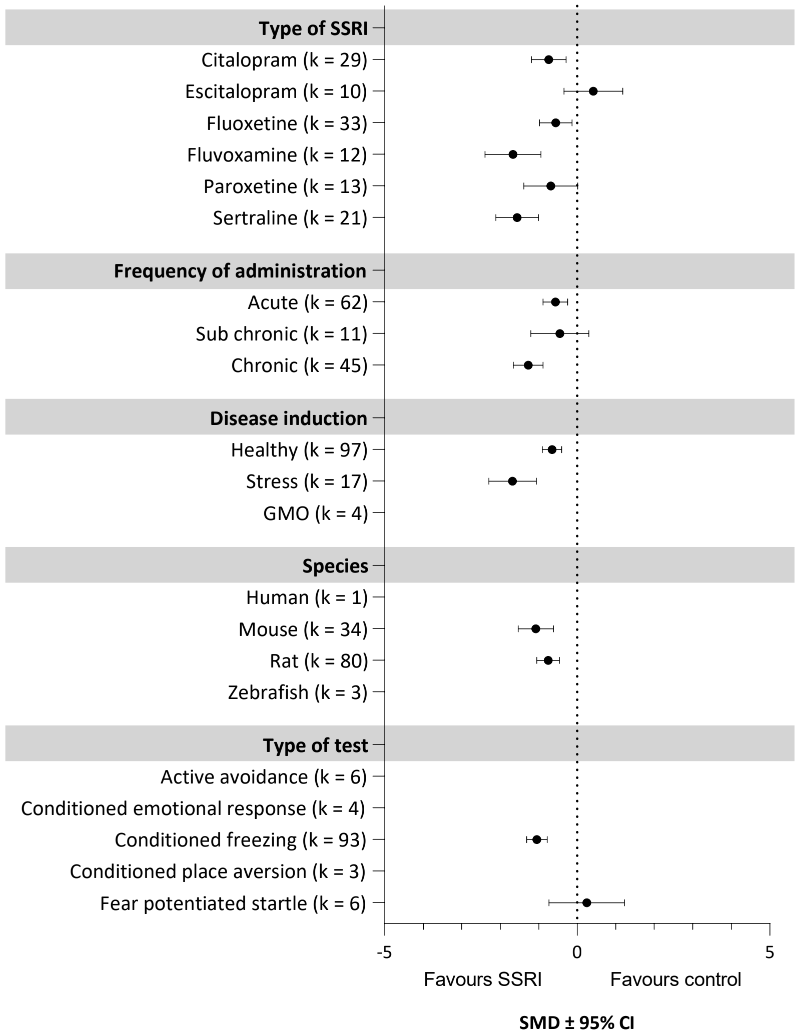

Supplementary figure 7H. Summary of subgroup analyses on the fear learning process contextual fear expression after acquisition learning. Subgroup analyses were performed on type of SSRI, frequency of administration, disease induction, species and type of test. Data are shown as standardized mean differences (SMDs) ± 95% confidence interval (CI). Missing values indicate subgroups that were reported, but had insufficient data to include in the subgroup analyses.

Extinction learning to cue

Meta-analysis

A total of 17 experiments (k = 17) were included in the meta-analysis that investigated the effect of SSRIs on the extinction learning to cue. Of these 17 experiments, 14 experiments measured extinction learning to cue and 3 experiments extinction learning measured as fear expression to cue. SSRIs were observed to have a significant anxiolytic effect on extinction learning to cue (SMD (95% CI) -0.96 (-1.60, -0.32); P=0.00; I^2 88%; k = 17) (figure 7J).

The only SSRI that was included in the subgroup analysis on type of SSRI was paroxetine and showed a significant effect on extinction learning to cue in the subgroup analysis, fear behaviour was significantly reduced after administration (SMD (95% CI) -1.53 (-2.36, -0.70); P=0.00; I^2 85%; k = 9) (figure 7K).

Subgroup analysis showed that acute SSRI administration significantly reduced fear behaviour as measured during extinction learning to cue (SMD (95% CI) -2.14 (-3.16, -1.11); P=0.00; I^2 87%; k = 6). However, chronic SSRI administration did not have a significant effect on extinction learning to cue (SMD (95% CI) -0.14 (-0.98, 0.70); P=0.74; I^2 85%; k = 8) (figure 7K).

Subgroup analysis of disease induction on the effect of SSRIs on extinction learning to cue in healthy subjects showed no significant effect on fear behaviour (SMD (95% CI) -0.40 (-0.97, 0.17); P=0.17; I^2 83%; k = 13) (figure 7K).

A subgroup analysis of species revealed that SSRIs significantly reduced fear behaviour as assessed during extinction learning to cue in mice (SMD (95% CI) -1.47 (-2.41, -0.53); P=0.00; I^2 90%; k = 9) whereas no significant effect was found for rats (SMD (95% CI) -0.43 (-1.48, 0.61); P=0.42; I^2 86%; k = 7) (figure 7K).

A subgroup analysis of types of fear learning tests showed that SSRIs significantly reduced fear behaviour measured during extinction learning to cue in the conditioned freezing test (SMD (95% CI) -0.97 (-1.69, -0.25); P=0.01; I^2 89%; k = 15) (figure 7K).

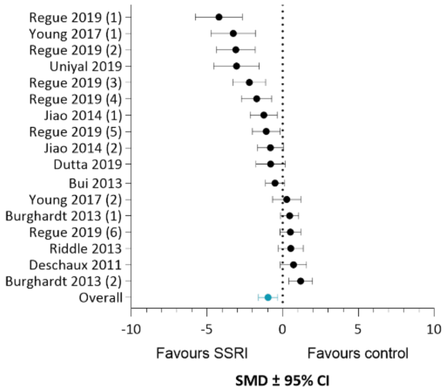

Type of SSRI	Frequency	Disease induction	Species	Type of test
Paroxetine	Acute	VGV 5-HT2CR	Mouse	Conditioned freezing
Citalopram	Acute	None	Mouse	Conditioned freezing
Paroxetine	Acute	VGV 5-HT2CR	Mouse	Conditioned freezing
Sertraline	Chronic [daily, 11x]	Single prolonged stress	Rat	Conditioned freezing
Paroxetine	Acute	None	Mouse	Conditioned freezing
Paroxetine	Chronic [daily, 28x]	VGV 5-HT2CR	Mouse	Conditioned freezing
Paroxetine	Subchronic [daily, 6x]	None	Rat	Active avoidance
Paroxetine	Acute	None	Mouse	Conditioned freezing
Paroxetine	Subchronic [daily, 6x]	None	Rat	Active avoidance
Paroxetine	Subchronic [daily, 2x]	None	Rat	Conditioned freezing
Escitalopram	Chronic [daily, 14x]	None	Human	Conditioned freezing
Citalopram	Acute	None	Mouse	Conditioned freezing
Citalopram	Chronic [daily, 9x]	None	Rat	Conditioned freezing
Paroxetine	Chronic [daily, 28x]	None	Mouse	Conditioned freezing
Fluoxetine	Chronic [daily, 23x]	None	Mouse	Conditioned freezing
Fluoxetine	Chronic [daily, 16x]	None	Rat	Conditioned freezing
Citalopram	Chronic [daily, 22x]	None	Rat	Conditioned freezing

Supplementary figure 7J. Forest plot of extinction learning to cue with corresponding study characteristics per experiment. The overall effect was negative (favouring SSRIs) and significant (SMD (95% CI) -0.90 (-1.61, -0.20) (P=0.01)). VGV 5-HT2CR = valine-glycine-valine isoform of the serotonin 2C receptor.

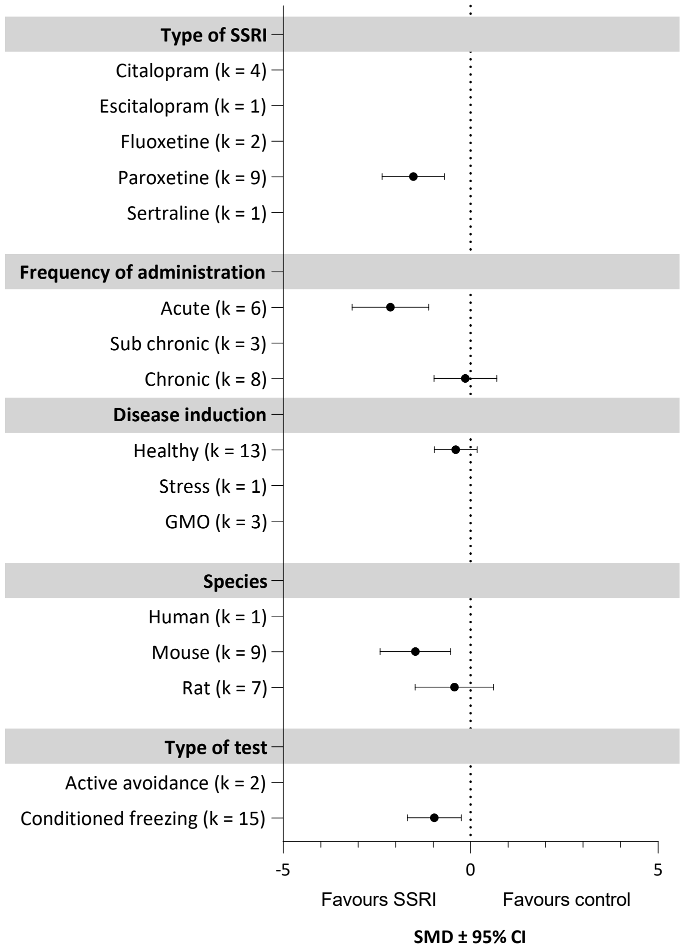

Supplementary figure 7K. Summary of subgroup analyses on the fear learning process extinction learning to cue. Subgroup analyses were performed on type of SSRI, frequency of administration, disease induction, species and type of test. Data are shown as standardized mean differences (SMDs) ± 95% confidence interval (CI). Missing values indicate subgroups that were reported, but had insufficient data to include in the subgroup analyses.

Extinction learning to context

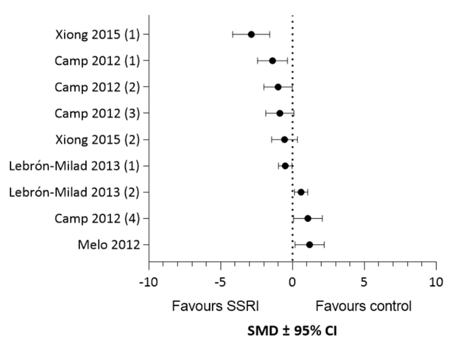

	Type of SSRI	Frequency	Disease induction	Species	Type of test
Xiong 2015 (1)	Fluoxetine	Chronic [daily, 33x]	Maternal separation	Rat	Conditioned freezing
Camp 2012 (1)	Fluoxetine	Chronic [daily, 23x]	None	Mouse	Conditioned freezing
Camp 2012 (2)	Fluoxetine	Chronic [daily, 23x]	None	Mouse	Conditioned freezing
Camp 2012 (3)	Fluoxetine	Chronic [daily, 23x]	None	Mouse	Conditioned freezing
Xiong 2015 (2)	Fluoxetine	Chronic [daily, 31x]	None	Rat	Conditioned freezing
Lebrón-Milad 2013 (1)	Fluoxetine	Chronic [daily, 14x]	None	Rat	Conditioned freezing
Lebrón-Milad 2013 (2)	Fluoxetine	Acute	None	Rat	Conditioned freezing
Camp 2012 (4)	Fluoxetine	Chronic [daily, 23x]	None	Mouse	Conditioned freezing
Melo 2012	Fluoxetine	Chronic [daily, 19x]	None	Rat	Plus-maze discriminative avoidance task

Supplementary figure 7L. Forest plot of extinction learning to context with corresponding study characteristics per experiment.

Cued fear expression after extinction learning

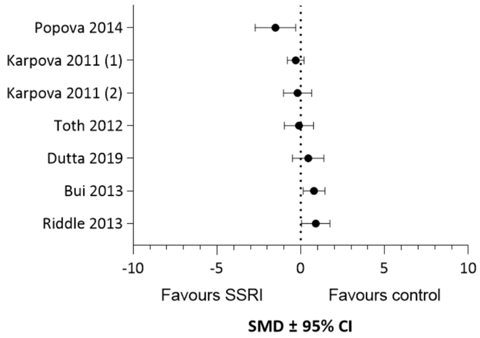

Type of SSRI	Frequency	Disease induction	Species	Type of test
Fluoxetine	Chronic [daily, 16x]	None	Mouse	Conditioned freezing
Fluoxetine	Chronic [daily, 25x]	None	Mouse	Conditioned freezing
Fluoxetine	Chronic [daily, 16x]	None	Mouse	Conditioned freezing
Fluoxetine	Acute	None	Rat	Conditioned freezing
Paroxetine	Subchronic [daily, 3x]	None	Rat	Conditioned freezing
Escitalopram	Chronic [daily, 14x]	None	Human	Conditioned freezing
Fluoxetine	Chronic [daily, 24x]	None	Mouse	Conditioned freezing

Supplementary figure 7M. Forest plot of cued fear expression after extinction learning with corresponding study characteristics per experiment.

Contextual fear expression after extinction learning

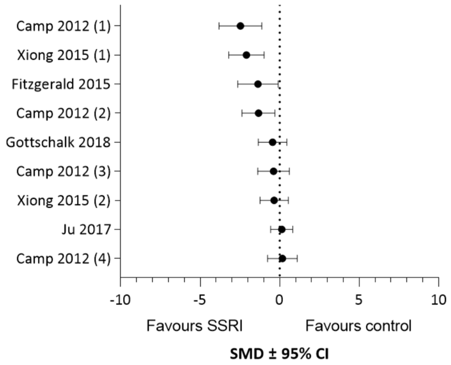

Type of SSRI	Frequency	Disease induction	Species	Type of test
Fluoxetine	Chronic [daily, 23x]	None	Mouse	Conditioned freezing
Fluoxetine	Chronic [daily, 44x]	Maternal separation	Rat	Conditioned freezing
Fluoxetine	Chronic [daily, 22x]	None	Mouse	Conditioned freezing
Fluoxetine	Chronic [daily, 23x]	None	Mouse	Conditioned freezing
Fluoxetine	Chronic [daily, 29x]	None	Rat	Conditioned freezing
Fluoxetine	Chronic [daily, 23x]	None	Mouse	Conditioned freezing
Fluoxetine	Chronic [daily, 44x]	None	Rat	Conditioned freezing
Fluoxetine	Chronic [daily, 16x]	None	Mouse	Conditioned freezing
Fluoxetine	Chronic [daily, 23x]	None	Mouse	Conditioned freezing

Supplementary figure 7N. Forest plot of contextual fear expression after extinction learning with corresponding study characteristics per experiment.

Summary of meta-analysis data

Findings in this systematic review suggest that acute and chronic treatment with SSRIs during acquisition learning to cue may have opposing effects. The cued fear response was significantly enhanced following acute SSRI treatment, whereas chronic SSRI treatment tended to decrease the fear response during acquisition.

Results of this systematic review indicate that chronic SSRI treatment during acquisition learning may reduce the contextual fear response, whereas no effects were found with shorter

treatment duration. Furthermore, the effect of SSRIs on contextual fear learning may well depend on the species in which the SSRIs are tested. SSRIs significantly reduced fear learning in mice whereas no effects of SSRIs were found in rats. Currently available data do not indicate that the effects of SSRIs on contextual fear acquisition in naive animals differ from those in stressed animals.

No beneficial effect of SSRIs on cued fear expression after acquisition learning was indicated by the findings of this systematic review. Findings suggest that the effects of SSRIS are moderated by three factors. First of all, acute treatment had a significant anxiogenic effect whereas chronic treatment had a significant anxiolytic effect. Secondly, SSRIs significantly reduced fear expression in mice while no effect of SSRIs was found in rats. Lastly, SSRIs had a significant anxiolytic effect when measured in the conditioned freezing test, whereas no effect was found in the fear potentiated startle test.

Findings in this systematic review indicate that SSRIs have a robust beneficial effect on contextual fear expression after acquisition learning. The effect was not dependent on duration of treatment, was found in both healthy and stressed subjects, and in both mice and rats. SSRIs had a strong effect on contextual anxiety when measured as conditioned freezing, whereas the currently available data do not suggest an effect on fear potentiated startle. Interestingly, four SSRIs (citalopram, fluoxetine, fluvoxamine and sertraline) had an anxiolytic effect whereas the other two SSRIs (escitalopram and paroxetine) did not have an effect on contextual fear expression. These last two SSRIs (n = 5 and n = 8, respectively) were the least investigated SSRIs and therefore probably did not substantially affect the general effect of SSRIs on contextual fear expression after acquisition learning.

The papers included in this systematic review indicate that SSRIs have a beneficial effect on extinction learning to cue. However, this was only the case for acutely administered SSRIs, chronic SSRI treatment did not have an effect on extinction learning to cue. Also, SSRIs were able to significantly reduce extinction learning in mice while no effect of SSRIs was found in rats.

Sensitivity analysis

Multiple sensitivity analyses were performed per fear learning process in order to check the robustness of the meta-analysis results. Exclusion of the articles by the characteristics described in the method section did not alter the significance or direction of the effect of SSRIs on acquisition learning to cue, acquisition learning to context and cued and contextual fear expression after acquisition learning. However, exclusion of the articles that gave the SSRI during both the measured fear learning process and one or more previous process(es) of fear learning (during acquisition and extinction);

altered the significance but not the direction of the effect of SSRIs on extinction learning to cue. The overall effect of SSRIs on extinction learning to cue was no longer significant after performing the sensitivity analysis.
