# Supplementary material for: The effect of SSRIs on fear learning: a systematic review and meta-analysis
Source: Psychopharmacology (Berl). 2023 Feb 27;240(11):2335–59. doi: 10.1007/s00213-023-06333-7 (PMC10593621; doi:10.1007/s00213-023-06333-7)
Supplement: Supplementary file 5 — Supplementary file5 (HTML 8777 KB) [file 213_2023_6333_MOESM5_ESM.html]

Meta-analysis fear learning


# Meta-analysis fear learning

#### Caspar J. Van Lissa, for Elise Heesbeen & Lucianne Groenink

#### 14 March, 2022

```
## v  Loading original data from 'dat.csv'.
```

**This is a sensitivity analysis; the study {{sens\_study}} has been removed from the data.**


## 0.1 Descriptive statistics

The effect size estimates ranged from -7.45 to 5.77 (\(M = -0.55, SD = 1.57\)).
One study had an effect size estimate almost 15 standard deviations from the mean effect size (\(Z = 14.98\)); it was removed as an outlier.
Sample sizes ranged from 8 to 75 animals (\(M = 21.23, SD = 9.93\)).

## 0.2 Meta-analysis

Meta-analysis was conducted in R (R Core Team 2021) using the R-packages `metafor` (Viechtbauer et al. 2010), and `pema` (Van Lissa and Van Erp 2021).
To estimate overall effects, we used random-effects meta-analysis (Hedges and Vevea 1998).
Separate meta-analyses were conducted for each of the fear learning processes.
The overall pooled effect sizes were:

The overall effect size estimate differed significantly from zero only for Acq retr to ctx, Ext learn to cue, Ext retr to ctx.

The residual heterogeneity \(\tau^2\) was significant for all processes.
This indicates that there was substantial heterogeneity between effect sizes across studies.

## 0.3 Forest plots

The forest plots for the aforementioned meta-analyses are presented below.
Within each plot, studies are ranked by their sampling variance \(vi\);
thus, the most precise estimates are at the bottom, near the overall effect.

Figure 0.1: Forest plot for Acq learn to cue

Figure 0.2: Forest plot for Acq learn to ctx

Figure 0.3: Forest plot for Acq retr to cue

Figure 0.4: Forest plot for Acq retr to ctx

Figure 0.5: Forest plot for Ext learn to cue

Figure 0.6: Forest plot for Ext learn to ctx

Figure 0.7: Forest plot for Ext retr to cue

Figure 0.8: Forest plot for Ext retr to ctx

## 0.4 Moderator analyses

Five categorical moderators were coded: (SSRI, frequency, disease induction, species, type of test).
The effect of these moderators was investigated using meta-regression.
Note however that 33 dummy variables were required to encode all conditions of these categorical moderators.
As the number of moderators was approximately equal to the number of available effect sizes (per process),
these models were not identified.
Addressing this problem requires performing variable selection.
Three steps were taken to do so.
First, variables and categories that did not occur within one subset of the data were omitted.
Secondly, some dummy variables were redundant because some studies had identical values on multiple dummy variables.
Only one of these redundant dummy variables was retained, and its name was updated to reflect all redundant dummies it represents.
For example, in the “Acq learn to ctx” sample, all human studies used the Fear potentiated startle test; no other studies used this test.
Therefore, these two variables were identical and their effects could not be distinguished.
Thus, the analysis shows their joint effect as an effect of `specieshuman;testFPS`.
Thirdly, despite these measures, many meta-regression models dropped all or some of the predictors,
or failed to converge entirely, suggesting the models were empirically non-identified.
Although these models are reported below,
we advise against their substantive interpretation.

The problems with meta-regression can be overcome by a technique that performs variable selection during analysis.
Such a technique was recently developed: Bayesian penalized meta-regression (BRMA), as implemented in the `pema` R-package (Van Lissa and Van Erp (2021)).
By imposing a regularizing (horseshoe) prior on the regression coefficients,
BRMA shrinks all coefficients towards zero, which aids empirical model identification.
Coefficients must overwhelm the prior in order to become significantly different from zero.
Thus, this method also performs variable selection: identifying which moderators are important in predicting the effect size.
The resulting regression coefficients are negatively biased by design, but the estimate of residual heterogeneity \(\tau^2\) is unbiased.
Note that, as this is a Bayesian model, inference is based on credible intervals.
A credible interval is interpreted as follows: The population value falls within this interval with 95% probability (certainty).
This is different from the interpretation of frequentist confidence intervals, which are interpreted as follows: In the long run, 95% of confidence intervals contain the population value.

To examine the effect of a categorical variable,
a reference category must be chosen.
Dummy variables encode the difference between each remaining category and this reference category.
When examining the results,
the intercept represents the expected effect size for a study that falls within the reference category for all categorical variables.
The effect of dummy variables represents the difference of that category with the reference category.
If a dummy variable has a significant effect, that means that that group’s mean differs significantly from the reference category’s mean (i.e., from the intercept).
The analyses below used the following reference categories:

```
## [1] "Acq learn to cue"
## [1] "Acq learn to ctx"
## [1] "Acq retr to cue"
## [1] "Acq retr to ctx"
## [1] "Ext learn to cue"
## [1] "Ext learn to ctx"
## [1] "Ext retr to cue"
## [1] "Ext retr to ctx"
```

### 0.4.1 Classic meta-regression

Note that analyses containing VIF values greater than 5 should be regarded as problematic, due to multicolinearity.
This applies to nearly all models.

### 0.4.2 Bayesian regularized meta-regression:

## 0.5 Subgroup plots

# 1 Conclusion

Meta-analyses revealed limited evidence that overall effects are non-zero in the population,
except for the processes .
All processes showed significant residual heterogeneity.

To explore whether this heterogeneity could be explained by between-study differences,
we conducted meta-regression analysis.
Classic meta-regression analyses were empirically under-identified, however,
because of the high number of moderators and high multicolinearity among them.
BRMA analyses were used, which are robust to multicolinearity, and perform variable selection by shrinking regression coefficients towards zero.
These BRMA analyses revealed no consistent evidence of any significant moderator effect across processes.
To exclude the possibility that multiple dummies interacted together,
I conducted additional sensitivity analyses using `metaCART` - a decision-tree based algorithm that inherently accommodates interactions between dummies.
The `metaCART` analyses also did not find evidence of interaction effects.

Hedges, Larry V., and Jack L. Vevea. 1998. “Fixed- and Random-effects Models in Meta-analysis.” *Psychological Methods* 3 (4): 486–504.

R Core Team. 2021. *R: A Language and Environment for Statistical Computing*. Vienna, Austria: R Foundation for Statistical Computing. https://www.R-project.org/.

Van Lissa, C. J., and S. Van Erp. 2021. “Select Relevant Moderators Using Bayesian Regularized Meta-Regression.” *PsyArxiv*. https://doi.org/10.31234/osf.io/6phs5.

Viechtbauer, Wolfgang et al. 2010. “Conducting Meta-Analyses in R with the Metafor Package.” *J Stat Softw* 36 (3): 1–48.
