# Supplementary material for: The effect of SSRIs on fear learning: a systematic review and meta-analysis
Source: Psychopharmacology (Berl). 2023 Feb 27;240(11):2335–59. doi: 10.1007/s00213-023-06333-7 (PMC10593621; doi:10.1007/s00213-023-06333-7)
Supplement: Supplementary file 6 — Supplementary file6 (PDF 428 KB) [file 213_2023_6333_MOESM6_ESM.pdf]

The effect of SSRIs on fear learning: a systematic review and meta-analysis

Psychopharmacology

Elise J Heesbeen, Elisabeth Y Bijlsma, P Monika Verdouw, Caspar van Lissa, Carlijn Hooijmans, Lucianne Groenink

Corresponding author: Lucianne Groenink, l.groenink@uu.nl

Supplementary file S6. Forest plots of fear learning processes not included in meta-analysis

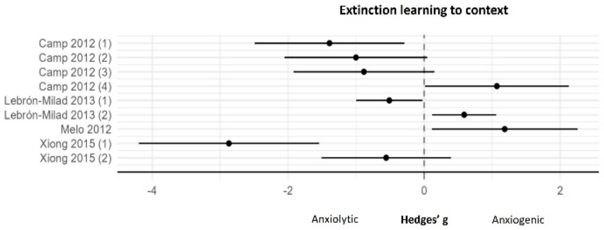

Study	Type of SSRI	Dose	Frequency	Disease induction	Species	Type of test
Camp 2012 (1)	Fluoxetine	10 mg/kg	Chronic [daily, 23x]	None	Mouse	Conditioned freezing
Camp 2012 (2)	Fluoxetine	10 mg/kg	Chronic [daily, 23x]	None	Mouse	Conditioned freezing
Camp 2012 (3)	Fluoxetine	10 mg/kg	Chronic [daily, 23x]	None	Mouse	Conditioned freezing
Camp 2012 (4)	Fluoxetine	10 mg/kg	Chronic [daily, 23x]	None	Mouse	Conditioned freezing
Lebrón-Milad 2013 (1)	Fluoxetine	12 mg/kg	Chronic [daily, 14x]	None	Rat	Conditioned freezing
Lebrón-Milad 2013 (2)	Fluoxetine	10 mg/kg	Acute	None	Rat	Conditioned freezing
Melo 2012	Fluoxetine	20 mg/kg	Chronic [daily, 19x]	None	Rat	Plus-maze discriminative avoidance task
Xiong 2015 (1)	Fluoxetine	10 mg/kg	Chronic [daily, 33x]	Maternal separation	Rat	Conditioned freezing
Xiong 2015 (2)	Fluoxetine	10 mg/kg	Chronic [daily, 31x]	None	Rat	Conditioned freezing

Supplementary figure 4A. Forest plot of extinction learning to context with corresponding study characteristics per experiment.

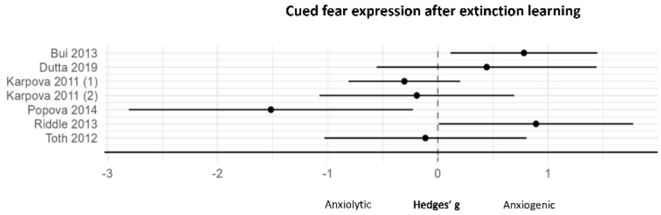

Study	Type of SSRI	Dose	Frequency	Disease induction	Species	Type of test
Bui 2013	Escitalopram	10 mg/day	Chronic [daily, 14x]	None	Human	Conditioned freezing
Dutta 2019	Paroxetine	5 mg/kg	Subchronic [daily, 3x]	None	Rat	Conditioned freezing
Karpova 2011 (1)	Fluoxetine	10 mg/kg	Chronic [daily, 25x]	None	Mouse	Conditioned freezing
Karpova 2011 (2)	Fluoxetine	10 mg/kg	Chronic [daily, 16x]	None	Mouse	Conditioned freezing
Popova 2014	Fluoxetine	10.26 mg/kg	Chronic [daily, 16x]	None	Mouse	Conditioned freezing
Riddle 2013	Fluoxetine	18 mg/kg	Chronic [daily, 24x]	None	Mouse	Conditioned freezing
Toth 2012	Fluoxetine	10 mg/kg	Acute	None	Rat	Conditioned freezing

Supplementary figure 4B. Forest plot of cued fear expression after extinction learning with corresponding study characteristics per experiment.

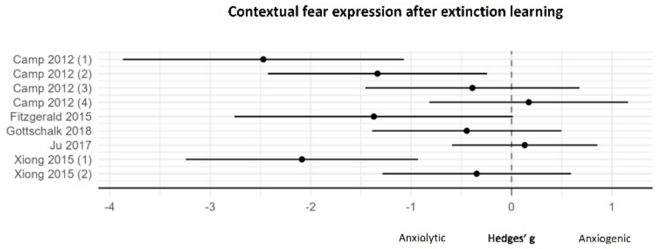

Study	Type of SSRI	Dose	Frequency	Disease induction	Species	Type of test
Camp 2012 (1)	Fluoxetine	10 mg/kg	Chronic [daily, 23x]	None	Mouse	Conditioned freezing
Camp 2012 (2)	Fluoxetine	10 mg/kg	Chronic [daily, 23x]	None	Mouse	Conditioned freezing
Camp 2012 (3)	Fluoxetine	10 mg/kg	Chronic [daily, 23x]	None	Mouse	Conditioned freezing
Camp 2012 (4)	Fluoxetine	10 mg/kg	Chronic [daily, 23x]	None	Mouse	Conditioned freezing
Fitzgerald 2015	Fluoxetine	10 mg/kg	Chronic [daily, 22x]	None	Mouse	Conditioned freezing
Gottschalk 2018	Fluoxetine	10 mg/kg	Chronic [daily, 29x]	None	Rat	Conditioned freezing
Ju 2017	Fluoxetine	10 mg/kg	Chronic [daily, 16x]	None	Mouse	Conditioned freezing
Xiong 2015 (1)	Fluoxetine	10 mg/kg	Chronic [daily, 44x]	Maternal separation	Rat	Conditioned freezing
Xiong 2015 (2)	Fluoxetine	10 mg/kg	Chronic [daily, 44x]	None	Rat	Conditioned freezing

Supplementary figure 4C. Forest plot of contextual fear expression after extinction learning with corresponding study characteristics per experiment.
