# Supplementary material for: The effect of SSRIs on fear learning: a systematic review and meta-analysis
Source: Psychopharmacology (Berl). 2023 Feb 27;240(11):2335–59. doi: 10.1007/s00213-023-06333-7 (PMC10593621; doi:10.1007/s00213-023-06333-7)
Supplement: Supplementary file 7 — Supplementary file7 (PDF 168 KB) [file 213_2023_6333_MOESM7_ESM.pdf]

# The effect of SSRIs on fear learning: a systematic review and meta-analysis

Psychopharmacology

Elise J Heesbeen, Elisabeth Y Bijlsma, P Monika Verdouw, Caspar van Lissa, Carlijn Hooijmans, Lucianne Groenink

Corresponding author: Lucianne Groenink, l.groenink@uu.nl

**Supplementary file S7. Publication bias visualized by funnel plots of the studied fear learning processes**

Risk of bias across studies

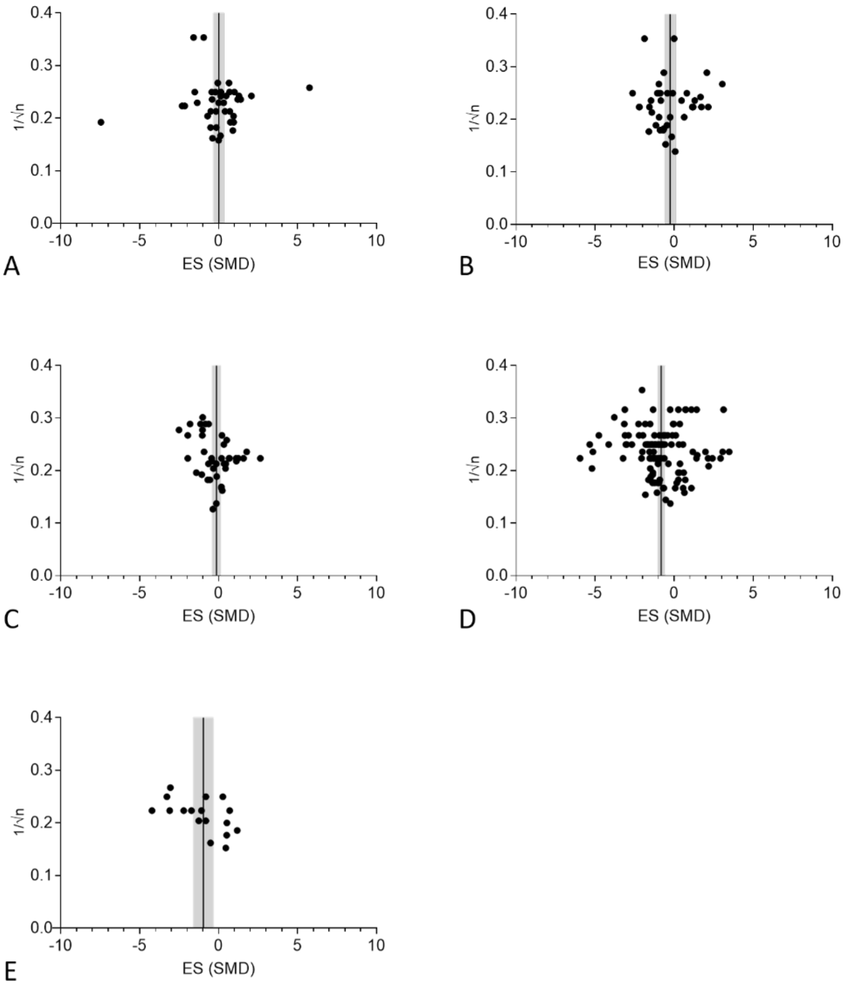


*Supplementary figure S5. Publication bias visualized by funnel plots of the following fear learning processes: A) acquisition learning to cue, B) acquisition learning to context, C) cued fear expression after acquisition learning, D) contextual fear expression after acquisition learning, E) extinction learning to cue. The vertical black line represents the SMD of the summary effect, the grey areas represent the corresponding 95% confidence intervals. ES = effect estimate, SMD = standardized mean difference.*
