# Supplementary material for: The effect of SSRIs on fear learning: a systematic review and meta-analysis
Source: Psychopharmacology (Berl). 2023 Feb 27;240(11):2335–59. doi: 10.1007/s00213-023-06333-7 (PMC10593621; doi:10.1007/s00213-023-06333-7)
Supplement: Supplementary file 8 — Supplementary file8 (PDF 502 KB) [file 213_2023_6333_MOESM8_ESM.pdf]

The effect of SSRIs on fear learning: a systematic review and meta-analysis

Psychopharmacology

Elise J Heesbeen, Elisabeth Y Bijlsma, P Monika Verdouw, Caspar van Lissa, Carlijn Hooijmans, Lucianne Groenink

Corresponding author: Lucianne Groenink, l.groenink@uu.nl

Supplementary file S8. Trim and Fill analysis for the studied fear learning processes

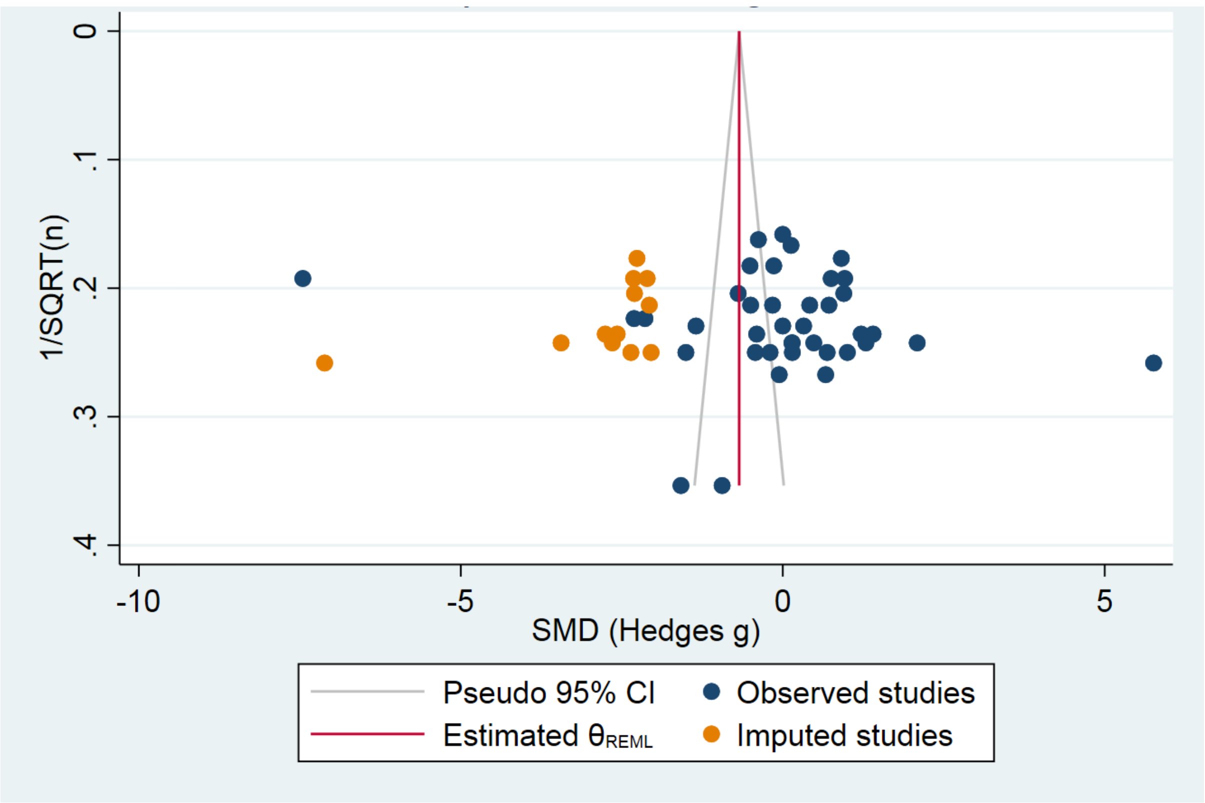

Supplementary figure 6A. Trim and Fill analysis for the fear learning process acquisition learning to cue.

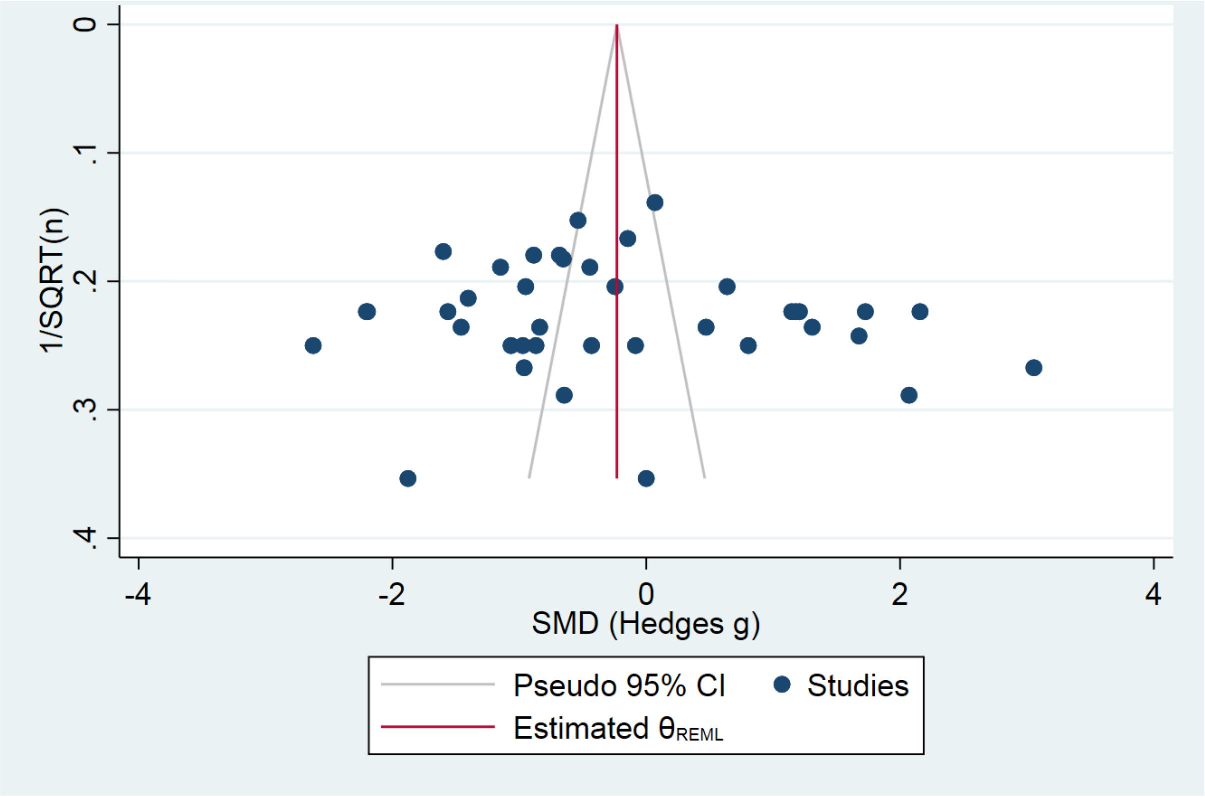

Supplementary figure 6B. Trim and Fill analysis for the fear learning process acquisition learning to context.

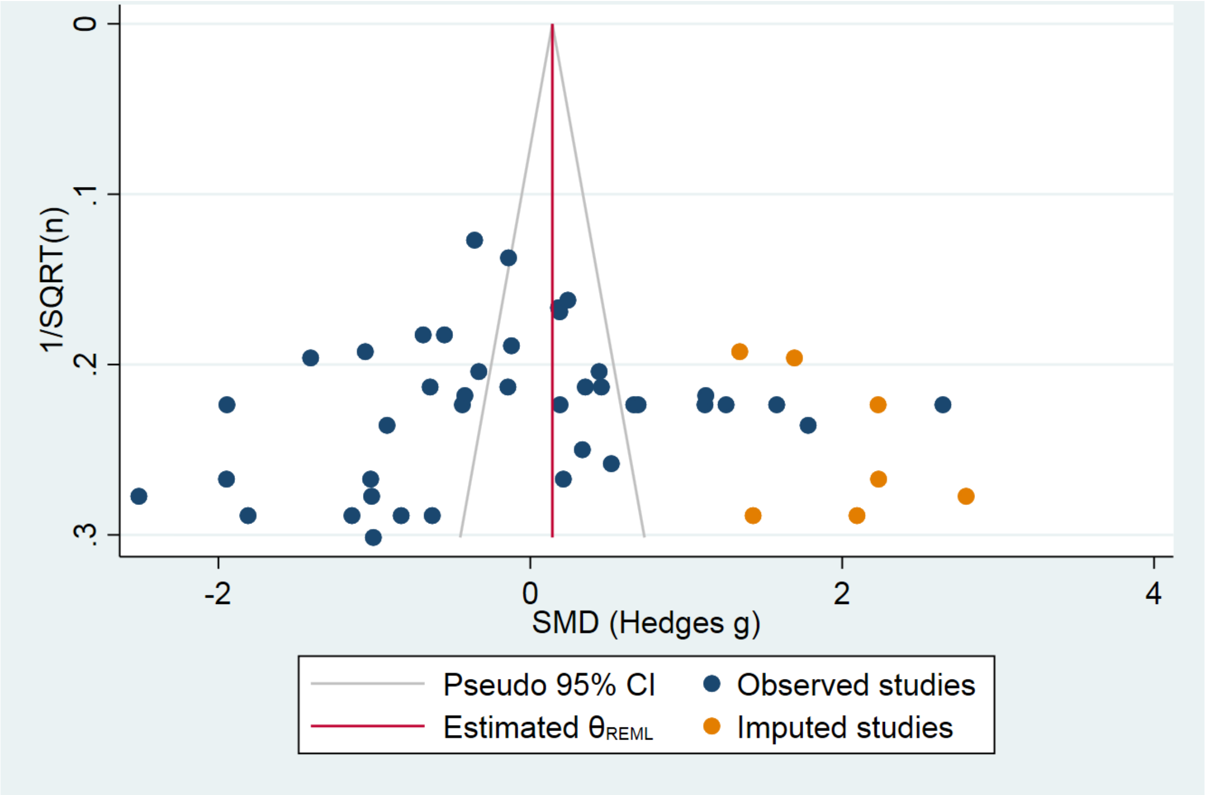

Supplementary figure 6C. Trim and Fill analysis for the fear learning process cued fear expression after acquisition learning.

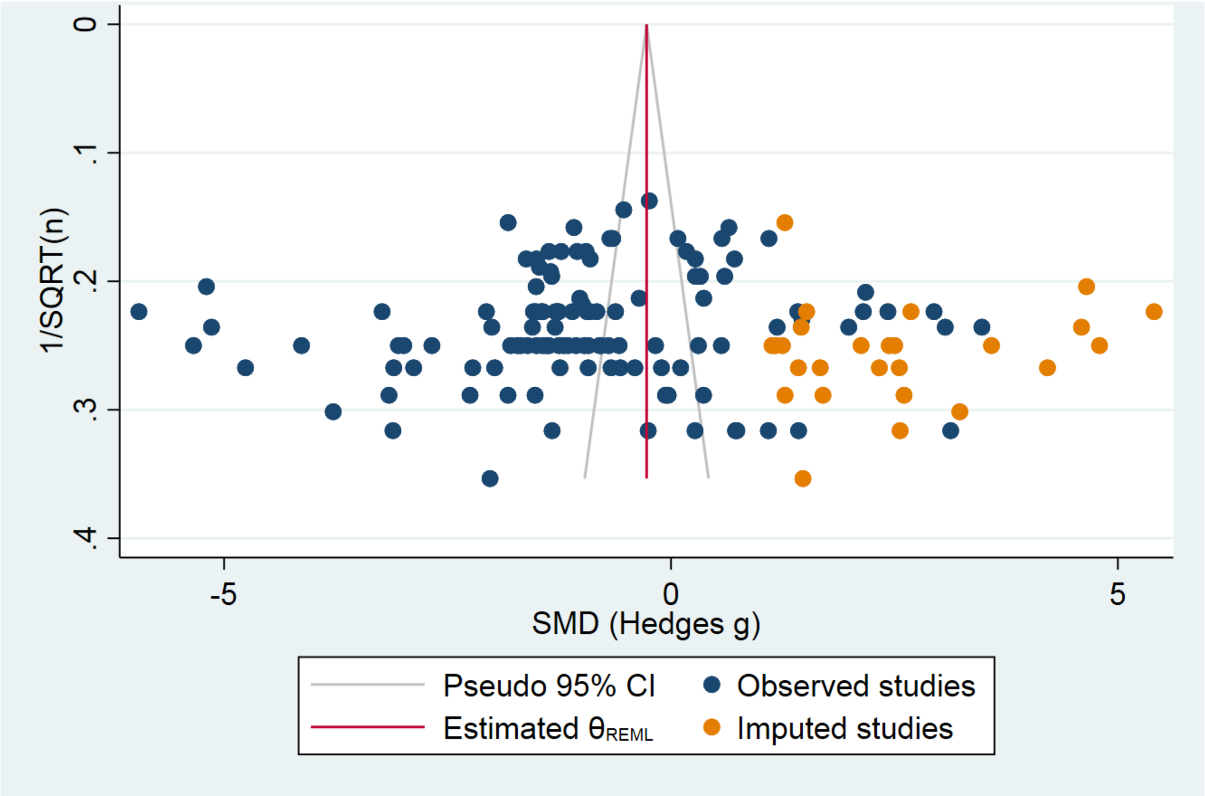

Supplementary figure 6D. Trim and Fill analysis for the fear learning process contextual fear expression after acquisition learning.

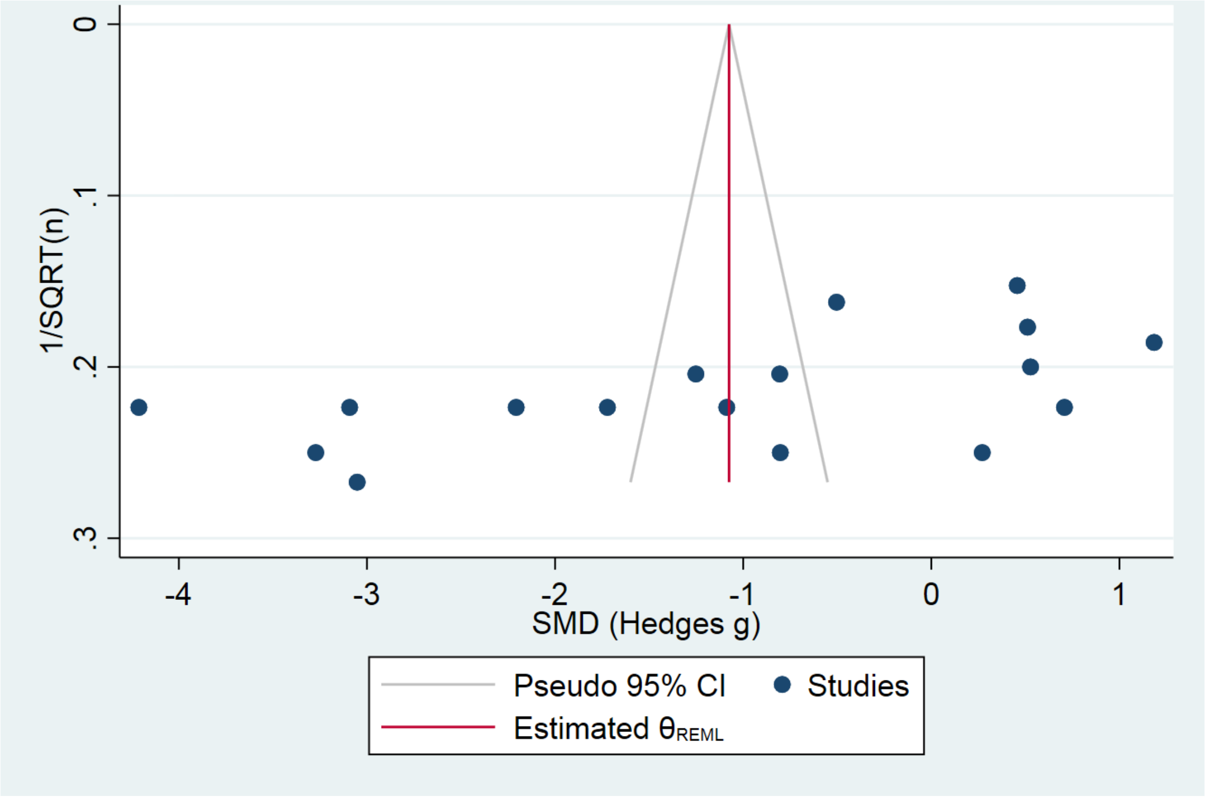

Supplementary figure 6E. Trim and Fill analysis for the fear learning process extinction learning to cue.
